# Five New Indole Alkaloid Derivatives from Deep-Sea Fungus *Aspergillus fumigatus* AF1

**DOI:** 10.3390/md23010004

**Published:** 2024-12-25

**Authors:** Lai-Hui Dai, Gao-Rong Zhang, Yang-Hui Ou, Xiao-Jing Liu, Hong-Liang Yao, Wen-Hao Hu, Hou-Jin Li, Wen-Jian Lan

**Affiliations:** 1 School of Pharmaceutical Sciences, GBRCE for Functional Molecular Engineering, Sun Yat-sen University, Guangzhou 510006, China; dailh5@mail2.sysu.edu.cn (L.-H.D.); zhanggr8@mail2.sysu.edu.cn (G.-R.Z.); cpulxj1003@163.com (X.-J.L.); huwh9@mail.sysu.edu.cn (W.-H.H.); 2 Guangxi Collaborative Innovation Center of Modern Sericulture and Silk, Hechi University, Hechi 546300, China; 3 Guangdong Key Laboratory of Animal Conservation and Resource Utilization, Guangdong Public Laboratory of Wild Animal Conservation and Utilization Institute of Zoology, Guangdong Academy of Sciences, Guangzhou 510260, China; ouyh0807@gmail.com (Y.-H.O.); yaohl@giz.gd.cn (H.-L.Y.); 4 School of Chemistry, Sun Yat-sen University, Guangzhou 510006, China

**Keywords:** deep-sea-derived fungus, *Aspergillus fumigatus*, structural identification, anti-pulmonary fibrosis activity

## Abstract

One new gliotoxin derivative fumianthrogliotoxin (**1**), one new indoquizoline alkaloid *N*3-(methyl propionate) indoquizoline (**2**), and three novel indole alkaloids, anthroxyindole (**3**), (±)-asperfumiindole A (**4**), and (±)-asperfumiindole B (**5**), together with 16 known compounds (**6**–**21**), were isolated from the culture of deep-sea derived fungus *Aspergillus fumigatus* AF1. Their chemical structures and absolute configurations were determined through the analysis of NMR data in combination with electronic circular dichroism (ECD) calculations and other spectroscopic analyses. Compounds **2**–**11** and **13**–**21** were evaluated for anti-pulmonary fibrosis activity. Compounds **8** and **13** displayed significant downregulation of the mRNA expression levels of all three molecular markers (COL1A1, α-SMA and FN1), with compound **13** exhibiting the best performance among all the tested compounds.

## 1. Introduction

The deep-sea environment accounts for 95% of the ocean, which covers 71% of the Earth’s surface area. Thus, the deep-sea is an important part of the marine system. Marine fungi are important components of marine microorganisms and have been recognized as important sources of structurally novel and biologically active metabolites [1,2]. Compared with other source microorganisms, deep-sea-derived fungi possess unique genetic backgrounds and metabolic pathways due to their living environments characterized by high salinity, high pressure, and low temperature [3]. Therefore, deep-sea-derived fungi have a greater potential to produce compounds with distinctive chemical structures and valuable pharmaceutical activities [4]. The investigation of the secondary metabolites from deep-sea-derived fungi may lead to the discovery of structurally novel compounds with pharmaceutical activity.

The genus *Aspergillus* is widely distributed and has drawn attention because of significant biological activities and novel metabolite structures [5,6,7]. It has been reported that alkaloids are also among the major classes of metabolites produced by *Aspergillus* species, possessing a variety of pharmaceutical activities, such as anti-inflammatory, antimicrobial, and cytotoxic properties [6,7,8]. The alkaloids from marine-derived fungi of *Aspergillus* are classified into diketopiperazine, quinazoline, quinoline, and indole alkaloids based on their structural features [7,9]. Fumiquinazoline is a type of indole quinazoline alkaloid featuring a pyrazino [2,1-b] quinazoline-3,6-dione core binding with an indole system [10] and it was first obtained from the marine-derived fungus *Aspergillus fumigatus* [11,12,13]. Indole alkaloids contain an indole nucleus, which is considered a “privileged structure” in pharmaceutical activities. It plays an important role in antimicrobial treatment [14,15,16]. Thus, this type of alkaloids has attracted attention among researchers.

Pulmonary fibrosis (PF), a chronic, progressive, and predominantly fatal disease, has become a significant health concern in the post-pandemic recovery period following COVID-19 [17]. Despite the multidisciplinary approaches employed in the treatment of PF, no definitive therapy for post-inflammatory pulmonary fibrosis following COVID-19 infection has been identified [18]. Studies have shown that indole alkaloids can moderate the TGF-β signaling pathway and exhibit excellent anti-pulmonary fibrosis activity, making them as potential lead drugs for the treatment of pulmonary fibrosis [19].

**Figure 1:**
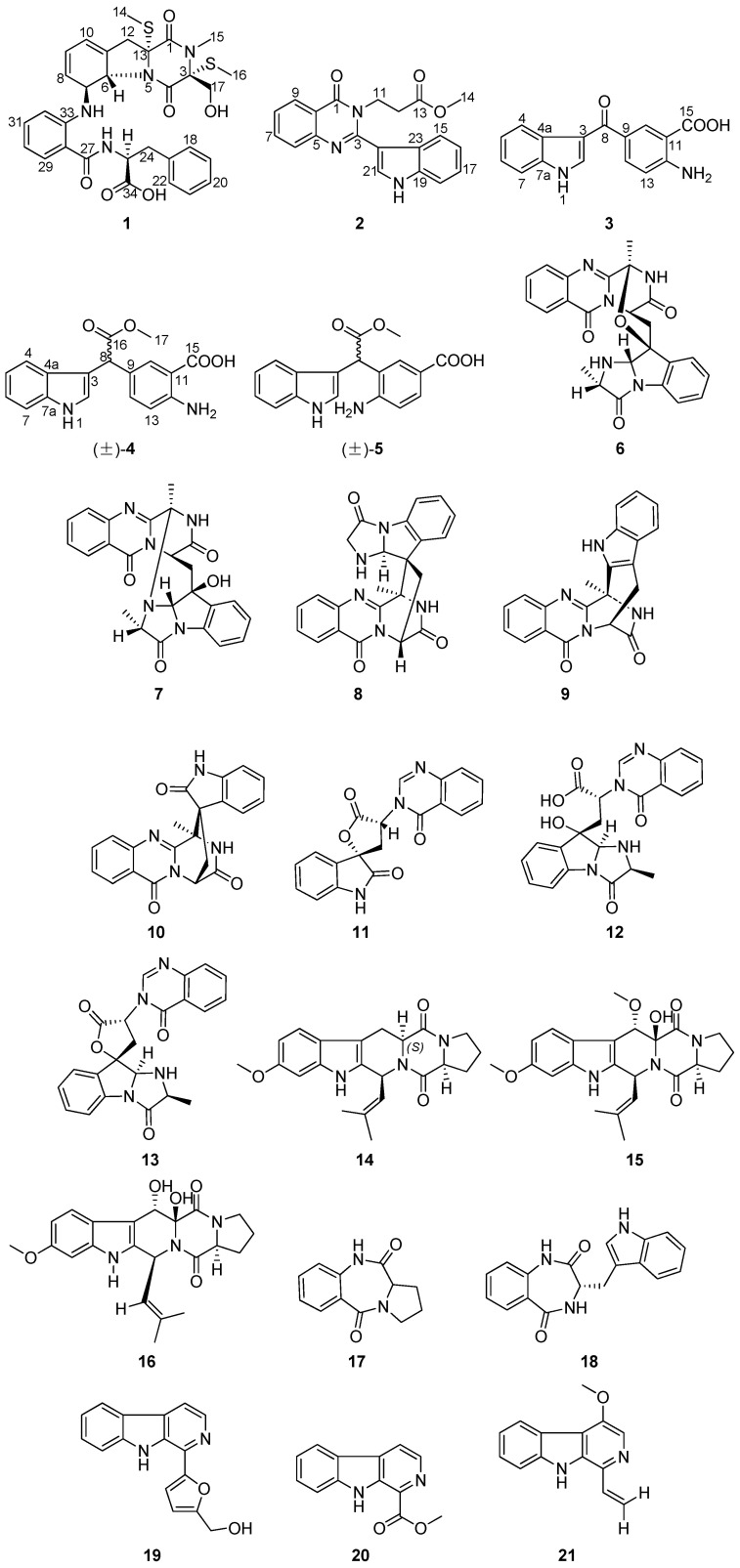
Chemical structures of compounds **1**–**21**.

Biogenetically, alkaloids are derived from amino acids, which undergo through various biosynthetic processes to form structurally diverse compounds [20]. Our previous studies demonstrated the efficacy of an amino acid-directed strategy in promoting the accumulation of indole alkaloids in fungi [21,22]. As part of our ongoing research on bioactive alkaloids of the genus *Aspergillus*, an amino acid-directed strategy was employed to culture *Aspergillus fumigatus* AF1, a fungal strain collected at a depth of 3300 m in the northern basin of the South China Sea, by adding seven types of amino acids. Herein, the isolation, structural identification, and anti-pulmonary fibrosis activity of compounds **1**–**21** (Figure 1) are described.

## 2. Results and Discussion

### 2.1. Structural Elucidation

**Figure 2:**
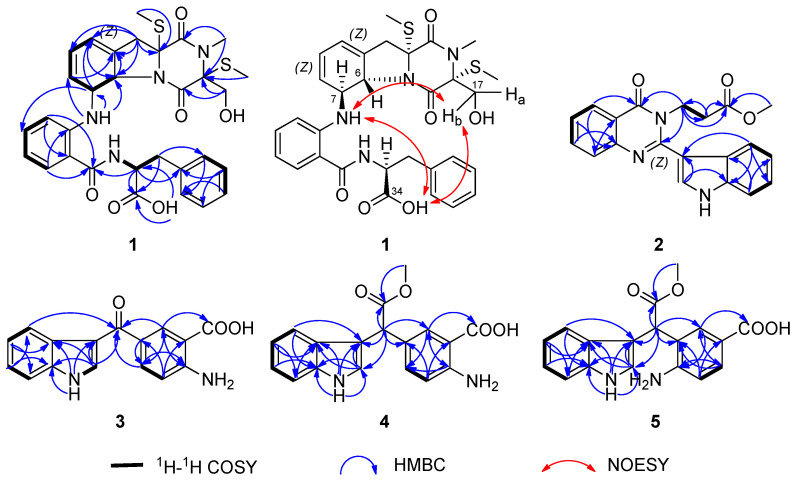
Key ^1^H-^1^H COSY and HMBC correlations of **1**–**5**, and NOESY correlations of **1**.

Compound **1** was isolated as a pale green solid. The molecular formula was revealed to be C_31_H_34_N_4_O_6_S_2_ with HR-ESI-MS at *m/z* 645.1813 [M + Na]^+^ (calcd for C_31_H_34_N_4_O_6_S_2_Na^+^, 645.1812), indicating 17 degrees of unsaturation. The NMR spectral data (Table 1, Figures S2–S8) revealed the presence of a monosubstituted benzene ring [*δ*_H_ 7.31 (1H, d, *J* = 7.0 Hz, H-18), 7.25 (1H, dd, *J* = 7.5, 7.0 Hz, H-19), 7.20 (1H, dd, *J* = 7.5, 7.5 Hz, H-20), 7.25 (1H, dd, *J* = 7.5, 7.0 Hz, H-21), 7.31 (1H, d, *J* = 7.0 Hz, H-22); *δ_C_* 129.3 (C-18), 128.2 (C-19), 126.3 (C-20), 128.2 (C-21), 129.3 (C-22), and 137.9 (C-23)], a 1,2-disubstituted benzene ring [*δ*_H_ 7.66 (1H, dd, *J* = 7.5, 1.5 Hz, H-29), 7.20, (1H, dd, *J* = 7.5, 7.5 Hz, H-30), 7.50 (1H, ddd, *J* = 8.0, 7.5, 1.5 Hz, H-31), 7.10 (1H, d, *J* = 8.0 Hz, H-32); *δ_C_* 126.3 (C-28), 130.3 (C-29), 124.0 (C-30), 132.2 (C-31), 121.0 (C-32), 136.8 (C-33)], two olefinic bonds [*δ*_H_ 5.64 (1H, d, *J* = 9.5 Hz, H-8), *δ*_H_ 5.90 (1H, m, H-9), *δ*_H_ 6.00 (1H, m, H-10); *δ_C_* 130.6 (C-8), *δ_C_* 123.5 (C-9), *δ_C_* 119.3 (C-10), *δ_C_* 133.1 (C-11)], three methyl groups [*δ*_H_ 2.19 (3H, s, H-14), 2.99 (3H, s, H-15), 2.20 (3H, s, H-16); *δ_C_* 14.7 (C-14), 28.3 (C-15), 12.8 (C-16)], three methylene groups [*δ*_H_ 2.85, 3.12 (2H, m, H-12), 3.73 (1H, dd, *J* = 11.0, 3.0 Hz, H-17), 4.06 (1H, d, *J* = 11.0 Hz, H-17), 2.81, 3.12 (2H, m, H-24); *δ_C_* 38.4 (C-12), 63.0 (C-17), 33.3 (C-24)], four carbonyl groups [*δ_C_* 165.2 (C-1), 166.3 (C-4), 167.7, (C-27), 171.3 (C-34)], and two hydroxyl groups [*δ*_H_ 5.47 (1H, d, *J* = 3.0 Hz, 17-OH), 10.41 (1H, brs, 34-OH)]. Comparing the spectroscopic data (^1^H and ^13^C NMR, DEPT 135, ^1^H−^1^H COSY, HSQC, HMBC, and HR-ESI-MS) with the published data of the compounds bisdethiobis (methylthio) gliotoxin and dehydroxybisdethiobis (methylthio) gliotoxin [23,24], compound **1** has a similar fragment structure to bisdethiobis (methylthio) gliotoxin, except that the hydroxyl group at C-6 is replaced by an imide group, according to the molecular formula of compound **1**. The ^1^H−^1^H COSY and HMBC correlations also proved that the fragment of bisdethiobis (methylthio) gliotoxin was connected to the imide group (7-NH) (Figure 2). Furthermore, the ^1^H−^1^H COSY correlations between H-24 (*δ*_H_ 2.81, m; *δ*_H_ 3.12, m) and H-25 (1H, *δ*_H_ 3.89, dt, *J* = 11.0, 6.5 Hz), H-25 and the active hydrogen H-26 (*δ*_H_ 8.51, 1H, brd, *J* = 6.5 Hz), along with the HMBC correlations from H-24 to C-18 (*δ_C_* 129.3)/C-23 (*δ_C_* 137.9)/C-34 (*δ*_C_ 171.3), H-25 to C-34 (*δ*_C_ 171.3)/C-23 (*δ_C_* 137.9), and from 34-OH (*δ*_H_ 10.41, brs) to C-34 (*δ*_C_ 171.3)/C-25 (*δ*_C_ 53.9), indicated that the monosubstituted benzene ring was linked to –CH_2_-CH(C=O)-NH– through C-23 and composed the phenylalanine fragment (Figure 2). The ^1^H−^1^H COSY correlations of H-29/H-30/H-31/H-32 were observed. The HMBC correlations from H-29 to C-27 (*δ*_C_ 167.7)/C-33 (*δ*_C_ 136.8), from H-32 to C-27 (*δ*_C_ 167.7)/C-28 (*δ*_C_ 126.3), and from H-25 to C-27 (*δ*_C_ 167.7), indicated that the 1,2-disubstituted benzene ring was linked to the –CH_2_-CH(C=O)-NH– of the phenylalanine fragment though carbonyl C-27 (*δ*_C_ 167.7) (Figure 2). Finally, the two main sections were linked by 7-NH (*δ*_H_ 5.47, m) with the HMBC signals from 7-NH to C-6 (*δ_C_* 69.0)/C-7 (*δ_C_* 73.7)/C-8 (*δ_C_* 130.6) and H-7 to C-31 (*δ_C_* 132.2) (Figure 2). Thus, the planar structure of 1 was established as shown in Figure 1.

**Figure 3:**
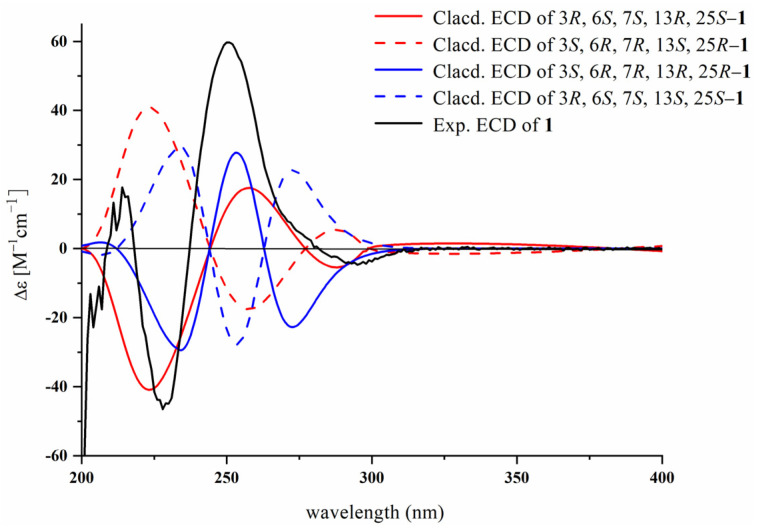
Experimental and calculated ECD spectra of **1**.

The relative configuration of **1** was deduced according to the NOESY experiment. Initially, the NOESY correlations between OH-34 (1H, *δ*_H_ 10.41, brs) and NH-7/H_b_-17 and between NH-7 and H_b_-17 suggested that OH-34, NH-7, and CH_2_OH shared the same orientation. Additionally, the relatively large coupling constants of H-6/H-7 (^3^*J*_H-6/H-7_ = 14.0 Hz) indicated that they are in opposite directions. Therefore, the four possible configurations of **1** were (3*R*, 6*S*, 7*S*, 13*R*, 25*S*)-**1**, (3*S*, 6*R*, 7*R*, 13*R*, 25*R*)-**1**, (3*S*, 6*R*, 7*R*, 13*S*, 25*R*)-**1**, and (3*R*, 6*S*, 7*S*, 13*S*, 25*S*)-**1**. According to the comparisons of the experimental and calculated ECD curves of the four possible configurations, the experimental results matched well with those calculated for (3*R*, 6*S*, 7*S*, 13*R*, 25*S*)-**1** and (3*S*, 6*R*, 7*R*, 13*R*, 25*R*)-**1** (Figure 3). In addition, the calculated optical rotation data of the configurations (3*R*, 6*S*, 7*S*, 13*R*, 25*S*)-**1** and (3*S*, 6*R*, 7*R*, 13*R*, 25*R*)-**1** were +228.195 and −577.239 (Table S1). Furthermore, the experimental optical rotation of compound **1** was +118.15, which is in better agreement with the calculated data of (3*R*, 6*S*, 7*S*, 13*R*, 25*S*)-**1**. Therefore, the absolute configuration of **1** was defined as (3*R*, 6*S*, 7*S*, 13*R*, 25*S*)-**1** according to the above analysis. Herein, compound **1** was identified and named fumianthrogliotoxin.

Compound **2** was obtained as a pale green particulate. Its molecular formula was determined to be C_20_H_17_N_3_O_3_ based on a deprotonated molecular ion peak at *m/z* 346.1196 [M − H]^−^ (calcd for C_20_H_16_N_3_O_3_^−^, 346.1197) in HR-ESI-MS with 14 degrees of unsaturation. The ^1^H NMR spectrum (Table 2) indicated the presence of two 1,2-disubstituted benzene rings [*δ*_H_ 7.66 (1H, d, *J* = 8.0 Hz, H-15), 7.22 (1H, dd, *J* = 8.0, 7.5 Hz, H-16), 7.26 (1H, dd, *J* = 8.0, 7.5 Hz, H-17), 7.39 (1H, d, *J* = 8.0 Hz, H-18); *δ*_H_ 7.77 (2H, m, H-6, H-7), 7.50 (1H, m, H-8), 8.34 (1H, d, *J* = 8.0 Hz, H-9)], one olefinic bond (*δ*_H_ 7.52, 1H, s, H-21), and one methoxy (*δ*_H_ 3.54, s, 3H). The ^13^C NMR data (Table 2) and HSQC data displayed signals for two carbonyls [*δ*_C_ 162.8 (C-1), 171.4 (C-13)], two disubstituted benzene rings [*δ*_C_ 147.7 (C-5), 127.0 (C-6), 134.7 (C-7), 127.3 (C-8), 126.9 (C-9), 120.6 (C-10); *δ*_C_ 119.9 (C-15), 119.9 (C-16), 121.7 (C-17), 123.5 (C-18), 135.8 (C-19), 126.2 (C-23)], one methoxy (*δ*_C_ 51.9), and one olefinic [*δ*_C_ 125.8 (C-21), 111.2 (C-22)]. The following data supported the presence of an indole residue: eight characteristic aromatic/olefinic carbons (C-15−C-23); the ^1^H−^1^H COSY correlations between *δ*_H_ 7.66 (1H, d, *J* = 8.0 Hz, H-15) and *δ*_H_ 7.22 (1H, dd, *J* = 8.0, 7.5 Hz, H-16), *δ*_H_ 7.26 (1H, dd, *J* = 8.0, 7.5 Hz, H-17), and *δ*_H_ 7.39 (1H, d, *J* = 8.0 Hz, H-18); HMBC correlations from *δ*_H_ 7.66 (H-15) to *δ*_C_ 121.7 (C-17)/*δ*_C_ 135.8 (C-19)/*δ*_C_ 111.2 (C-22), from *δ*_H_ 7.39 (H-18) to *δ*_C_ 119.9 (C-16)/*δ*_C_ 126.2 (C-23), and from *δ*_H_ 7.52 (1H, H-21) to *δ*_C_ 135.8 (C-19)/*δ*_C_ 111.2 (C-22); and the NH signal (*δ*_H_ 9.07, 20-NH). Additionally, the ^1^H−^1^H COSY correlation of *δ*_H_ 4.55 (2H, t, *J* = 7.6 Hz, H-11) and *δ*_H_ 2.71 (2H, t, *J* = 7.6 Hz, H-12) and the HMBC correlations from *δ*_H_ 4.55 (H-11) to *δ*_C_ 33.2 (C-12) and *δ*_C_ 171.4 (C-13), from *δ*_H_ 2.71 (H-12) to *δ*_C_ 42.0 (C-11) and *δ*_C_ 171.4 (C-13), and from *δ*_H_ 3.54 (3H, s, OCH_3_-14) to *δ*_C_ 171.4 (C-13), indicated the presence of the fragment –CH_2_-CH_2_ (CO)-OCH_3_. The ^1^H−^1^H COSY correlations of *δ*_H_ 7.77 (1H, m, H-7)/7.50 (1H, m, H-8) and 7.50 (H-8)/8.34 (1H, d, *J* = 8.0 Hz, H-9) were observed. Combined with the molecular formula and unsaturation, the HMBC correlations from *δ*_H_ 7.77 (H-7) to *δ*_C_ 147.7 (C-5), from *δ*_H_ 7.50 (H-8) to *δ*_C_ 127.0 (C-6), from *δ*_H_ 7.77 (1H, m, H-6) to *δ*_C_ 120.6 (C-10), and from *δ*_H_ 8.34 (1H, d, *J* = 8.0 Hz, H-9) to *δ*_C_ 162.8 (C-1)/*δ*_C_ 147.7 (C-5) indicated that the presence of the fragment quinazolin-4(3*H*)-one [8]. In addition, the HMBC correlations from *δ*_H_ 4.55 (1H, t, *J* = 7.6 Hz, H-11) to *δ*_C_ 162.8 (C-1)/*δ*_C_ 151.9 (C-3) were observed. Therefore, the two fragments of -CH_2_-CH_2_(CO)-OCH_3_ and quinazolin-4(3*H*)-one are connected by a nitrogen atom and comprise methyl 3-(4-oxoquinazolin-3(4*H*)-yl) propanoate [8]. According to the molecular formula and the unsaturation of compound **2**, the fragment of methyl 3-(4-oxoquinazolin-3(4*H*)-yl) propanoate is linked to the indole residue through C-3 (*δ*_C_ 151.9) and C-22 (*δ*_C_ 111.2). Thus, the structure of **2** was established as shown in Figure 1 and named *N*3-(methyl propionate) indoquizoline.

Compound **3** was isolated as a light-brown solid. The HR-ESI-MS analysis of **3** showed a molecular ion at *m*/*z* 303.0738 [M + Na]^+^ (calcd for C_16_H_12_N_2_O_3_Na^+^, 303.0740), which indicated 12 degrees of unsaturation. The ^1^H NMR data (Table 3) indicated the presence of one 1,2-disubstituted benzene ring [*δ*_H_ 8.15 (1H, d, *J* = 7.0 Hz, H-4), 7.17 (1H, dd, *J* = 7.5, 7.0 Hz, H-5), 7.21 (1H, dd, *J* = 7.5, 7.0 Hz, H-6), 7.50 (1H, d, *J* = 7.5 Hz, H-7)] and one 1,3,4-trisubstituted benzene ring [*δ*_H_ 8.36 (1H, *s*, H-10), 6.71 (1H, d, *J* = 7.5, Hz, H-13), 7.63 (1H, d, *J* = 7.5 Hz, H-14)]. The ^13^C NMR data (Table 4) and HMBC data displayed signals for two carbonyls [*δ*_C_ 188.2 (C-8), 171.0 (C-15)], one disubstituted benzene ring [*δ*_C_ 126.7 (C-4a), 121.4 (C-4), 121.1 (C-5), 122.6 (C-6), 112.0 (C-7), 136.4 (C-7a)], one trisubstituted benzene ring [*δ*_C_ 126.1 (C-9), 134.0 (C-10), 126.1 (C-11), 153.8 (C-12), 115.2 (C-13), 132.2 (C-14)], and one olefinic [*δ*_C_ 133.2 (C-2), 115.1 (C-3)]. The following indicated the presence of a 3-substituted indole group: eight characteristic aromatic/olefinic carbons (*δ*_C_ 133.2, 115.1, 126.7, 121.4, 121.1, 122.6, 112.0, 136.4); the ^1^H−^1^H COSY correlations of *δ*_H_ 8.15 (1H, d, *J* = 7.0 Hz, H-4)/7.17 (1H, dd, *J* = 7.5, 7.0 Hz, H-5), 7.21 (1H, dd, *J* = 7.5, 7.0 Hz, H-6)/7.50 (1H, d, *J* = 7.5 Hz, H-7), and 7.86 (1H, s, H-2)/11.94 (NH-1, s); and the HMBC correlations from H-5 to *δ*_C_ 126.7 (C-4a)/*δ*_C_ 136.4 (C-7a), *δ*_H_ 7.21 (H-6) to *δ*_C_ 136.4 (C-7a)/*δ*_C_ 121.4 (C-4), from *δ*_H_ 7.86 (H-2) to *δ*_C_ 115.1 (C-3)/*δ*_C_ 126.7 (C-4a)/*δ*_C_ 136.4 (C-7a), and from *δ*_H_ 11.94 (NH-1, s) to *δ*_C_ 115.1 (C-3)/*δ*_C_ 126.7 (C-4a)/*δ*_C_ 136.4 (C-7a). The HMBC correlations from *δ*_H_ 8.15 (H-4)/*δ*_H_ 7.86 (H-2) to *δ*_C_ 188.2 (C-8), revealed that the carbonyl group is linked to a 3-substituted indole group through C-3 (*δ*_C_ 115.1). In addition, the presence of the fragment anthranilic acid residue was deduced by according to the ^1^H−^1^H COSY correlations of *δ*_H_ 6.71 (1H, d, *J* = 7.5 Hz, H-13)/7.63 (1H, d, *J* = 7.5 Hz, H-14), and the HMBC correlations from *δ*_H_ 6.71 (1H, d, *J* = 7.5 Hz, H-13) to *δ*_C_ 126.1 (C-9)/*δ*_C_ 126.1 (C-11), from *δ*_H_ 7.63 (1H, d, *J* = 7.5 Hz, H-14) to *δ*_C_ 134.0 (C-10)/*δ*_C_ 153.8 (C-12), and from *δ*_H_ 8.36 (H-10) to *δ*_C_ 153.8 (C-12)/*δ*_C_ 132.2 (C-14)/*δ*_C_ 171.0 (C-15), along with the molecular formula and the unsaturation. Finally, the HMBC correlations from *δ*_H_ 7.86 (H-2)/*δ*_H_ 8.15 (H-4)/*δ*_H_ 8.36 (H-10)/*δ*_H_ 7.63 (H-14) to C-8 (*δ*_C_ 188.2) revealed that the two fragments are connected by the carbonyl group (*δ*_C_ 188.2). Thus, the structure of **3** was established as shown in Figure 1 and named anthroxyindole.

**Figure 4:**
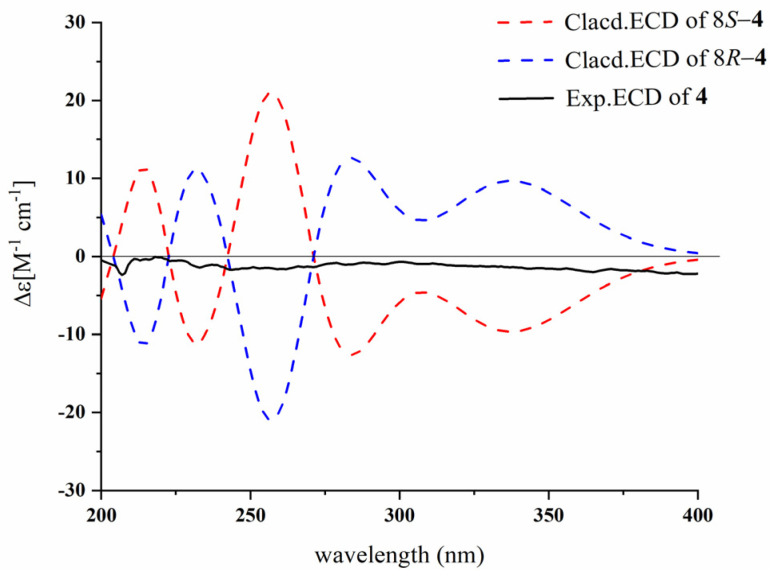
Experimental and calculated ECD spectra of **4**.

Compound **4** was isolated as a dark-brown solid with the molecular formula of C_18_H_16_N_2_O_4_, based on the HR-ESI-MS ion at *m*/*z* 347.1002 [M + Na]^+^ (calcd for C_18_H_16_N_2_O_4_Na^+^, 347.1002). The ^1^H NMR data (Table 3) indicated the presence of one 1,2-disubstituted benzene ring [*δ*_H_ 7.35 (1H, d, *J* = 8.0 Hz, H-4), 6.93 (1H, dd, *J* = 8.0, 7.0 Hz, H-5), 7.06 (1H, dd, *J* = 8.0, 7.0 Hz, H-6), 7.35 (1H, d, *J* = 8.0 Hz, H-7)], one 1,3,4-trisubstituted benzene ring [*δ*_H_ 7.71 (1H, s, H-10), 6.66 (1H, d, *J* = 8.0 Hz, H-13), 7.23 (1H, d, *J* = 8.0 Hz, H-14)], one methoxy (*δ*_H_ 3.66, s, 3H, OCH_3_-17), and one methine (*δ*_H_ 5.12, s, 1H, H-8). The ^13^C NMR (Table 4), DEPT 90, and HSQC data displayed signals for two carbonyls [*δ*_C_ 173.4 (C-16), 169.8 (C-15)], one disubstituted benzene ring [*δ*_C_ 126.2 (C-4a), 118.6 (C-4), 118.7 (C-5), 121.3 (C-6), 111.6 (C-7), 136.3 (C-7a)], one trisubstituted benzene ring [*δ*_C_ 125.0 (C-9), 130.8 (C-10), 110.1 (C-11), 150.5 (C-12), 116.4 (C-13), 133.8 (C-14)], one olefinic [*δ*_C_ 123.5 (C-2), 112.7 (C-3)], one methoxy (*δ*_C_ 57.0, OCH_3_-17), and one methine group (*δ*_C_ 47.1, C-8). The HMBC correlations from *δ*_H_ 3.66 (3H, s, OCH_3_-17) to *δ*_C_ 173.4 (C-16), indicated the presence of the fragment–COOCH_3_ (Figure 2). Based on the analysis of the NMR data mentioned above and the molecular formula, compound **4** has the same skeleton comprising a 3-substituted indole group and anthranilic acid residues as compound **3**. Moreover, the key correlations from *δ*_H_ 5.12 (1H, s, H-8) to *δ*_C_ 123.5 (C-2)/*δ*_C_ 112.7 (C-3)/*δ*_C_ 125.0 (C-9)/*δ*_C_ 130.8 (C-10)/*δ*_C_ 173.4 (C-16) support that the three fragments are linked by CH (*δ*_C_ 47.1, C-8) (Figure 2). Thus, the planar structure of **4** is shown in Figure 1, where it can be seen that there is a chiral carbon at C-8; however, the experimental CD spectra of compound **4** did not indicate any apparent Cotton effects (Figure 4), and its optical rotation could barely be detected, indicating that **4** might be a racemic mixture. Unfortunately, the enantiomers were not isolated due to the limited sample mass. Therefore, compound **4** was identified and named (±)-asperfumiindole A.

**Figure 5:**
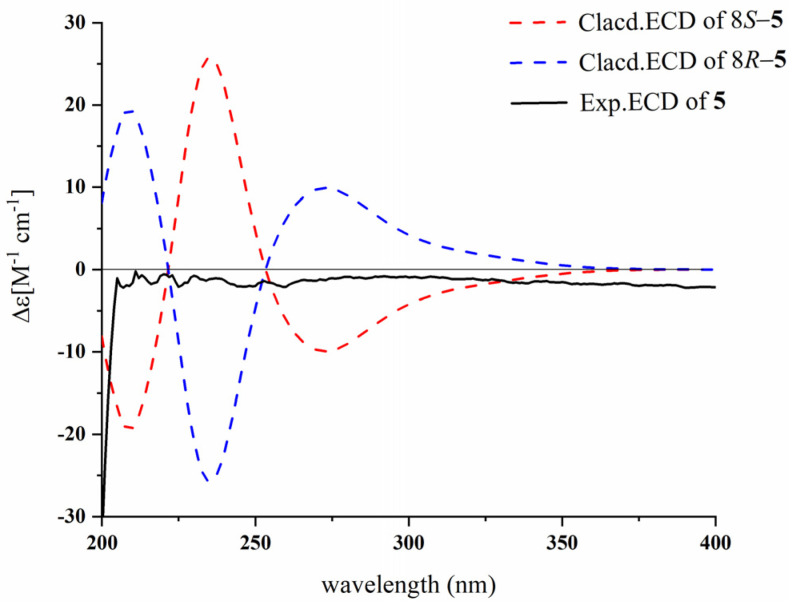
Experimental and calculated ECD spectra of **5**.

Compound **5** was obtained as a dark-brown solid. The molecular formula was established as C_18_H_16_N_2_O_4_ based on the HR-ESI-MS ion at *m*/*z* 347.1002 [M + Na]^+^ (calculated for C_18_H_16_N_2_O_4_Na^+^, 347.1002). The comparison of the NMR signals of **5** with those of **4** indicated that **5** was an analogue of **4**, with the major differences existing in the ^1^H and ^13^C NMR data of the benzene ring between **5** and **4**. In the ^1^H and ^13^C NMR spectra (Table 3 and Table 4), the chemical shifts of the aromatic ring at *δ*_H_ 7.23 (1H, d, *J* = 8.0, H-14), 6.66 (1H, d, *J* = 8.0, H-13); and *δ*_C_ 125.0, 130.8, 110.1, 150.5, 116.4, 133.8 in **4** were changed to *δ*_H_ 6.96 (1H, dd, *J* = 8.0, 2.0 Hz, H-12), 6.44 (1H, d, *J* = 8.0, H-13); and *δ*_C_ 124.0 (C-9), 131.6 (C-10), 120.1 (C-11), 129.9 (C-12), 115.2 (C-13), 149.3 (C-14) in **5**, suggesting that the substituent group is located in a different position to compose a 1,2,4-trisubstituent in **5**. The analysis of the ^1^H-^1^H COSY correlations of *δ*_H_ 6.96 (1H, dd, *J* = 8.0, 2.0 Hz, H-12)/6.44 (1H, d, *J* = 8.0 Hz, H-13) and the HMBC correlations from *δ*_H_ 6.96 (H-12) to *δ*_C_ 131.6 (C-10)/*δ*_C_ 149.3 (C-14), from *δ*_H_ 6.44 (H-13) to *δ*_C_ 124.0 (C-9)/*δ*_C_ 120.1 (C-11), and from *δ*_H_ 7.73 (1H, d, *J* = 2.0 Hz, H-10) to *δ*_C_ 129.9 (C-12)/*δ*_C_ 149.3 (C-14)/*δ*_C_ 149.3 (C-15) (Figure 2) also proved the fragment para-aminobenzoic acid. Therefore, compound **5** was deduced to have the same skeleton as **4**, except that the NH_2_ substituent group is linked to the position of the benzene ring at C-14 (*δ*_C_ 149.3) instead of C-12 (*δ*_C_ 129.9) in **5**. Thus, the structure of **5** was determined as shown in Figure 1, where it can be seen that there is a chiral carbon at C-8; however, the experimental CD spectra of compound **5** did not display any apparent Cotton effect (Figure 5), and its optical rotation could barely be detected, indicating that **5** might be a racemic mixture. Unfortunately, the enantiomers were not isolated due to the sample mass being too low. Thus, compound **5** was identified and named (±)-asperfumiindole B.

The chemical structures of the known compounds **6**–**21** were elucidated as fumiquinazoline C (**6**) [12], fumiquinazoline D (**7**) [25], spiroquinazoline (**8**) [26], fumiquinazoline J (**9**) [27], alantrypinone (**10**) [28], 3-(4-oxoquinazolin-3-yl)spiro[1*H*-indole-3,5-oxolane]-2, 20-dione (**11**) [9], fumigatoside F (**12**) [29], tryptoquivaline O (**13**) [30], fumitremorgin C (**14**) [27], cyclotryprostatin B (**15**) [31], cyclotryprostatin A (**16**) [32], cycloanthranilylproline (**17**) [33], benzodiazepinedione (**18**) [12], perlolyrine (**19**) [34], 1-methoxycarbonyl-*β*-carboline (**20**) [35], and 4-methoxy-1-vinyl-*β*-carboline (**21**) [36]. All of these compounds were identified by comparing their ^1^H and ^13^C NMR data (Figures S45–S76) with those reported in the literature.

### 2.2. Anti-Pulmonary Fibrosis Activity

**Figure 6:**
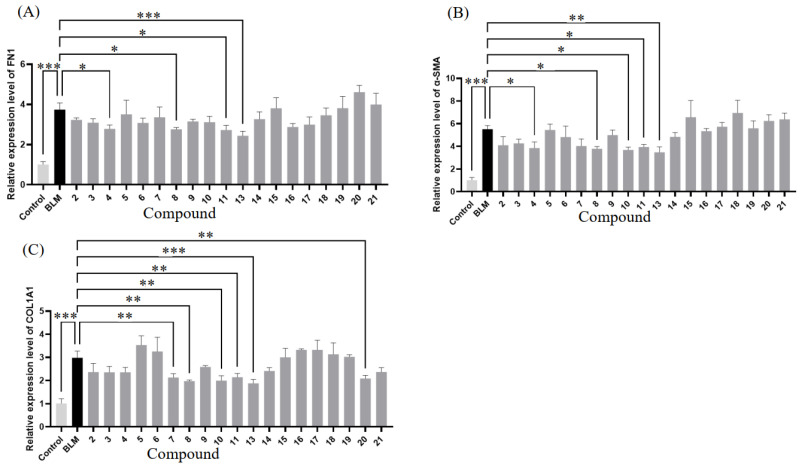
Relative mRNA expression levels of FN1 (**A**), α-SMA (**B**), and COL1A1 (**C**) in bleomycin-treated A549 cells. A549 cells were pre-treated with control (DMSO) or indicated compounds (50 μM) for 2 h, followed by stimulation with bleomycin (0.02 U/mL) for an additional 48 h. Relative mRNA expression levels of COL1A1, α-SMA, and FN1 were measured with qRT-PCR and normalized to GADPH. Data are presented as means ± SD (error bars) values. * *p* < 0.05, ** *p* < 0.01, and *** *p* < 0.001 vs. BLM group.

Compounds **2**–**11** and **13**–**21** were tested for their anti-pulmonary fibrosis activity in A549 cells using qRT-PCR. FN1, COL1A1, and α-SMA are crucial molecular markers of pulmonary fibrosis [37]. FN1 contributes to the formation of the extracellular matrix formation, COL1A1 is a key component of type I collagen, and α-SMA serves as a marker of myofibroblast activity. The abnormal expression of these markers often indicates fibrosis progression, which is closely associated with tissue stiffening and functional impairment [38]. Bleomycin is an antitumor antibiotic widely used to induce pulmonary fibrosis in animal models [39]. It causes direct lung tissue damage, triggering inflammatory responses and fibroblast activation, which ultimately leads to excessive extracellular matrix deposition, such as collagen, and fibrosis formation. The qRT-PCR results showed that the mRNA expression levels of COL1A1, α-SMA, and FN1 were significantly upregulated in the bleomycin-treated group, indicating high expression of the extracellular matrix proteins FN1 and COL1A1 and the myofibroblast marker α-SMA. These findings are closely associated with the fibroblast activation, excessive extracellular matrix deposition, and tissue stiffening observed in pulmonary fibrosis. In our study, compounds **8** and **13** showed significant downregulation of the mRNA expression levels of all three molecular markers (COL1A1, α-SMA, and FN1), with compound **13** exhibiting the best performance among all the tested compounds (Figure 6).

## 3. Materials and Methods

### 3.1. General Experimental Procedures

An Anton Paar MCP500 polarimeter was used to measure optical rotations. ECD spectra were acquired with a JASCO J-810 circular dichroism spectrometer (Applied Photophysics Ltd., Leatherhead, UK). UV and IR spectra were obtained using a Shimadzu UV-vis-NIR spectrophotometer and a Bruker Tensor-27 spectrophotometer. 1D and 2D NMR spectra were recorded on Bruker Avance IIIT 500HD and Bruker Avance II 400 spectrometers (Bruker Bio Spin AG, Industriestrasse 26, Fällanden, Switzerland). The chemical shifts relative to the residual solvent signals were as follows: CDCl_3_: *δ*_H_ 7.260 and *δ*_C_ 77.00; DMSO-*d_6_*: *δ*_H_ 2.500, and *δ*_C_ 39.52. HR-ESI-MS data were acquired using Thermo DSQ EI low-resolution and Thermo MAT95XP EI high-resolution mass spectrometers (Thermo Fisher Scientific Inc., Waltham, MA, USA). Preparative HPLC was performed by using a Shimadzu LC-20AT HPLC pump combined with an SPD-20A dual λ absorbance detector and an ODS column (250 × 20 mm; Shimadzu Corporation, Nakagyo-ku, Kyoto, Japan). Column chromatography was performed by using silica gel (SiO_2_, 200–300 mesh; Qingdao Marine Chemical Factory, Qingdao, China). Sephadex LH-20 (GE Healthcare, Chicago, IL, USA) was also utilized for column chromatography.

### 3.2. Fungal Material

The fungal strain *Aspergillus fumigatus* AF1, which had been separated from 3300 m deep sea water in the northern basin of the South China Sea, was stored in a 15% (*v*/*v*) glycerol aqueous solution at −80 °C. A voucher specimen was deposited in the School of Pharmaceutical Sciences, Sun Yat-sen University, Guangzhou, China.

### 3.3. Fermentation, Extraction, and Isolation

To stimulate the secondary metabolism in the *A. fumigatus* AF1, we employed the amino acid-directed strategy [19]. The culture with fungal mycelia was statically incubated at 28 °C for 30 days in 180 Erlenmeyer flasks (1000 mL), each containing 400 mL of sterilized GPY medium supplemented with amino acids (L-Trp 2 g/L, L-Ser 2 g/L, L-Thr 2 g/L, L-Lys 2 g/L, L-Phe 2 g/L, L-Val 2 g/L, and D,L-Met 2 g/L) and artificial sea salt (20 g/L) at pH = 7. After incubation, the fermentation liquid and mycelia were separated by with cheesecloth and then extracted five times with EtOAc and MeOH, respectively. The crude extracts of fermentation liquid and mycelia were concentrated using rotary evaporation to obtain 50 g and 20 g of extracts, respectively.

The EtOAc extract (50 g) of fermentation was chromatographed on a silica gel column with a gradient of petroleum ether-EtOAc-MeOH (10:0:0–0:10:0–0:0:10) to yield 7 fractions (Fr.1–Fr.7). Fr.3 was fractionated to obtain six subfractions (Fr.3.1–Fr.3.6) using the silica gel chromatography column with a gradient of petroleum ether-EtOAc (10:0–0:10). Compounds **1** (16 mg), **3** (8 mg), **4** (6 mg), and **16** (13 mg) were purified from Fr.3.2 by performing preparative HPLC with MeOH-H_2_O (68:32, *v*/*v*). Fr.3.3 was chromatographed on Sephadex LH-20 (MeOH) to obtain 10 subfractions (Fr.3.3.1–Fr.3.3.10), and Fr.3.3.8 was further purified by performing preparative HPLC with MeOH- H_2_O (70:30, *v*/*v*) to yield **9** (10 mg). Compounds **2** (6 mg) and **12** (2 mg) were separated from Fr.3.4 by performing preparative reverse-phase HPLC with MeOH-H_2_O (68:32, *v*/*v*), and compound **5** (6 mg) was obtained from s Fr.3.6 by performing preparative HPLC with MeOH-H_2_O (72:28, *v/v*).

Subsequently, Fr.4 was fractionated into seven subfractions (Fr.4.1–Fr.4.7) on Sephadex LH-20 (MeOH). Fr.4.1 was repeatedly fractionated by performing HPLC with MeOH-H_2_O (76:24, *v*/*v*) to afford compound **15** (4 mg). Compounds **14** (10 mg) and **8** (3 mg) were isolated from Fr.4.2 and Fr.4.3, respectively, by performing HPLC with MeOH-H_2_O (70:30, *v/v*). Fr.4.4 was further purified by performing preparative HPLC to yield compound **17** (5 mg) with MeOH-H_2_O (58:42, *v*/*v*). Compounds **11** (3 mg) and **20** (2 mg) were isolated from Fr.4.5 using a preparative HPLC column with MeOH-H_2_O (58:42, *v*/*v*). Fr.5 was purified into six subfractions (Fr.5.1–Fr.5.6) using a silica gel column with a step gradient elution of petroleum ether-EtOAc (10:0–0:10). Compounds **10** (3 mg) and **3** (8 mg) were obtained from Fr.5.3 (MeOH: H_2_O, 60:40, *v*/*v*) and Fr.5.5 (MeOH: H_2_O, 72:28, *v*/*v*) using reverse-phase HPLC, respectively. Fr.6 was separated into six subfractions (Fr.6.1–Fr.6.6) using Sephadex LH-20 (MeOH). Compound **18** (8 mg) was separately purified by preparative HPLC (MeOH: H_2_O, 76:24, *v*/*v*) from Fr.6.1. Fr.6.4 was purified by reverse-phase HPLC with an eluent of MeOH-H_2_O (64:36, *v/v*) to yield compound **13** (5 mg)**.**

The MeOH extract (20 g) from the mycelia was chromatographed on a silica gel column with a stepwise gradient of petroleum ether-EtOAc (100:0–0:100) and EtOAc-MeOH (100:0–0:100) to obtain 5 fractions (Fr.1–Fr.5). Fr.2 was treated with Sephadex LH-20 (MeOH) and HPLC successively to obtain compounds **6** (10 mg; MeOH: H_2_O, 72:28, *v*/*v*) and **7** (3 mg; MeOH: H_2_O, 70:30, *v*/*v*). Fr.3 was separated using a silica gel column with step gradients of petroleum ether-EtOAc (10:0–0:10) and EtOAc-MeOH (10:0–0:10) to afford 6 subfractions (Fr.3.1–Fr.3.6). Moreover, compound **19** (2 mg) was purified from Fr.3.4 using Sephadex LH-20 (MeOH).

Fumianthrogliotoxin (**1**): pale green solid; [α${]}_{D}^{25}$ + 118.15 (*c* 0.2, MeOH); UV (MeOH) λmax (log ε) 213 (4.77) nm; IR υ_max_ 3377, 2975, 2924, 1642, 1422, 1386, 1260, 1231, 1193, 1085, 1050, 960, 880 cm^−1^; for ^1^H and ^13^C NMR data, see Table 1; HR-ESI-MS *m*/*z* 645.1813 [M + Na]^+^ (calcd for C_31_H_34_N_4_O_6_S_2_Na^+^, 645.1812).

*N*3-(methyl propionate) indoquizoline (**2**): pale green particulate; UV (MeOH) λmax (log ε) 310 (3.56), 278 (3.59), 217 (4.15) nm; IR υ_max_ 3360, 2975, 1731, 1672, 1558, 1439, 1375, 1242, 1163, 1089, 1050, 881 cm^−1^; for ^1^H and ^13^C NMR data, see Table 2; HR-ESI-MS *m*/*z* 346.1196 [M − H]^−^ (calcd for C_20_H_16_N_3_O_3_^−^, 346.1197).

Anthroxyindole (**3**): light-brown solid; UV (MeOH) λmax (log ε) 334 (3.84), 248 (3.77), 211 (4.21) nm; IR υ_max_ 3348, 1620, 1517, 1431, 1375, 1208, 1050 cm^−1^; for ^1^H NMR data, see Table 3, and for ^13^C NMR data, see Table 4; HR-ESI-MS *m/z* 303.0738 [M + Na]^+^ (calcd for C_16_H_12_N_2_O_3_Na^+^, 303.0740).

(±)-Asperfumiindole A (**4**): dark-brown solid; [α${]}_{D}^{25}$+ 0.197 (*c* 0.2, MeOH); UV (MeOH) λmax (log ε) 338 (3.06), 257 (3.67), 220 (4.25) nm; IR υ_max_ 3366, 2975, 1724, 1677, 1624, 1584, 1563, 1497, 1457, 1434, 1377, 1339, 1299, 1233, 1193, 1161, 1126, 1091, 1047, 1022, 998, 879, 824 cm^−1^; for ^1^H NMR data, see Table 3, and for ^13^C NMR data, see Table 4; HR-ESI-MS *m/z* 347.1002 [M + Na]^+^ (calcd for C_18_H_12_N_2_O_4_Na^+^, 347.1002).

(±)-Asperfumiindole B (**5**): dark-brown solid; [α${]}_{D}^{25}$+ 0.495 (*c* 0.17, MeOH); UV (MeOH) λmax (log ε) 328 (4.08), 256 (4.67), 220 (5.22) nm; IR *υ*_max_ 3359, 2974, 2924, 2256, 1724, 1621, 1574, 1536, 1457, 1432, 1375, 1339, 1243, 1199, 1161, 1126, 1091, 1048, 1024, 1008, 930, 880, 824 cm^−1^; for ^1^H NMR data, see Table 3, and for ^13^C NMR data, see Table 4; HR-ESI-MS *m/z* 347.1002 [M + Na]^+^ (calcd for C_18_H_12_N_2_O_4_Na^+^, 347.1002).

### 3.4. Anti-Pulmonary Fibrosis Activity Analysis

#### 3.4.1. Cell Culture and Treatment

The cell lines used in the manuscript were obtained from Wuhan Pricella Biotechnology Co., Ltd. A549 cells were cultured in RPMI-1640 medium containing 10% FBS, 100 U/mL penicillin, and 100 mg/L streptomycin at 37 °C and 5% CO_2_. The cells were pre-treated with the compound at a concentration of 50 μM for 2 h, followed by treatment with bleomycin at a final concentration of 0.02 U/mL for an additional 48 h.

#### 3.4.2. Quantitative Real-Time PCR (qRT-PCR) Assay

For the mRNA-level analysis of the cultured cells, total RNA was extracted by using the FastPure Cell/Tissue Total RNA Isolation Kit V2 (Nanjing Vazyme Biotechnology Co., Ltd., Nanjing, China). Complementary DNA (cDNA) was synthesized by using HiScript II qRT SuperMix (Nanjing Vazyme Biotechnology Co., Ltd., Nanjing, China). qRT-PCR was performed by using a CFX Connect™ Real-Time PCR Detection System (Bio-Rad Laboratories, Inc., Hercules, CA, USA) and ChamQ Universal SYBR qPCR Master Mix (QIAGEN, Hilden, Germany) with cDNA as the template. Gene expression fold changes were calculated by using the 2^TCM^ method. The primer pairs used in this study are listed in Table 5.

## 4. Conclusions

In summary, a total of **21** alkaloid derivatives were isolated from the fermentation of the deep-sea fungus *Aspergillus fumigatus* AF1, including five undescribed indole alkaloid derivatives, fumianthrogliotoxin (**1**), *N*3-(methyl propionate) indoquizoline (**2**), anthroxyindole (**3**), (±)- asperfumiindole A (**4**), and (±)- asperfumiindole B (**5**). Compounds **2**–**11** and **13**–**21** were evaluated for their anti-pulmonary fibrosis activity in A549 cells. Among all the tested samples, compounds **8** and **13** exhibited better performance, as they significantly downregulated the mRNA expression levels of all three molecular markers (COL1A1, α-SMA, and FN1). This study provides evidence that the employed to amino acid-directed strategy can improve the structural diversity of indole alkaloids in deep-sea-derived fungi. Moreover, indole alkaloids **8** and **13** demonstrated significant downregulation of mRNA expression levels of pulmonary fibrosis markers, highlighting the potential of indole alkaloids as lead compounds in the treatment of pulmonary fibrosis. With the ultimate aim of finding more bioactive indole alkaloids effective against pulmonary fibrosis, further exploration of the amino acid-directed strategy is essential.

## Figures and Tables

**Table 1:** ^1^H (500 MHz) and ^13^C (125 MHz) NMR data of **1** in DMSO-*d*_6_ (*δ* in ppm; *J* in Hz).

Position	*δ*_C_, Type	*δ*_H_, Mult (*J* in Hz)
1	165.2, C	
2	N	
3	72.8, C	
4	166.3, C	
5	N	
6	69.0, CH	4.82, d (14.0)
7	73.7, CH	4.72, d (14.0)
7-NH		5.47, m
8	130.6, CH	5.64, d (9.5)
9	123.5, CH	5.90, m
10	119.3, CH	6.00, m
11	133.1, C	
12	38.4, CH_2_	2.85, m; 3.12, m
13	71.52, C	
14	14.7, CH_3_	2.19, s
15	28.3, CH_3_	2.99, s
16	12.8, CH_3_	2.20, s
17	63.0, CH_2_	3.73, dd (11.0, 3.0); 4.06, d (11.0)
17-OH		5.47, d (3.0)
18	129.3, CH	7.31, d (7.0)
19	128.2, CH	7.25, dd (7.5, 7.0)
20	126.3, CH	7.20, dd (7.5, 7.5)
21	128.2, CH	7.25, dd (7.5, 7.0)
22	129.3, CH	7.31, d (7.0)
23	137.9, C	
24	33.3, CH_2_	2.81, m; 3.12, m
25	53.9, CH	3.89, dt (11.0, 6.5)
26	NH	8.51, brd (6.5)
27	167.7, C	
28	126.3, C	
29	130.3, CH	7.66, dd (7.5, 1.5)
30	124.0, CH	7.20, dd (7.5, 7.5)
31	132.2, CH	7.50, ddd (8.0, 7.5, 1.5)
32	121.0, CH	7.10, d (8.0)
33	136.8, C	
34	171.3, C	
34-OH		10.41, brs

**Table 2:** ^1^H (400 MHz) and ^13^C (100 MHz) NMR data for **2** in CDCl_3_ (*δ* in ppm; *J* in Hz).

Position	*δ*_C_, Type	*δ*_H_, Mult (*J* in Hz)
1	162.8, C	
2	N	
3	151.9, C	
4	N	
5	147.7, C	
6	127.0, CH	7.77, m
7	134.7, C	7.77, m
8	127.3, C	7.50, m
9	126.9, C	8.34, d (8.0)
10	120.6, C	
11	42.0, CH_2_	4.55, t (7.6)
12	33.2, C	2.71, t (7.6)
13	171.4, C	
14	51.9, OCH_3_	3.54, s
15	119.9, CH	7.66, d (8.0)
16	119.9, C	7.22, dd (8.0, 7.5)
17	121.7, C	7.26, dd (8.0, 7.5)
18	123.5, C	7.39, d (8.0)
19	135.8, C	
20	NH	9.07, s
21	125.8, C	7.52, s
22	111.2, C	
23	126.2, C	

**Table 3:** ^1^H NMR (500 MHz) data for compounds **3**–**5** in DMSO-*d*_6_ (*δ* in ppm; *J* in Hz).

Position	3	4	5
	*δ*_H_, Mult (*J* in Hz)		
1	11.94, s	11.02, s	10.99, s
2	7.86, s	7.18, s	7.14, dd (2.0)
3			
4a			
4	8.15, d (7.0)	7.35, d (8.0)	7.33, dd (8.0, 7.0)
5	7.17, dd (7.5, 7.0)	6.93, dd (8.0, 7.0)	6.91, dd (8.0, 7.0)
6	7.21, dd (7.5, 7.0)	7.06, dd (8.0, 7.0)	7.04, dd (8.0, 7.0)
7	7.50, d (7.5)	7.35, d (8.0)	7.33, dd (8.0, 7.0)
7a			
8		5.12, s	5.01, s
9			
10	8.36, s	7.71, s	7.73, d (2.0)
11			
12			6.96, dd (8.0, 2.0)
13	6.71, d (7.5)	6.66, d (8.0)	6.44, d (8.0)
14	7.63, d (7.5)	7.23, d (8.0)	
15			
16			
17		3.66, s	3.62, s

**Table 4:** ^13^C NMR (125 MHz) data for compounds **3**–**5** in DMSO-*d*_6_ (*δ* in ppm).

Position	3	4	5
	*δ*_C_, Type		
1	NH	NH	NH
2	133.2, CH	123.5, CH	123.4, CH
3	115.1, C	112.7, C	113.2, C
4a	126.7, C	126.2, C	126.4, C
4	121.4, CH	118.6, CH	111.6, CH
5	121.1, CH	118.7, CH	118.6, CH
6	122.6, CH	121.3, CH	121.2, CH
7	112.0, CH	111.6, CH	118.7, CH
7a	136.4, C	136.3, C	136.3, C
8	188.2, C	47.1, CH	47.6, CH
9	126.1, C	125.0, C	124.0, C
10	134.0, CH	130.8, CH	131.6, CH
11	126.1, C	110.1, C	120.1, C
12	153.8, C	150.5, C	129.9, CH
13	115.2, CH	116.4, CH	115.2, CH
14	132.2, CH	133.8, CH	149.3, C
15	171.0, C	169.8, C	149.3, C
16	NH	173.4, C	173.7, C
17		57.0, OCH_3_	51.8, CH_3_

**Table 5:** List of primers used in this study.

Primer Name	Primer Sequence (5′-3′)
FN1-F	CGGTGGCTGTCAGTCAAAG
FN1-R	AAACCTCGGCTTCCTCCATAA
α-SMA-F	GTGTTGCCCCTGAAGAGCAT
α-SMA-R	GCTGGGACATTGAAAGTCTCA
COL1A1-F	GTGCGATGACGTGATCTGTGA
COL1A1-R	CGGTGGTTTCTTGGTCGGT
GADPH-F	ACCCAGAAGACTGTGGATGG
GADPH-R	TGCTGTAGCCAAATTCGTTG

## Data Availability

Data are contained within the article or Supplementary Materials.

## Supplementary Materials

The following supporting information can be downloaded at: https://www.mdpi.com/article/10.3390/md23010004/s1, Table S1: Energy analysis and calculated optical rotations of different configurations for **1**; Figures S1–S76: Spectrum of compounds.

## References

[1] Kong F.-D., Zhang S.-L., Zhou S.-Q., Ma Q.-Y., Xie Q.-Y., Chen J.-P., Li J.-H., Zhou L.-M., Yuan J.-Z., Hu Z. (2019). Quinazoline-Containing Indole Alkaloids from the Marine-Derived Fungus *Aspergillus* sp. HNMF114. J. Nat. Prod..

[2] Liao L., You M., Chung B.K., Oh D.-C., Oh K.-B., Shin J. (2015). Alkaloidal Metabolites from a Marine-Derived *Aspergillus* sp. Fungus. J. Nat. Prod..

[3] Zain Ul Arifeen M., Ma Y.-N., Xue Y.-R., Liu C.-H. (2019). Deep-Sea Fungi Could Be the New Arsenal for Bioactive Molecules. Mar. Drugs.

[4] Sun C., Mudassir S., Zhang Z., Feng Y., Chang Y., Che Q., Gu Q., Zhu T., Zhang G., Li D. (2020). Secondary Metabolites from Deep-Sea Derived Microorganisms. Curr. Med. Chem..

[5] Wang W., Yu Y., Keller N.P., Wang P. (2021). Presence, Mode of Action, and Application of Pathway Specific Transcription Factors in *Aspergillus* Biosynthetic Gene Clusters. Int. J. Mol. Sci..

[6] Youssef F.S., Simal-Gandara J. (2021). Comprehensive Overview on the Chemistry and Biological Activities of Selected Alkaloid Producing Marine-Derived Fungi as a Valuable Reservoir of Drug Entities. Biomedicines.

[7] Zhu J., Song L., Shen S., Fu W., Zhu Y., Liu L. (2023). Bioactive Alkaloids as Secondary Metabolites from Plant Endophytic *Aspergillus* Genus. Molecules.

[8] Li S.-G., Wang K.-B., Gong C., Bao Y., Qin N.-B., Li D.-H., Li Z.-L., Bai J., Hua H.-M. (2018). Cytotoxic Quinazoline Alkaloids from the Seeds of *Peganum harmala*. Bioorganic Med. Chem. Lett..

[9] Buttachon S., Chandrapatya A., Manoch L., Silva A., Gales L., Bruyère C., Kiss R., Kijjoa A. (2012). Sartorymensin, a New Indole Alkaloid, and New Analogues of Tryptoquivaline and Fiscalins Produced by *Neosartorya siamensis* (KUFC 6349). Tetrahedron.

[10] Long S., Duarte D., Carvalho C., Oliveira R., Santarém N., Palmeira A., Resende D.I.S.P., Silva A.M.S., Moreira R., Kijjoa A. (2022). Indole-Containing Pyrazino[2,1-*b*]Quinazoline-3,6-Diones Active against *Plasmodium* and *Trypanosomatids*. ACS Med. Chem. Lett..

[11] Numata A., Takahashi C., Matsushita T., Miyamoto T., Kawai K., Usami Y., Matsumura E., Inoue M., Ohishi H., Shingu T. (1992). Fumiquinazolines, Novel Metabolites of a Fungus Isolated from a Saltfish. Tetrahedron Lett..

[12] Takahashi C., Matsushita T., Doi M., Minoura K., Shingu T., Kumeda Y., Numata A. (1995). Fumiquinazolines A–G, Novel Metabolites of a Fungus Separated from a *Pseudolabrus* Marine Fish. J. Chem. Soc. Perkin Trans..

[13] Liu R., Li H., Yang J., An Z. (2018). Quinazolinones Isolated from *Aspergillus* sp., an Endophytic Fungus of *Astragalus membranaceus*. Chem. Nat. Compd..

[14] Liu Y., Cui Y., Lu L., Gong Y., Han W., Piao G. (2020). Natural Indole-containing Alkaloids and Their Antibacterial Activities. Arch. Pharm..

[15] Hu Y., Chen S., Yang F., Dong S. (2021). Marine Indole Alkaloids—Isolation, Structure and Bioactivities. Mar. Drugs.

[16] Islam F., Dehbia Z., Zehravi M., Das R., Sivakumar M., Krishnan K., Billah A.A.M., Bose B., Ghosh A., Paul S. (2023). Indole Alkaloids from Marine Resources: Understandings from Therapeutic Point of View to Treat Cancers. Chem. Biol. Interact..

[17] Alrajhi N.N. (2023). Post-COVID-19 Pulmonary Fibrosis: An Ongoing Concern. Ann. Thorac. Med..

[18] Perez-Favila A., Garza-Veloz I. (2024). Antifibrotic Drugs against Idiopathic Pulmonary Fibrosis and Pulmonary Fibrosis Induced by COVID-19: Therapeutic Approaches and Potential Diagnostic Biomarkers. Int. J. Mol. Sci..

[19] Qin R., Zhao Q., Han B., Zhu H.-P., Peng C., Zhan G., Huang W. (2022). Indole-Based Small Molecules as Potential Therapeutic Agents for the Treatment of Fibrosis. Front. Pharmacol..

[20] Cheng Z., Lou L., Liu D., Li X., Proksch P., Yin S., Lin W. (2016). Versiquinazolines A–K, Fumiquinazoline-Type Alkaloids from the Gorgonian-Derived Fungus *Aspergillus versicolor* LZD-14-1. J. Nat. Prod..

[21] Guo Y.-W., Liu X.-J., Yuan J., Li H.-J., Mahmud T., Hong M.-J., Yu J.-C., Lan W.-J. (2020). l-Tryptophan Induces a Marine-Derived *Fusarium* sp. to Produce Indole Alkaloids with Activity against the Zika Virus. J. Nat. Prod..

[22] Huang L.-H., Xu M.-Y., Li H.-J., Li J.-Q., Chen Y.-X., Ma W.-Z., Li Y.-P., Xu J., Yang D.-P., Lan W.-J. (2017). Amino Acid-Directed Strategy for Inducing the Marine-Derived Fungus *Scedosporium apiospermum* F41–1 to Maximize Alkaloid Diversity. Org. Lett..

[23] Kirby G.W., Robins D.J., Sefton M.A., Talekar R.R. (1980). Biosynthesis of Bisdethiobis(Methylthio)Gliotoxin, a New Metabolite of *Gliocladium deliquescens*. J. Chem. Soc. Perkin Trans..

[24] Li X., Kim S.-K., Nam K.W., Kang J.S., Choi H.D., Son B.W. (2006). A New Antibacterial Dioxopiperazine Alkaloid Related to Gliotoxin from a Marine Isolate of the Fungus *Pseudallescheria*. J. Antibiot..

[25] Afiyatullov S.S., Kalinovskii A.I., Pivkin M.V., Dmitrenok P.S., Kuznetsova T.A. (2005). Alkaloids from the Marine Isolate of the Fungus *Aspergillus fumigatus*. Chem. Nat. Compd..

[26] Barrow C.J., Sun H.H. (1994). Spiroquinazoline, a Novel Substance P Inhibitor with a New Carbon Skeleton, Isolated from *Aspergillus flavipes*. J. Nat. Prod..

[27] Zhang W., Li J., Wei C., Deng X., Xu J. (2022). Chemical Epigenetic Modifiers Enhance the Production of Immunosuppressants from the Endophytic Fungus *Aspergillus fumigatus* Isolated from *Cynodon dactylon*. Nat. Prod. Res..

[28] Larsen T.O., Frydenvang K., Frisvad J.C., Christophersen C. (1998). UV-Guided Isolation of Alantrypinone, a Novel *Penicillium* Alkaloid. J. Nat. Prod..

[29] Limbadri S., Luo X., Lin X., Liao S., Wang J., Zhou X., Yang B., Liu Y. (2018). Bioactive Novel Indole Alkaloids and Steroids from Deep Sea-Derived Fungus *Aspergillus fumigatus* SCSIO 41012. Molecules.

[30] Jiao R.H., Xu S., Liu J.Y., Ge H.M., Ding H., Xu C., Zhu H.L., Tan R.X. (2006). Chaetominine, a Cytotoxic Alkaloid Produced by Endophytic *Chaetomium* sp. IFB-E015. Org. Lett..

[31] Sun J.-H., Yang Z.-D., Zhang Y.-F. (2019). Chemical Constituents and Bioactivity of a Fungal Endophyte from *Lamium amplexicaule*. Chem. Nat. Compd..

[32] Afiyatullov S.S., Kalinovskii A.I., Pivkin M.V., Dmitrenok P.S., Kuznetsova T.A. (2004). Fumitremorgins from the Marine Isolate of the Fungus *Aspergillus fumigatus*. Chem. Nat. Compd..

[33] Çetinel Aksoy S., Küçüksolak M., Uze A., Bedir E. (2021). Benzodiazepine Derivatives from Marine-Derived *Streptomyces cacaoi* 14CM034. Rec. Nat. Prod..

[34] Peng Q., Cai J., Long J., Yang B., Lin X., Wang J., Xiao J., Liu Y., Zhou X. (2021). New Azaphthalide and Phthalide Derivatives from the Marine Coral-Derived Fungus *Aspergillus* sp. SCSIO41405. Phytochem. Lett..

[35] Cha X.-J., Pu G., Xiong R.-F., Ma Y.-Y., Zhang G.-H., Yao H., Kong G.-H., Bao M.-F., Hu Q.-F., Li Y.-K. (2023). Carboline Alkaloids from the Cigar Tobacco-Derived Fungi *Aspergillus* sp. and Their Anti-TMV Activity. Chem. Nat. Compd..

[36] Kwon H.C., Lee B.G., Kim S.H., Jung C.M., Hong S.Y., Han J.W., Lee H.W., Zee O.P., Lee K.R. (1999). Inducible Nitric Oxide Synthase Inhibitors from *Melia azedarach* var. Japonica. Arch. Pharm. Res..

[37] Wang J., Jiang M., Xiong A., Zhang L., Luo L., Liu Y., Liu S., Ran Q., Wu D., Xiong Y. (2022). Integrated Analysis of Single-Cell and Bulk RNA Sequencing Reveals pro-Fibrotic PLA2G7 High Macrophages in Pulmonary Fibrosis. Pharmacol. Res..

[38] Al-Habeeb F., Aloufi N., Traboulsi H., Liu X., Nair P., Haston C., Azuelos I., Huang S.K., White E.S., Gallouzi I.E. (2021). Human Antigen R Promotes Lung Fibroblast Differentiation to Myofibroblasts and Increases Extracellular Matrix Production. J. Cell Physiol..

[39] Cárdenes N., Sembrat J., Noda K., Lovelace T., Álvarez D., Bittar H.E.T., Philips B.J., Nouraie M., Benos P.V., Sánchez P.G. (2021). Human Ex Vivo Lung Perfusion: A Novel Model to Study Human Lung Diseases. Sci. Rep..

