# Supplementary material for: Five New Indole Alkaloid Derivatives from Deep-Sea Fungus Aspergillus fumigatus AF1
Source: Mar Drugs. 2024 Dec 25;23(1):4. doi: 10.3390/md23010004 (PMC11766945; doi:10.3390/md23010004)
Supplement: Supplementary file 1 [file marinedrugs-23-00004-s001.zip › marinedrugs-3386941-supplementary.pdf]

Supplementary Materials

Five New Indole Alkaloid Derivatives from Deep-Sea Fungus *Aspergillus fumigatus* AF1

Lai-Hui Dai [1,†], Gao-Rong Zhang [1,4 †], Yang-Hui Ou [2,†], Xiao-Jing Liu [1], Hong-Liang Yao [2], Wen-Hao Hu [1], Hou-Jin Li [3,*] and Wen-Jian Lan [1,*]

1 School of Pharmaceutical Sciences, GBRCE for Functional Molecular Engineering, Sun Yat-Sen University, Guangzhou 510006, China; dailh5@mail2.sysu.edu.cn (L.-H. D.); cpulxj1003@163.com (X.-J.L.); huwh9@mail.sysu.edu.cn (W.-H.W.).

2 Guangdong Key Laboratory of Animal Conservation and Resource Utilization, Guangdong Public Labora-tory of Wild Animal Conservation and Utilization Institute of Zoology, Guangdong Academy of Sciences, Guangzhou, 510260, China; ouyh0807@gmail.com (Y.-H.O.); yaohl@giz.gd.cn (H.-L.Y.)

3 School of Chemistry, Sun Yat-sen University, Guangzhou 510006, China;

4 Guangxi Collaborative Innovation Center of Modern Sericulture and Silk, Hechi University, Hechi 546300, China; zhanggr8@mail2.sysu.edu.cn (G.-R.Z.).

† These authors contributed equally to this work and should be considered co-first authors.

* Correspondence: lanwj@mail.sysu.edu.cn (W.-J.L.); ceslhj@mail.sysu.edu.cn (H.-J.L.); Tel.: +86-20-39943042.

The contents of Table

Table S1. Energy analysis and calculated optical rotations of different configurations for **1**. 5

The contents of Figures

Figure S1. HRESIMS spectrum of compound **1**. 6

Figure S2. ^{1}H NMR spectrum of compound **1** in DMSO-d_6 (500 MHz). 7

Figure S3. ^{13}C NMR spectrum of compound **1** in DMSO-d_6 (125 MHz). 7

Figure S4. DEPT 135 spectrum of compound **1** in DMSO-d_6 (125 MHz). 8

Figure S5. ^{1}H-^{1}H COSY spectrum of compound **1** in DMSO-d_6. 8

Figure S6. HSQC spectrum of compound **1** in DMSO-d_6. 9

Figure S7. HMBC spectrum of compound **1** in DMSO-d_6. 9

Figure S8. NOESY spectrum of compound **1** in DMSO-d_6. 10

Figure S9. UV spectrum of compound **1**. 10

Figure S10. IR spectrum of compound **1**. 11

Figure S11. HRESIMS spectrum of compound **2**. 12

Figure S12. ^{1}H NMR spectrum of compound **2** in $CDCl_3$(400 MHz). 13

Figure S13. ^{13}C NMR spectrum of compound **2** in $CDCl_3$(100 MHz). 13

Figure S14. DEPT 135 spectrum of compound **2** in $CDCl_3$(100 MHz). 14

Figure S15. ^{1}H-^{1}H COSY spectrum of compound **2** in $CDCl_3$. 14

Figure S16. HSQC spectrum of compound **2** in $CDCl_3$. 15

Figure S17. HMBC spectrum of compound **2** in $CDCl_3$. 15

Figure S18. UV spectrum of compound **2**. 16

Figure S19. IR spectrum of compound **2**. 16

Figure S20. HRESIMS spectrum of compound **3**. 17

Figure S21. ^{1}H NMR spectrum of compound **3** in DMSO-d_6 (500 MHz). 18

Figure S22. ^{13}C NMR spectrum of compound **3** in DMSO-d_6(125 MHz). 18

Figure S23. ^{1}H-^{1}H COSY spectrum of compound **3** in DMSO-d_6. 19

Figure S24. HSQC spectrum of compound **3** in DMSO-d_6. 19

Figure S25. HMBC spectrum of compound **3** in DMSO-d_6. 20

Figure S26. UV spectrum of compound **3**. 20
Figure S27. IR spectrum of compound **3**. 21
Figure S28. HRESIMS spectrum of compound **4**. 22
Figure S29. ^{1}H NMR spectrum of compound **4** in DMSO-d_6 (500 MHz). 23
Figure S30. ^{13}C NMR spectrum of compound **4** in DMSO-d_6(125 MHz). 23
Figure S31. DEPT 90 spectrum of compound **4** in DMSO-d_6 (400 MHz). 24
Figure S32. ^{1}H-^{1}H COSY spectrum of compound **4** in DMSO-d_6. 24
Figure S33. HSQC spectrum of compound **4** in DMSO-d_6. 25
Figure S34. HMBC spectrum of compound **4** in DMSO-d_6. 25
Figure S35. UV spectrum of compound **4**. 26
Figure S36. IR spectrum of compound **4**. 26
Figure S37. HRESIMS spectrum of compound **5**. 27
Figure S38. ^{1}H NMR spectrum of compound **5** in DMSO-d_6(500 MHz). 28
Figure S39. ^{13}C NMR spectrum of compound **5** in DMSO-d_6(125MHz). 28
Figure S40. ^{1}H-^{1}H COSY spectrum of compound **5** in DMSO-d_6. 29
Figure S41. HSQC spectrum of compound **5** in DMSO-d_6. 29
Figure S42. HMBC spectrum of compound **5** in DMSO-d_6. 30
Figure S43. UV spectrum of compound **5**. 30
Figure S44. IR spectrum of compound **5**. 31
Figure S45. ^{1}H NMR spectrum of compound **6** in $CDCl_3$ (400 MHz). 31
Figure S46. ^{13}C NMR spectrum of compound **6** in $CDCl_3$ (100 MHz). 32
Figure S47. ^{1}H NMR spectrum of compound **7** in DMSO-d_6 (400 MHz). 32
Figure S48. ^{13}C NMR spectrum of compound **7** in DMSO-d_6 (100 MHz). 33
Figure S49. ^{1}H NMR spectrum of compound **8** in CD_3OD (400 MHz). 33
Figure S50. ^{13}C NMR spectrum of compound **8** in CD_3OD (100 MHz). 34
Figure S51. ^{1}H NMR spectrum of compound **9** in DMSO-d_6 (500 MHz). 34
Figure S52. ^{13}C NMR spectrum of compound **9** in DMSO-d_6 (125 MHz). 35
Figure S53. ^{1}H NMR spectrum of compound **10** in DMSO-d_6 (500 MHz). 35
Figure S54. ^{13}C NMR spectrum of compound **10** in DMSO-d_6 (125 MHz). 36
Figure S55. ^{1}H NMR spectrum of compound **11** in DMSO-d_6 (500 MHz). 36
Figure S56. ^{13}C NMR spectrum of compound **11** in DMSO-d_6 (125 MHz). 37
Figure S57. ^{1}H NMR spectrum of compound **12** in acetone-d_6 (400 MHz). 37

Figure S58. ^{13}C NMR spectrum of compound **12** in acetone-d_6 (100 MHz).............38
Figure S59. ^{1}H NMR spectrum of compound **13** in DMSO-d_6 (400 MHz)................38
Figure S60. ^{13}C NMR spectrum of compound **13** in DMSO-d_6 (100 MHz). 39
Figure S61. ^{1}H NMR spectrum of compound **14** in acetone-d_6 (500 MHz)..............39
Figure S62. ^{13}C NMR spectrum of compound **14** in acetone-d_6 (125 MHz).............40
Figure S63. ^{1}H NMR spectrum of compound **15** in acetone-d_6 (400 MHz)..............40
Figure S64. ^{13}C NMR spectrum of compound **15** in acetone-d_6 (100 MHz).............41
Figure S65. ^{1}H NMR spectrum of compound **16** in acetone-d_6 (400 MHz)..............41
Figure S66. ^{13}C NMR spectrum of compound **16** in acetone-d_6 (125 MHz).............42
Figure S67. ^{1}H NMR spectrum of compound **17** in DMSO-d_6 (400 MHz)................42
Figure S68. ^{13}C NMR spectrum of compound **17** in DMSO-d_6 (100 MHz). 43
Figure S69. ^{1}H NMR spectrum of compound **18** in DMSO-d_6 (500 MHz)................43
Figure S70. ^{13}C NMR spectrum of compound **18** in DMSO-d_6 (125 MHz). 44
Figure S71. ^{1}H NMR spectrum of compound **19** in acetone-d_6 (400 MHz)..............44
Figure S72. ^{13}C NMR spectrum of compound **19** in acetone-d_6 (100 MHz).............45
Figure S73. ^{1}H NMR spectrum of compound **20** in DMSO-d_6 (400 MHz)................45
Figure S74. ^{13}C NMR spectrum of compound **20** in DMSO-d_6 (100 MHz). 46
Figure S75. ^{1}H NMR spectrum of compound **21** in CD_3OD (500 MHz). 46
Figure S76. ^{13}C NMR spectrum of compound **21** in CD_3OD (125 MHz).................47

Table S1. Energy analysis and calculated optical rotations of different configurations for **1**.

Configuration	Conformer	G (Hartree)	△E (kcal/mol)	Population %	OR_{cal}	Weighted average
3*S*, 6*R*, 7*R*, 13*R*, 25*R* **-1**	C1	6184.3304115290	-1.02	56.0	-783.85	-577.239
	C2	6043.7297650266	-0.16	13.0	-553.25	
	C3	6079.4181672144	0.00	9.9	-27.38	
	C4	6180.2793215568	0.24	6.7	-716.69	
	C5	6133.3988439723	0.42	4.9	-243.99	
	C6	6158.2012350164	0.80	2.6	-141.39	
3*R*, 6*S*, 7*S*, 13*R*, 25*S*-**1**	C1	6155.2489977316	0.00	74.3	261.88	+228.195
	C2	6168.1231921109	0.93	15.4	-104.54	
	C3	6156.4622953388	1.42	6.8	731.14	

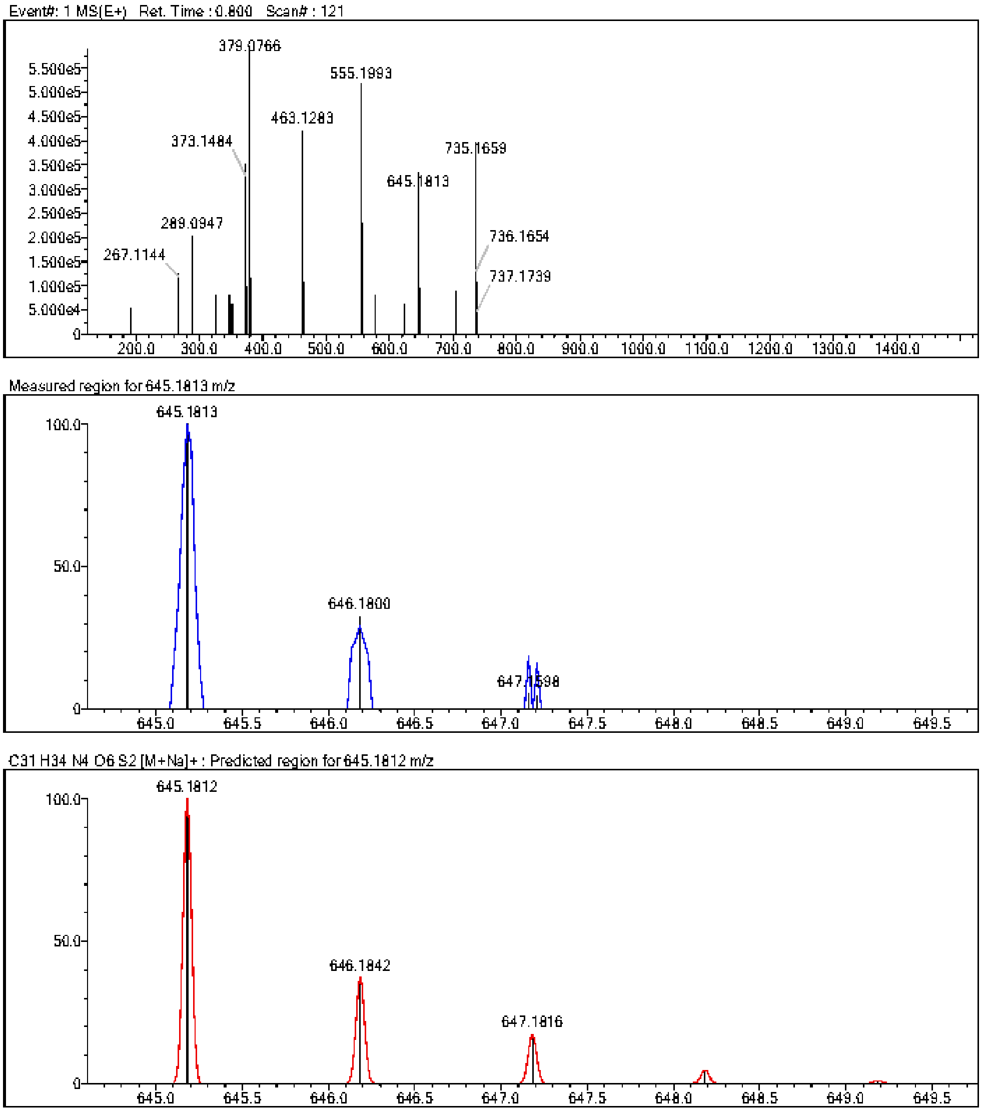

Rank	Score	Formula (M)	Ion	Meas. m/z	Pred. m/z	Df (mDa)	Df (ppm)	Iso	DBE
1	0.00	C31 H34 N4 O6 S2	[M+Na]+	645.1813	645.1812	0.1	0.15	0.00	17.0

Figure S1. HRESIMS spectrum of compound **1**.

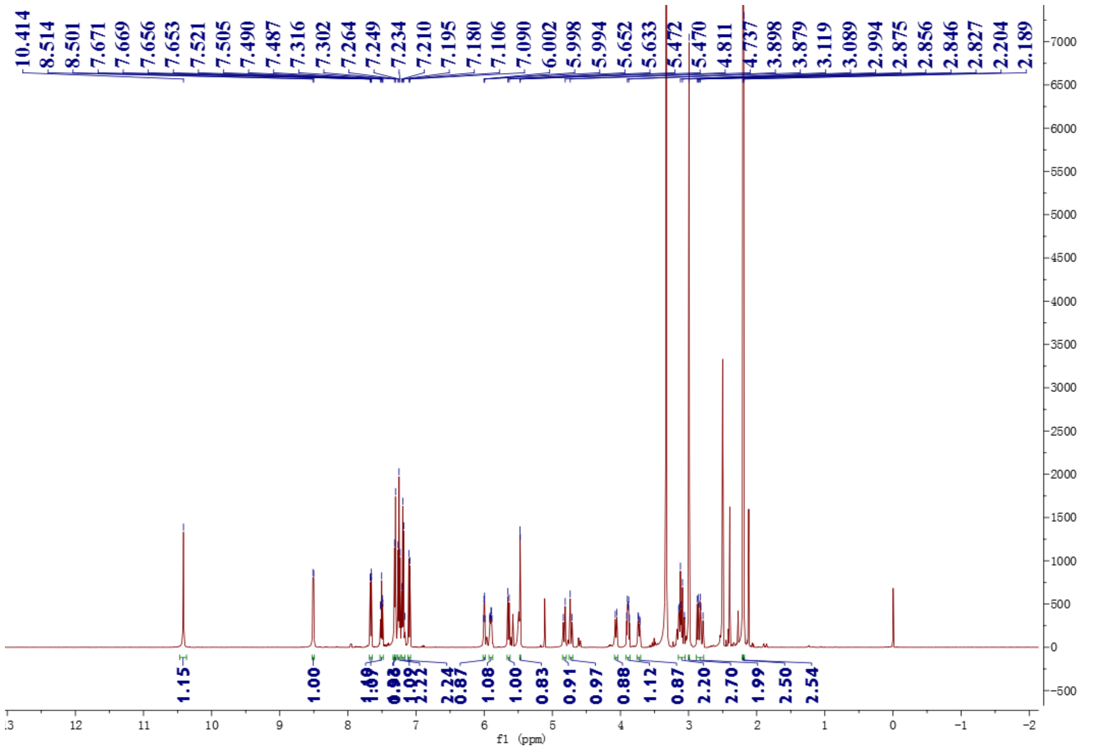

Figure S2. ^{1}H NMR spectrum of compound **1** in DMSO-d_6 (500 MHz).

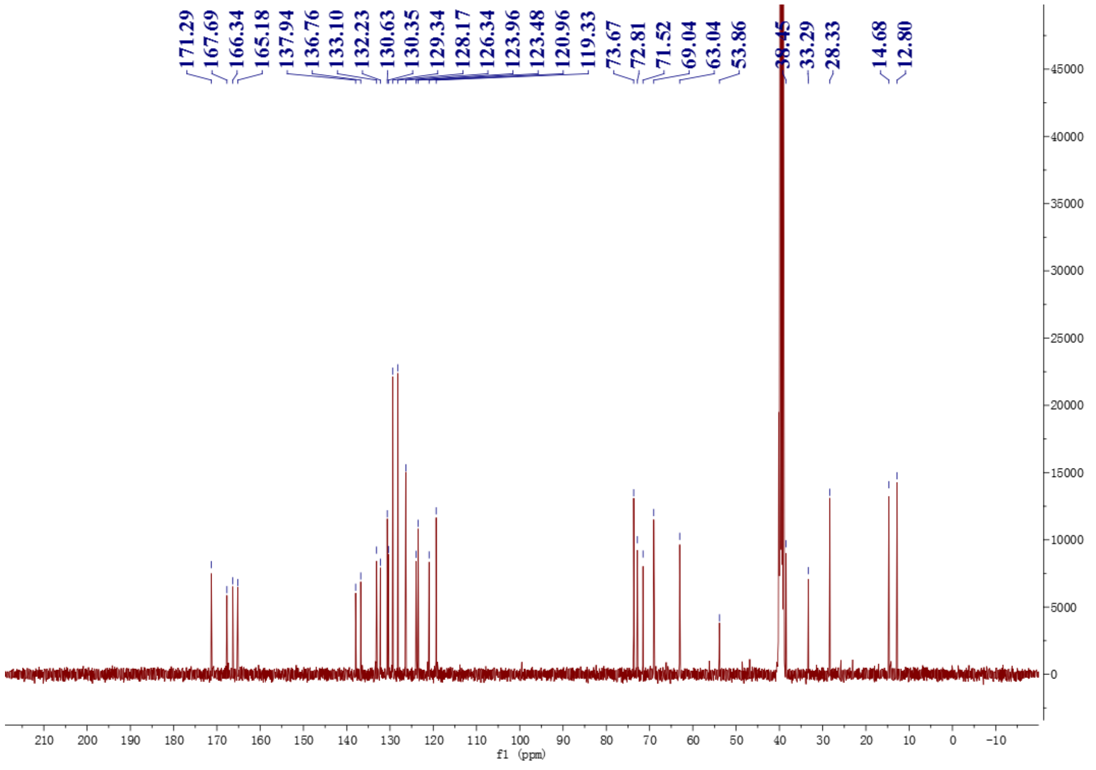

Figure S3. ^{13}C NMR spectrum of compound **1** in DMSO-d_6 (125 MHz).

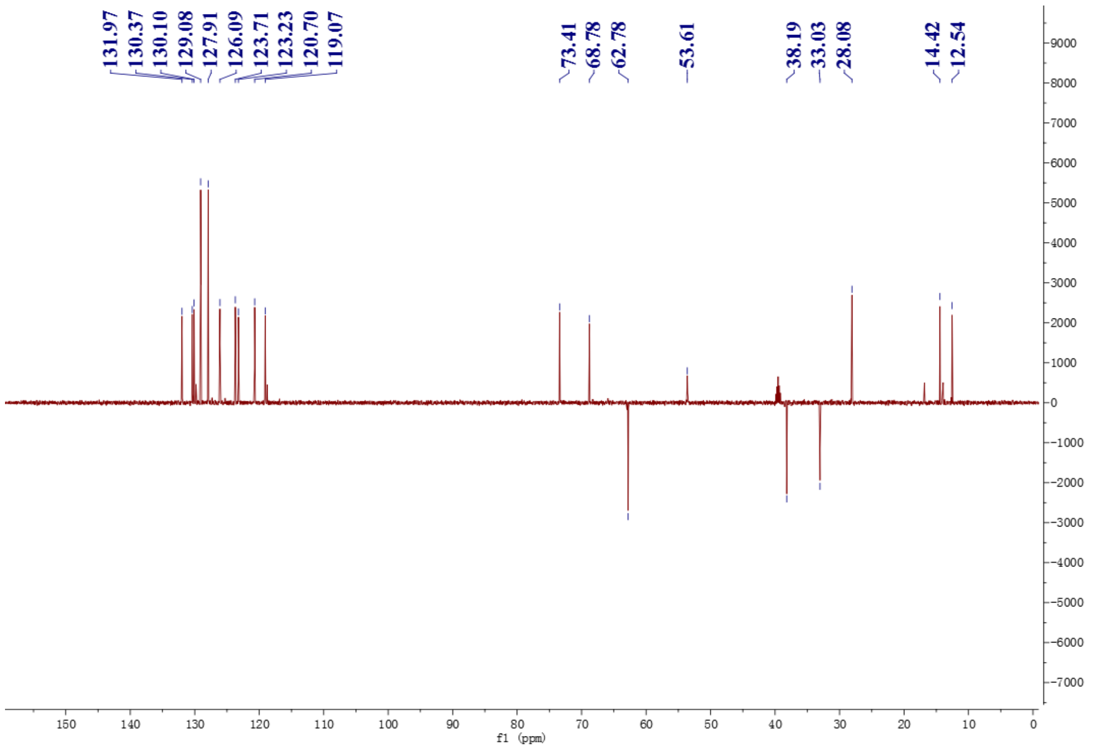

Figure S4. DEPT 135 spectrum of compound **1** in DMSO-d_6 (125 MHz).

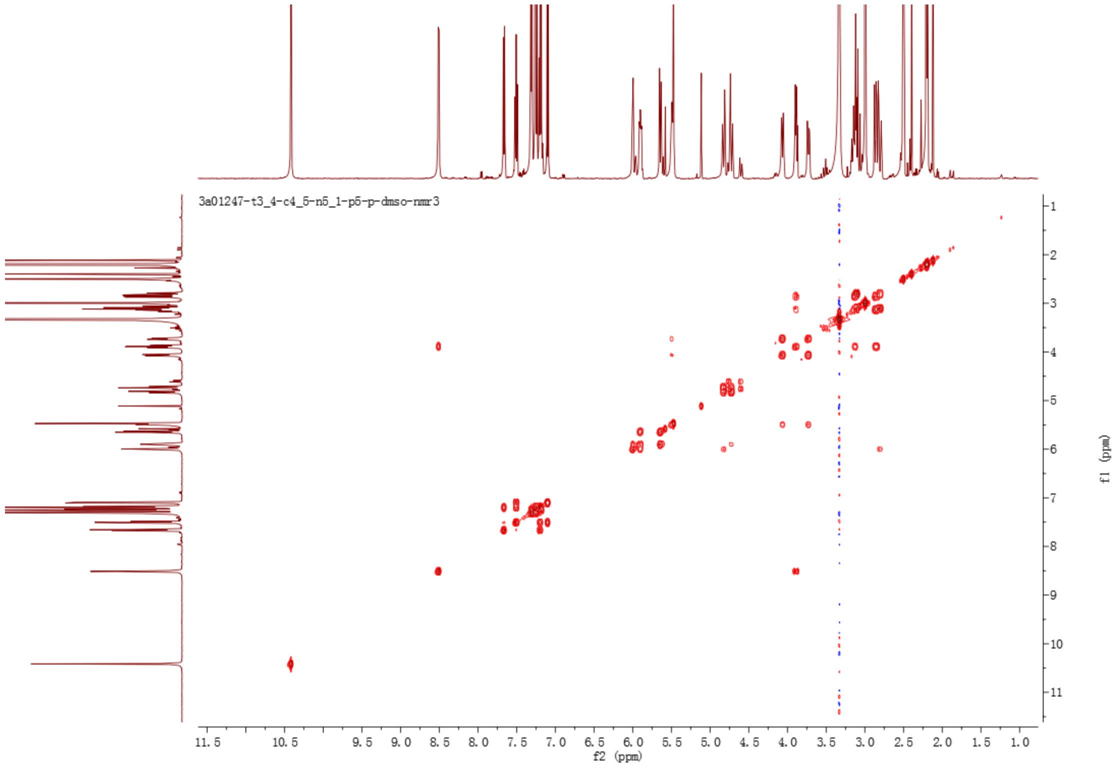

Figure S5. ^{1}H-^{1}H COSY spectrum of compound **1** in DMSO-d_6.

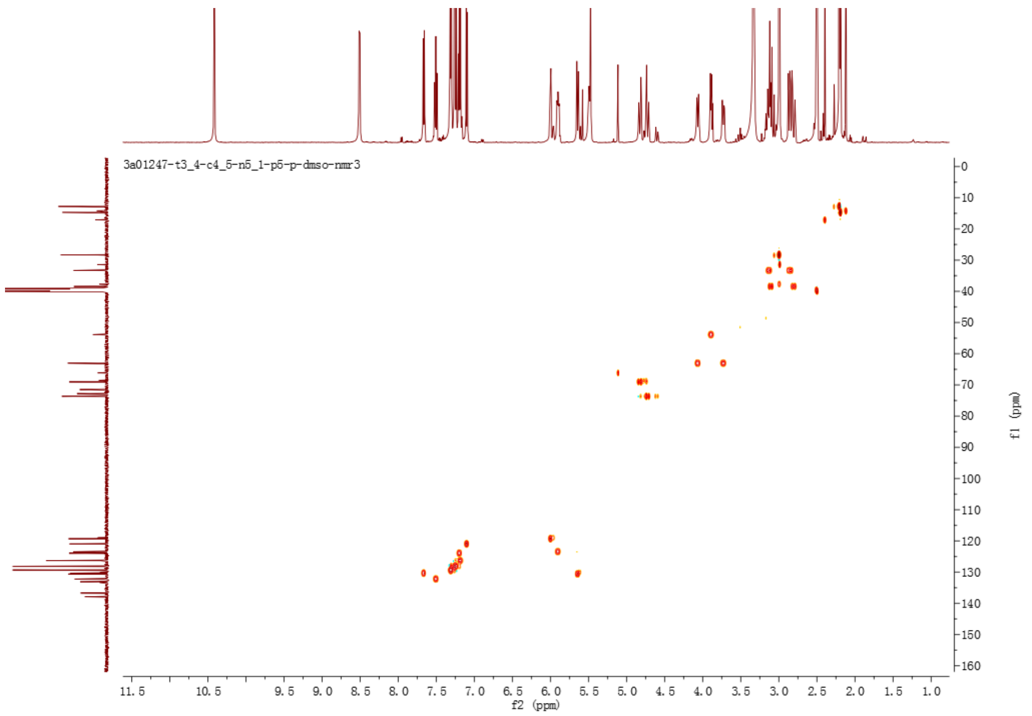

Figure S6. HSQC spectrum of compound **1** in DMSO-d_6.

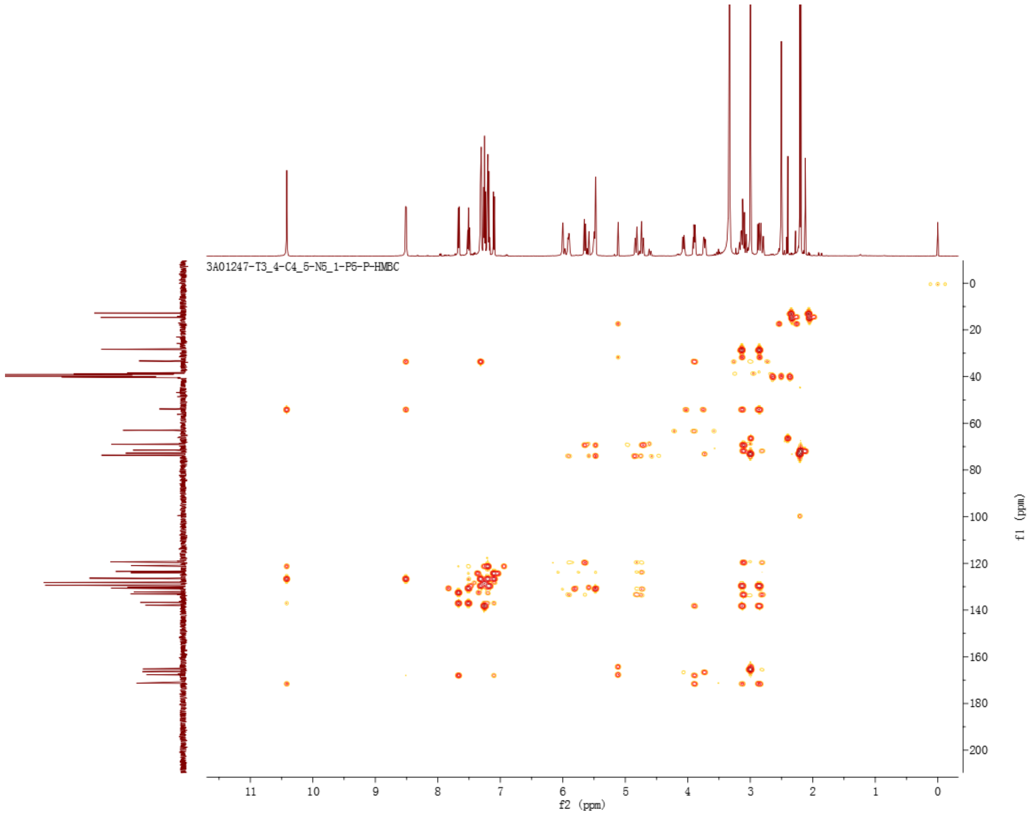

Figure S7. HMBC spectrum of compound **1** in DMSO-d_6.

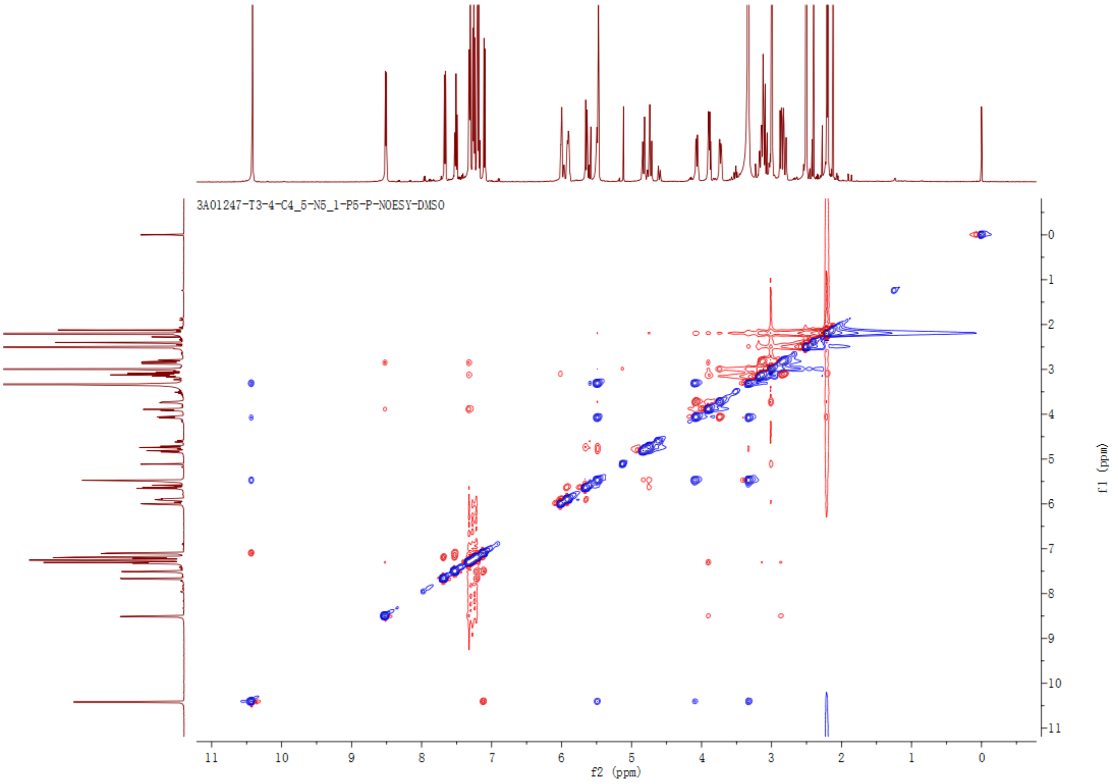

Figure S8. NOESY spectrum of compound **1** in DMSO-d_6.

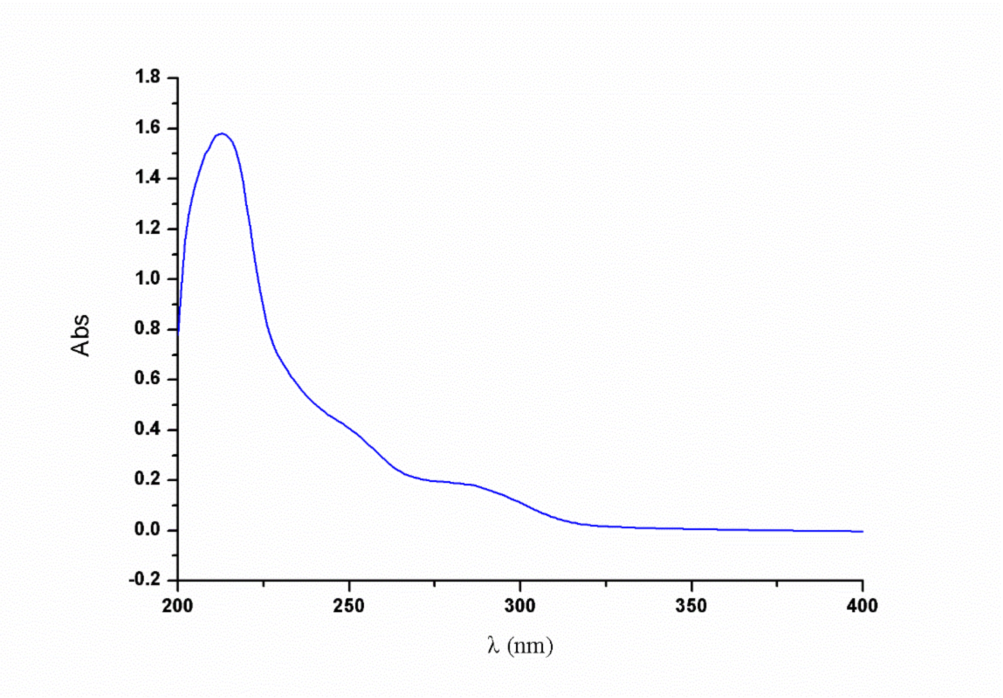

Figure S9. UV spectrum of compound **1**.

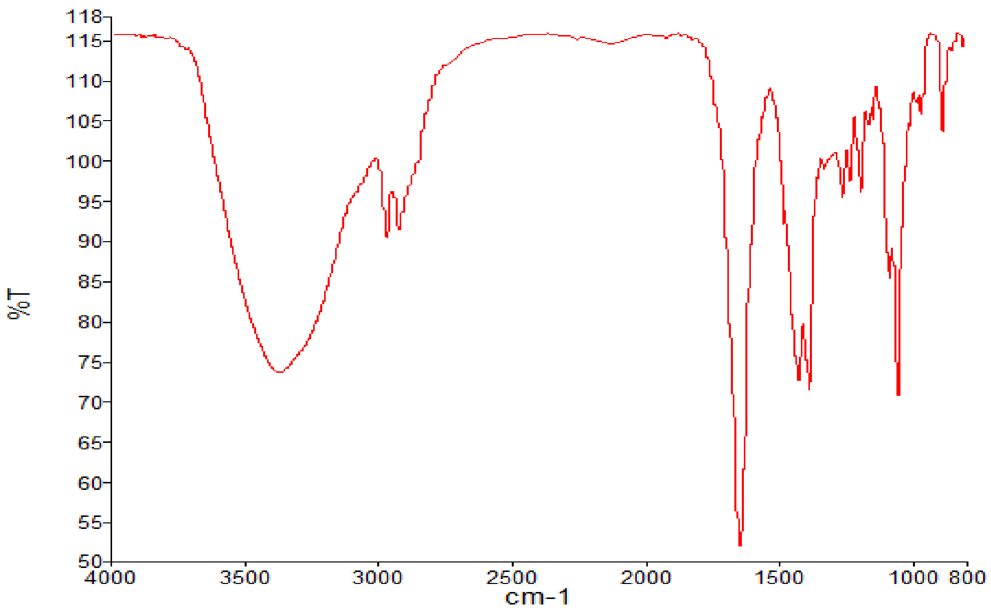

Figure S10. IR spectrum of compound **1**.

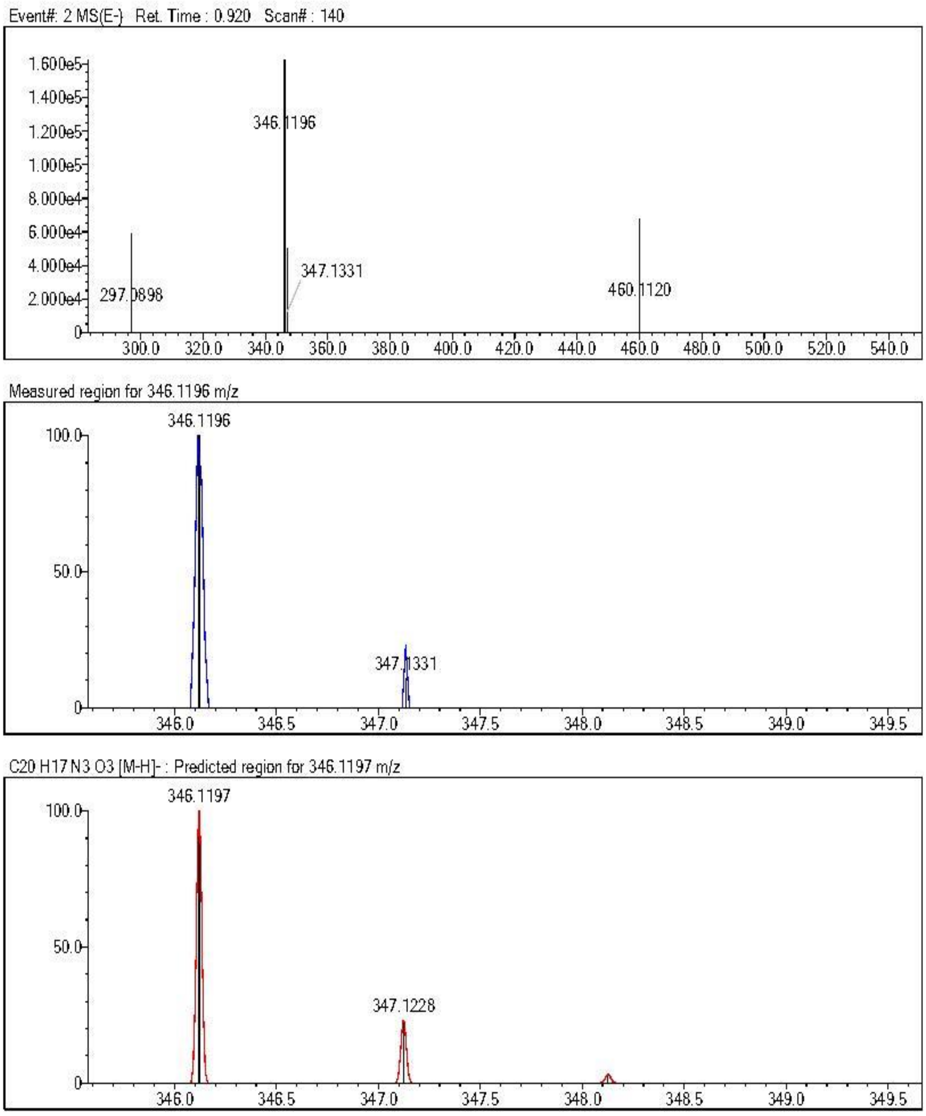

Rank	Score	Formula (M)	Ion	Meas. m/z	Pred. m/z	Df. (mDa)	Df. (ppm)	Iso	DBE
1	26.23	C20 H17 N3 O3	[M-H]-	346.1196	346.1197	-0.1	-0.29	26.23	14.0

Figure S11. HRESIMS spectrum of compound **2**.

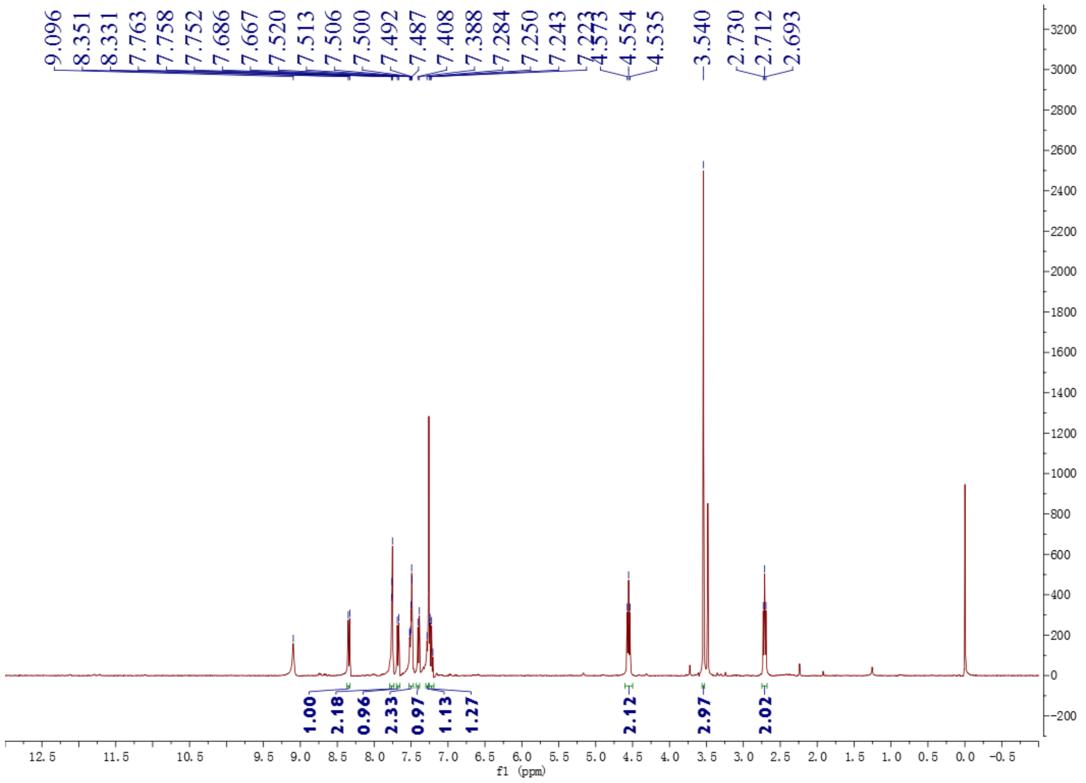

Figure S12. ^{1}H NMR spectrum of compound **2** in $CDCl_3$(400 MHz).

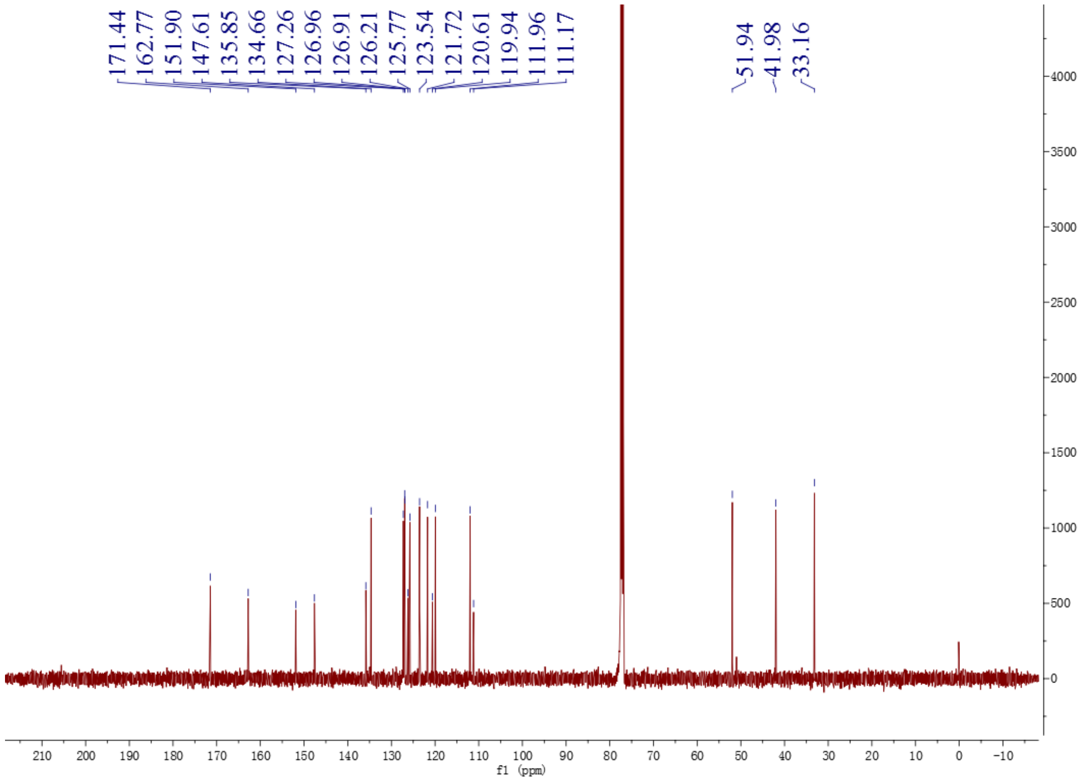

Figure S13. ^{13}C NMR spectrum of compound **2** in $CDCl_3$(100 MHz).

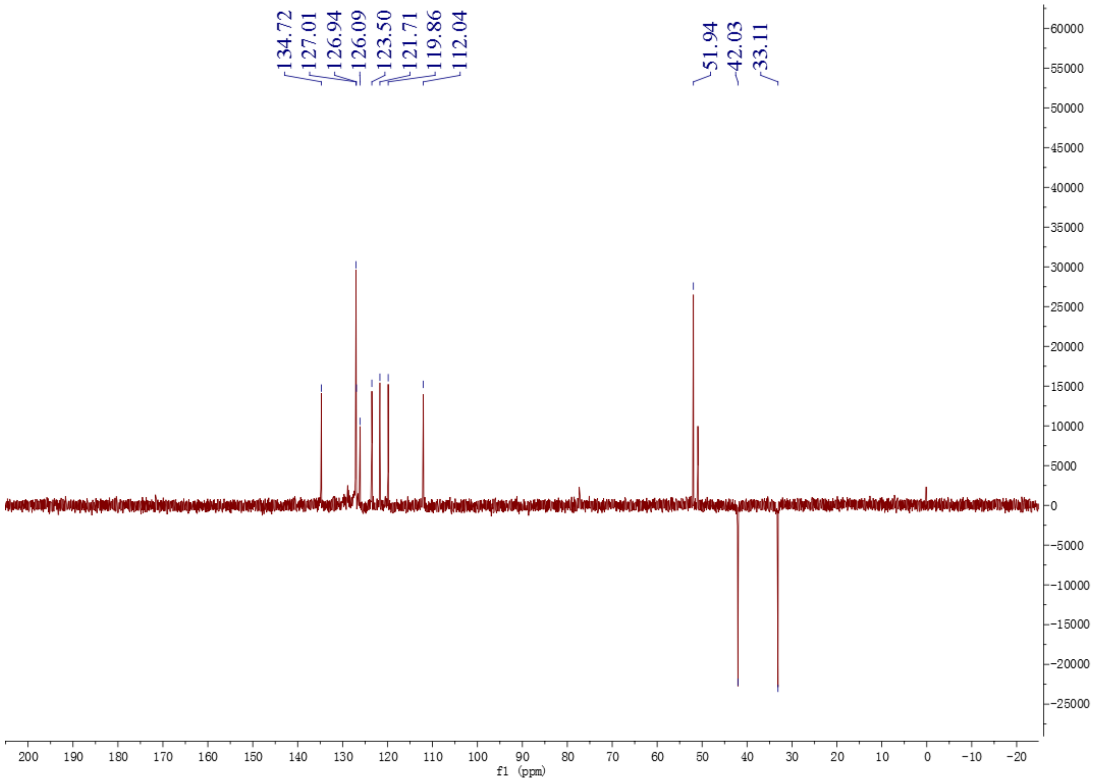

Figure S14. DEPT 135 spectrum of compound **2** in $CDCl_3$(100 MHz).

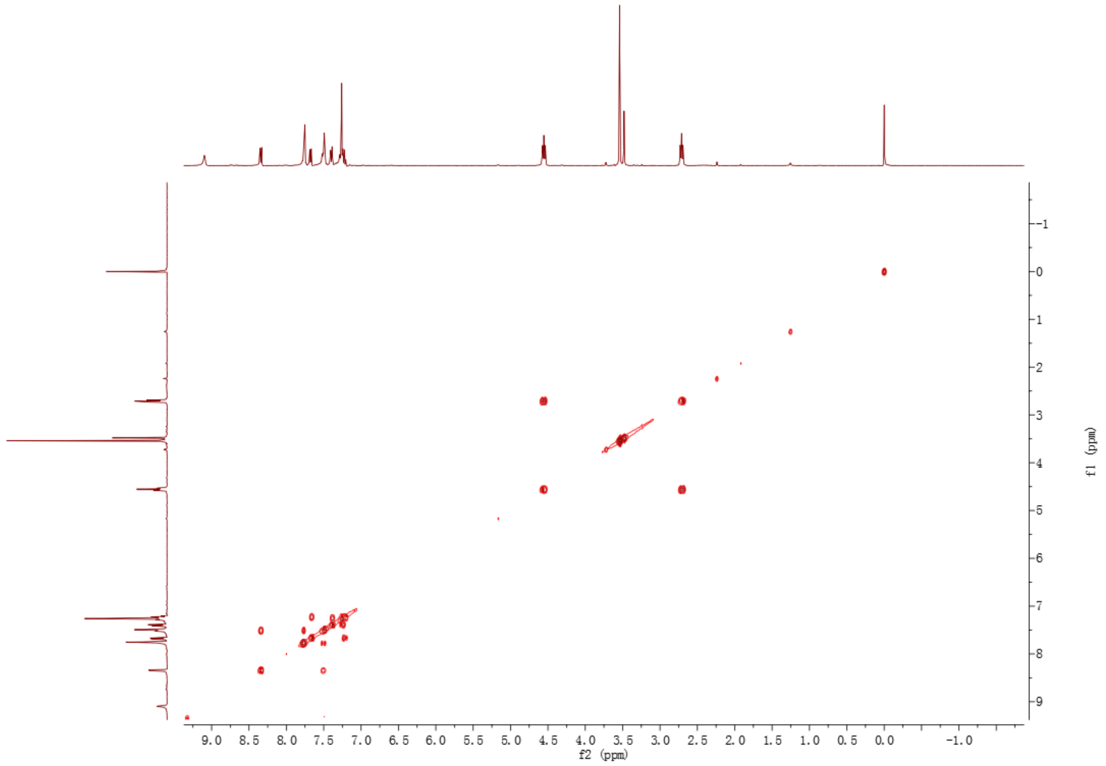

Figure S15. ^1H-^1H COSY spectrum of compound **2** in $CDCl_3$.

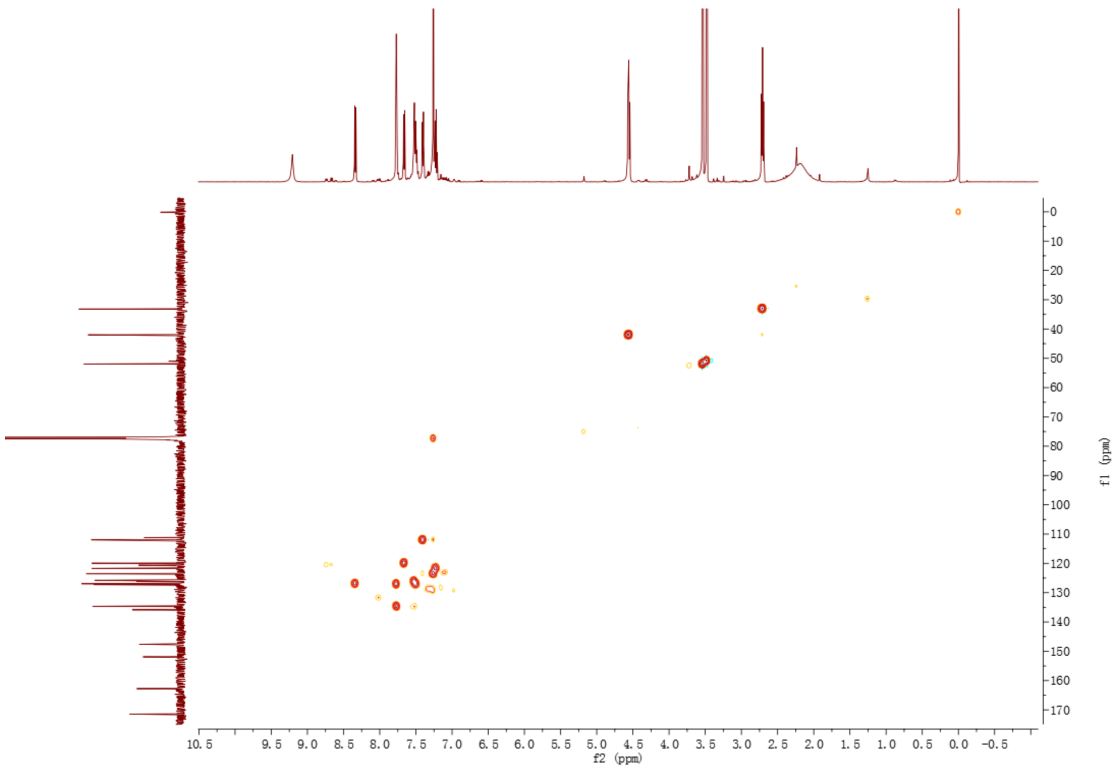

Figure S16. HSQC spectrum of compound **2** in $CDCl_3$.

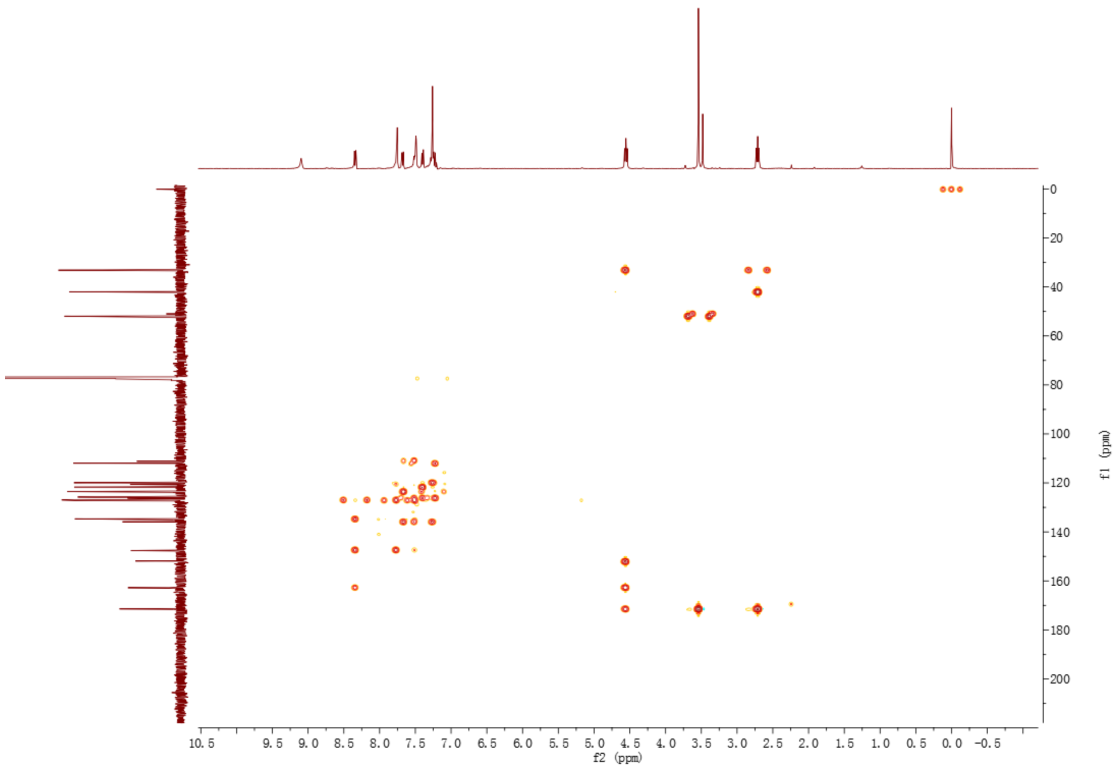

Figure S17. HMBC spectrum of compound **2** in $CDCl_3$.

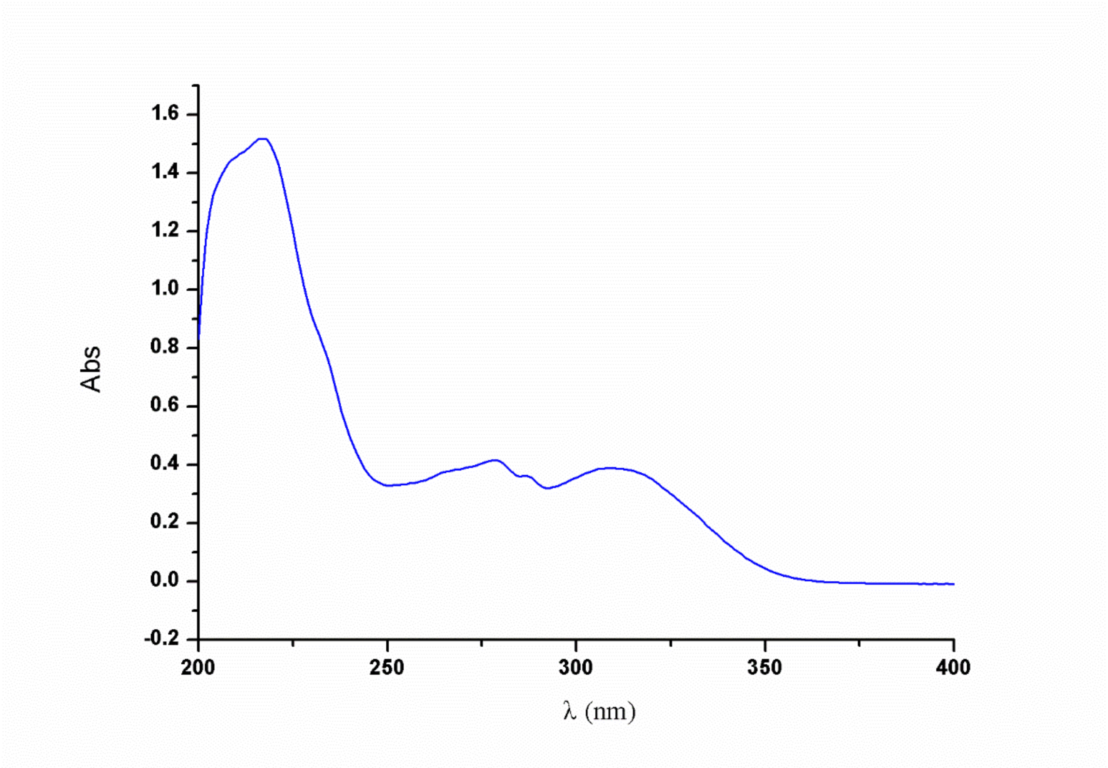

Figure S18. UV spectrum of compound **2**.

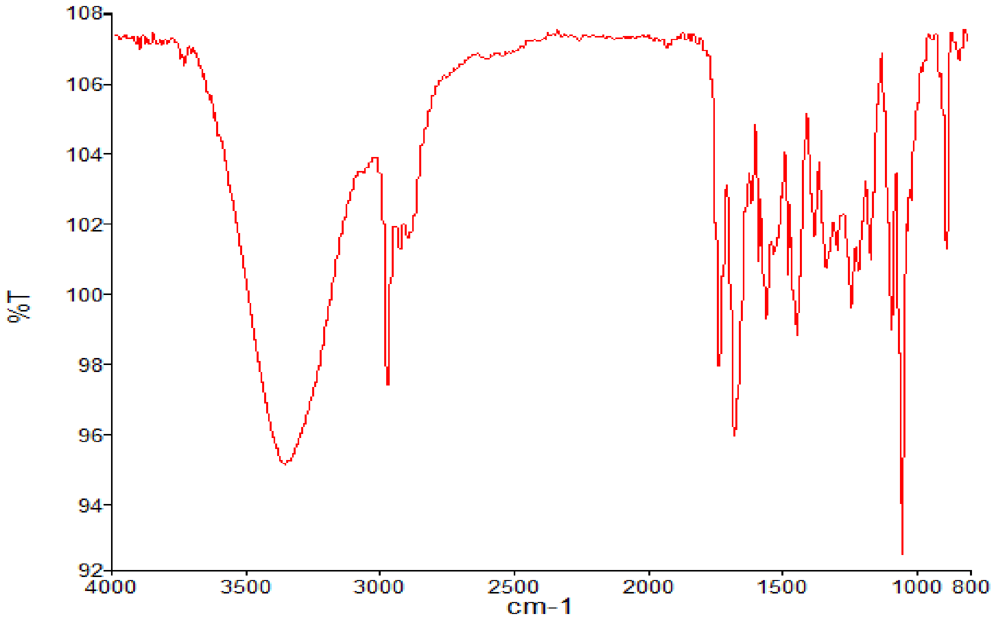

Figure S19. IR spectrum of compound **2**.

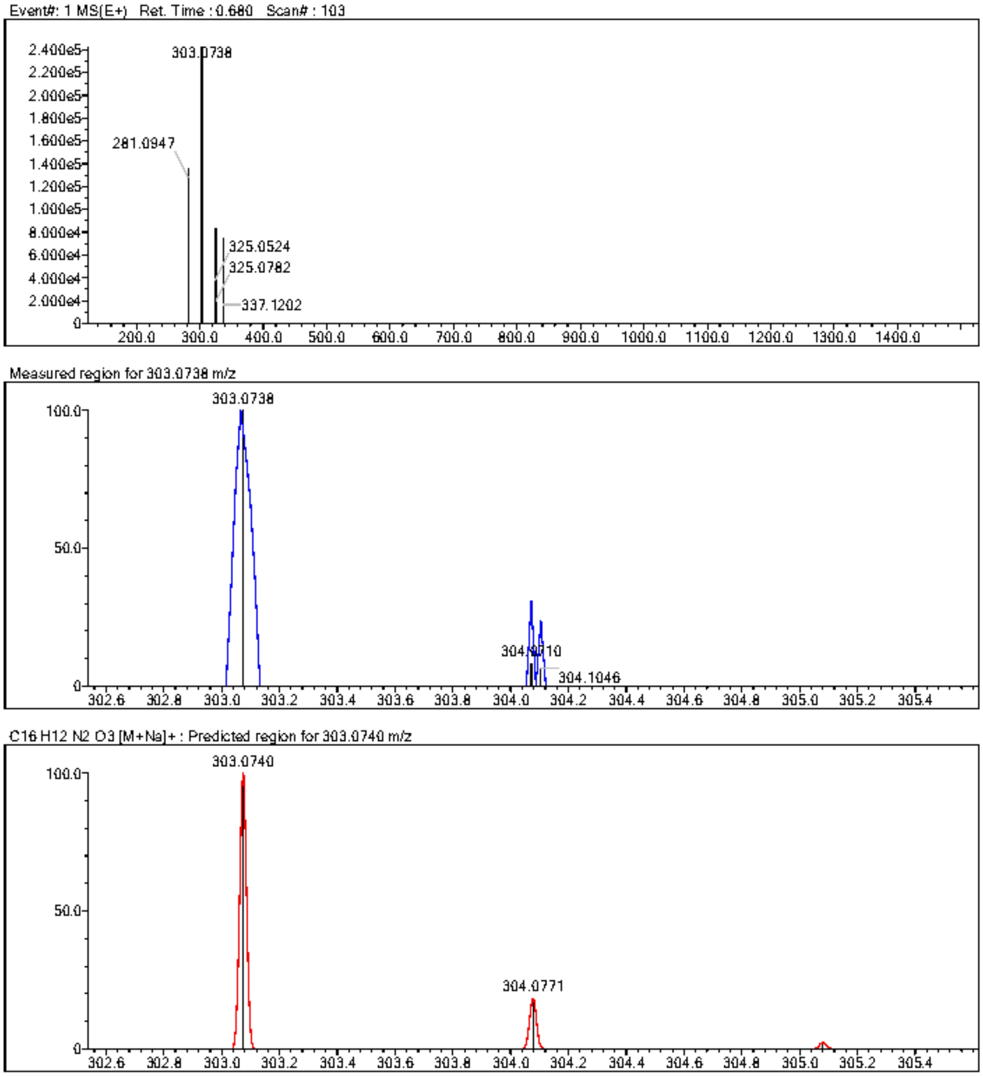

Rank	Score	Formula (M)	Ion	Meas. m/z	Pred. m/z	Df (mDa)	Df (ppm)	Iso	DBE
1	46.17	C16 H12 N2 O3	[M+Na]+	303.0738	303.0740	-0.2	-0.66	46.17	12.0

Figure S20. HRESIMS spectrum of compound **3**.

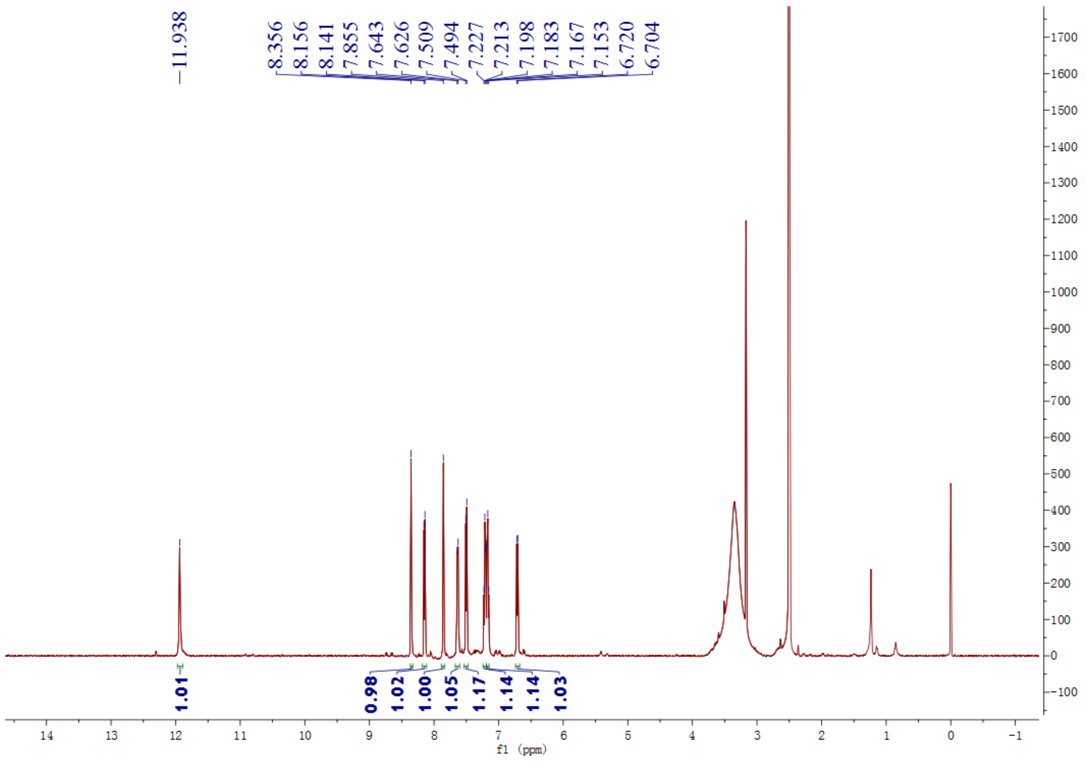

Figure S21. ^{1}H NMR spectrum of compound **3** in DMSO-d_6 (500 MHz).

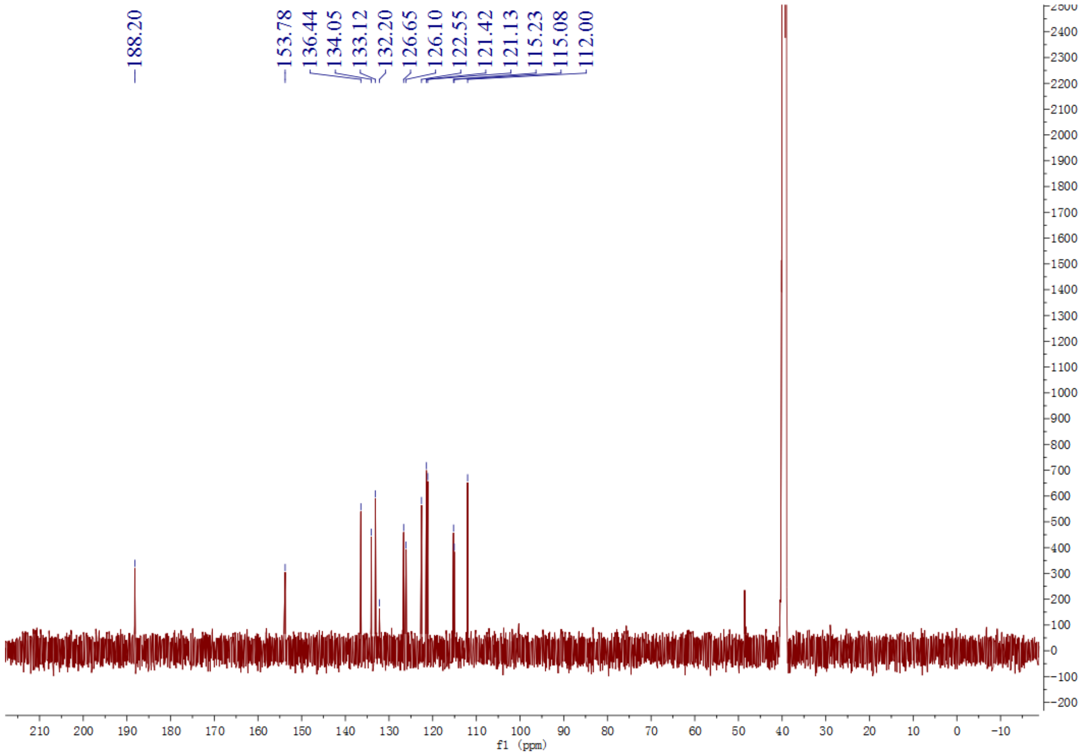

Figure S22. ^{13}C NMR spectrum of compound **3** in DMSO-d_6(125 MHz).

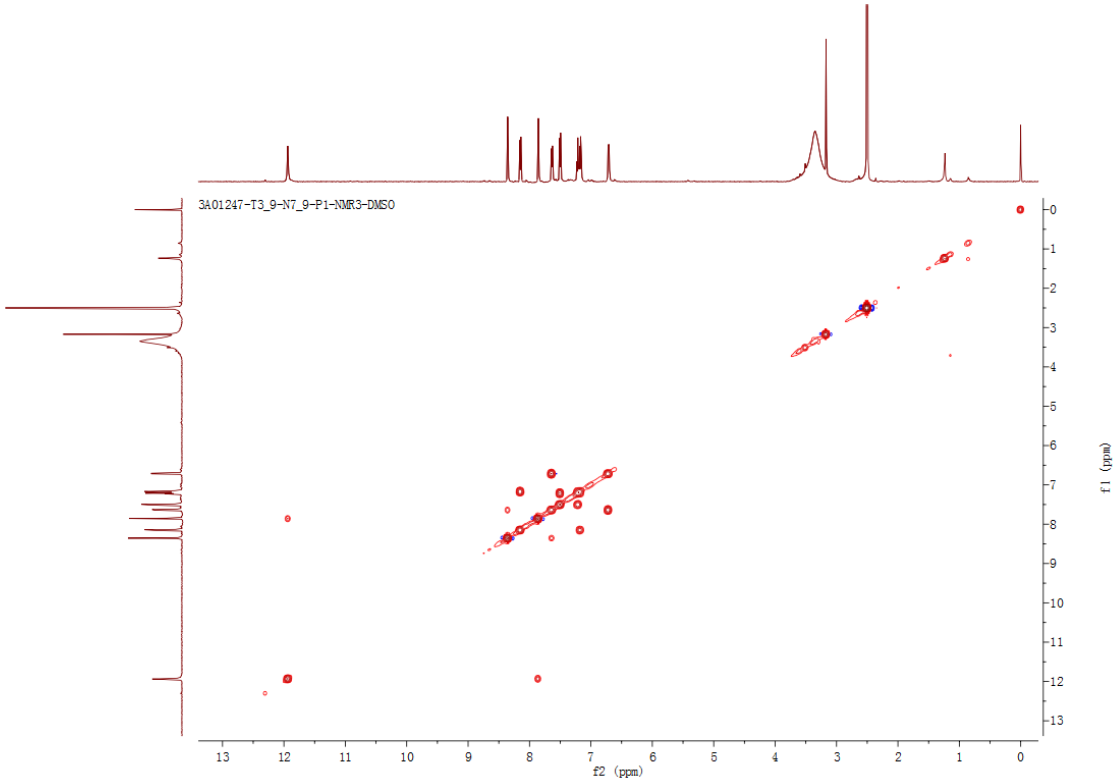

Figure S23. ^{1}H-^{1}H COSY spectrum of compound **3** in DMSO-d_6.

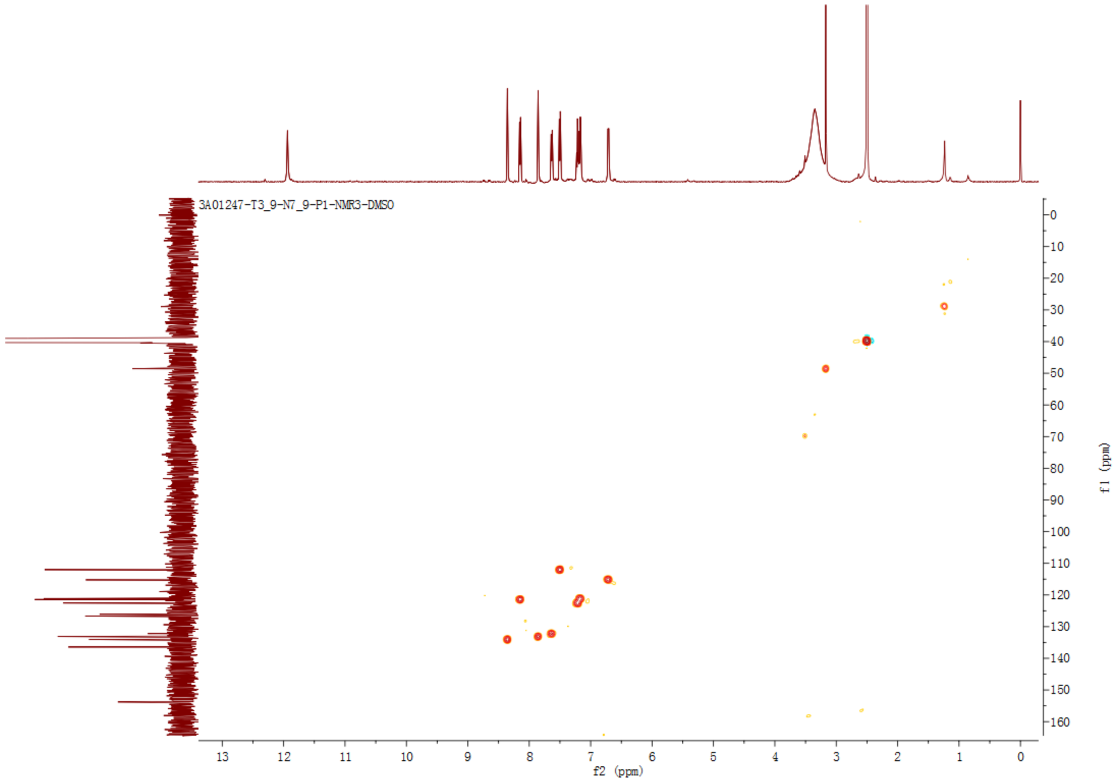

Figure S24. HSQC spectrum of compound **3** in DMSO-d_6.

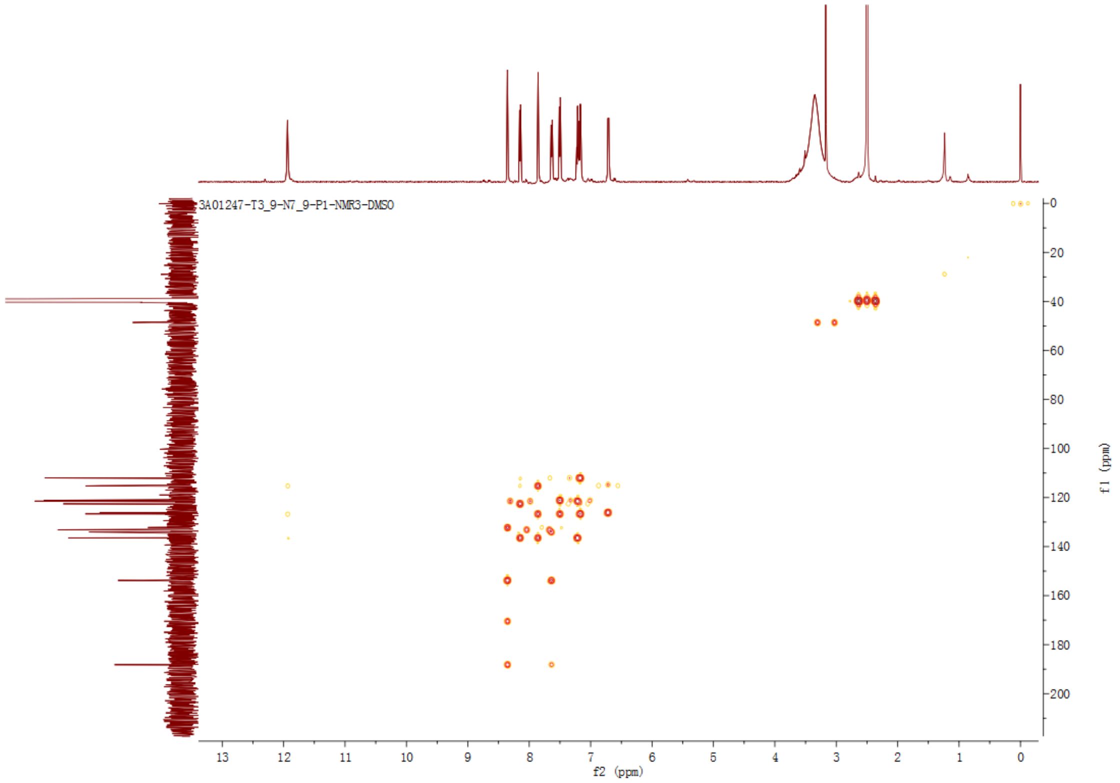

Figure S25. HMBC spectrum of compound **3** in DMSO-d_6.

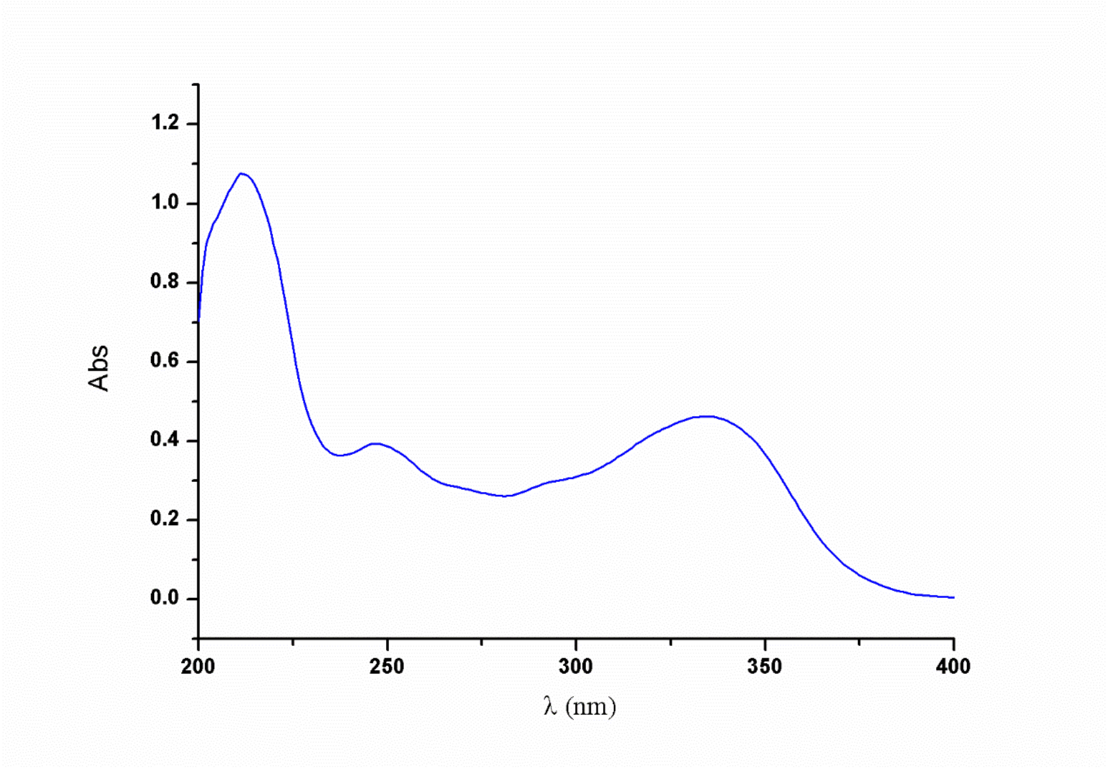

Figure S26. UV spectrum of compound **3**.

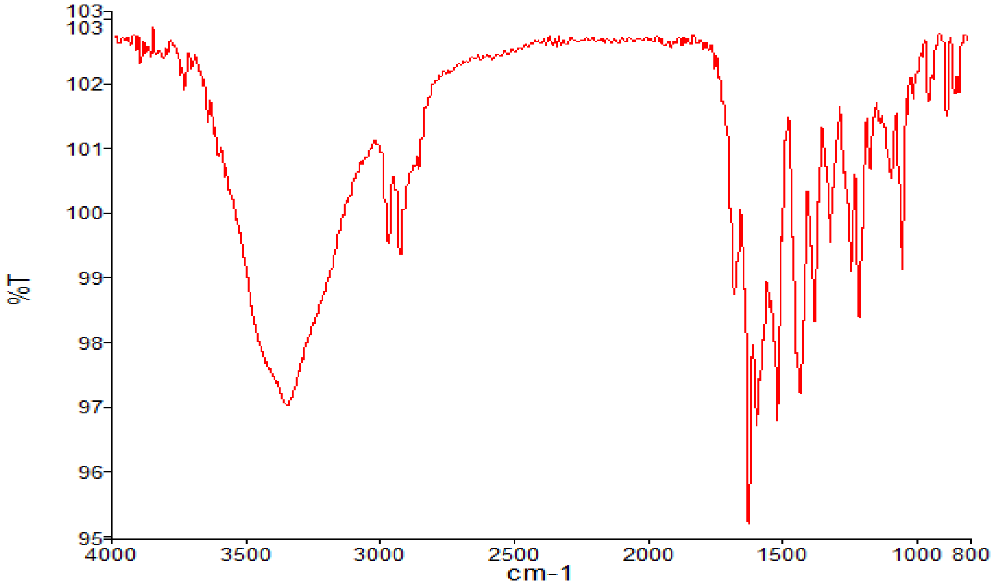

Figure S27. IR spectrum of compound **3**.

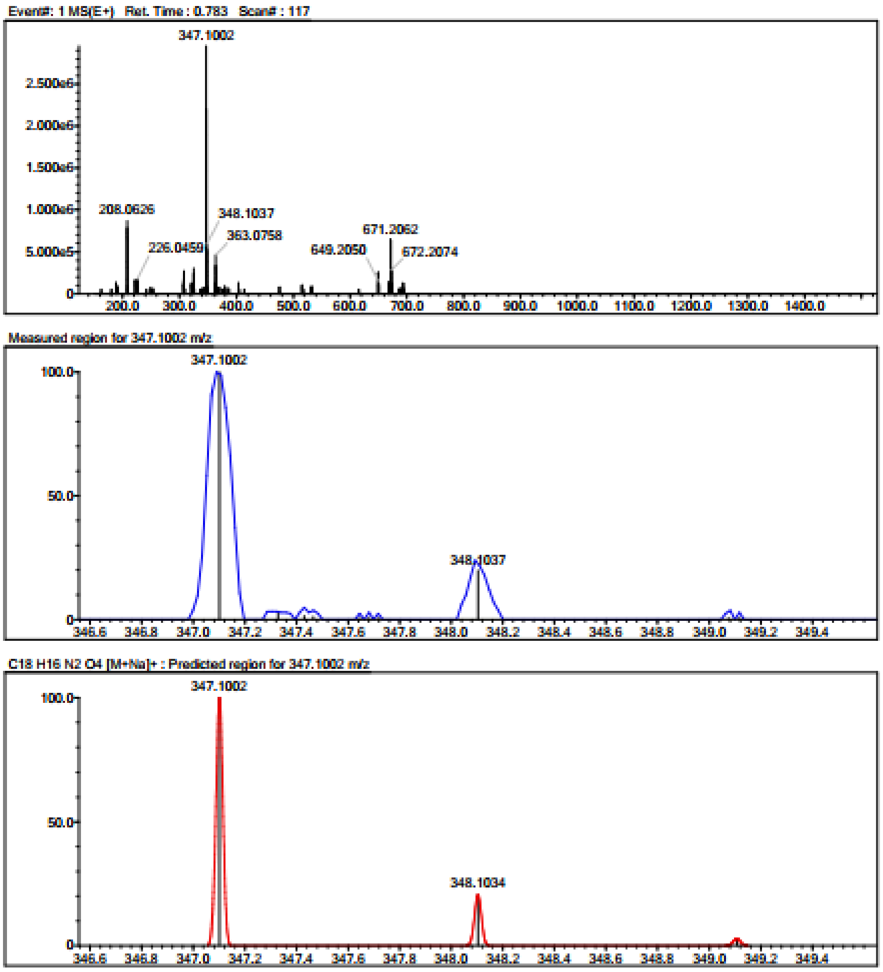

Rank	Score	Formula (M)	Ion	Meas. m/z	Pred. m/z	Df. (mDa)	Df. (ppm)	Iso	DBE
1	80.63	C18 H16 N2 O4	[M+Na]+	347.1002	347.1002	-0.0	0.00	80.63	12.0

Figure S28. HRESIMS spectrum of compound **4**.

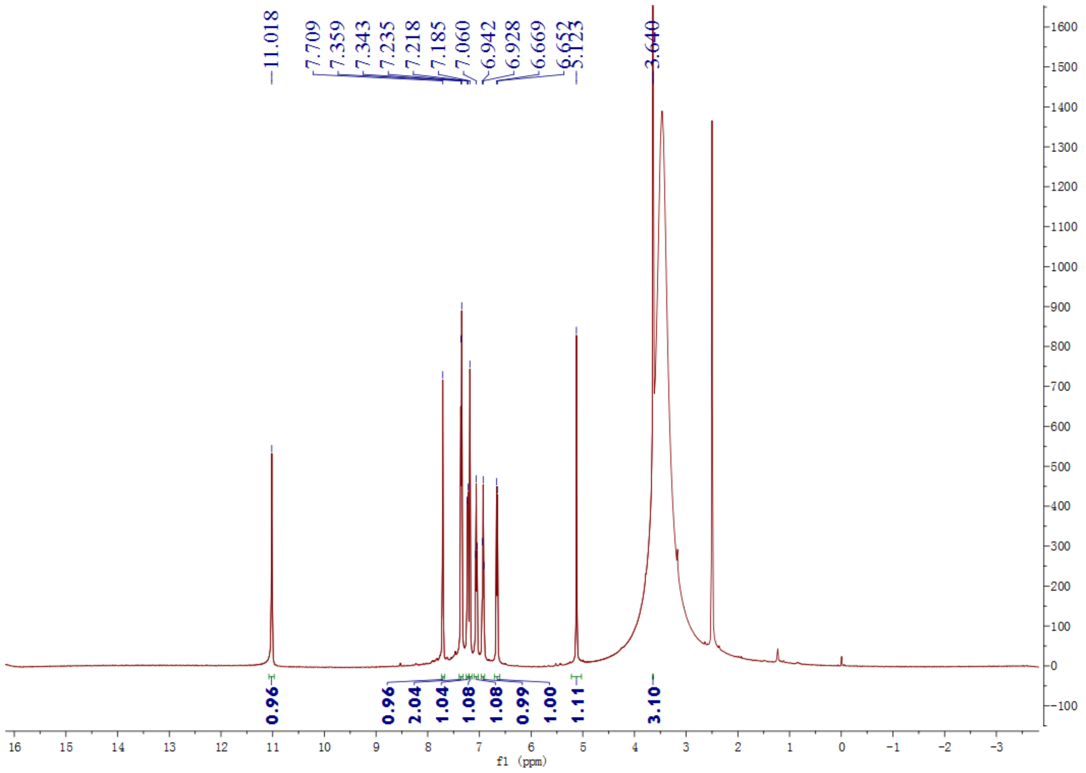

Figure S29. ^{1}H NMR spectrum of compound **4** in DMSO-d_6 (500 MHz).

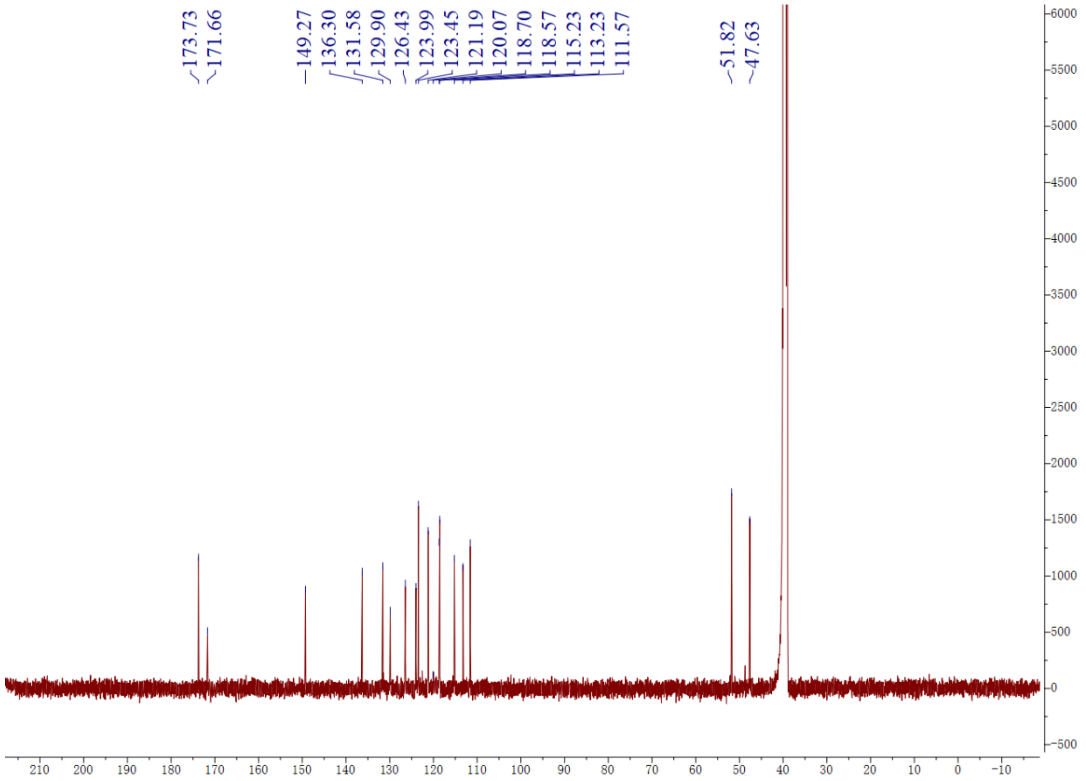

Figure S30. ^{13}C NMR spectrum of compound **4** in DMSO-d_6(125 MHz).

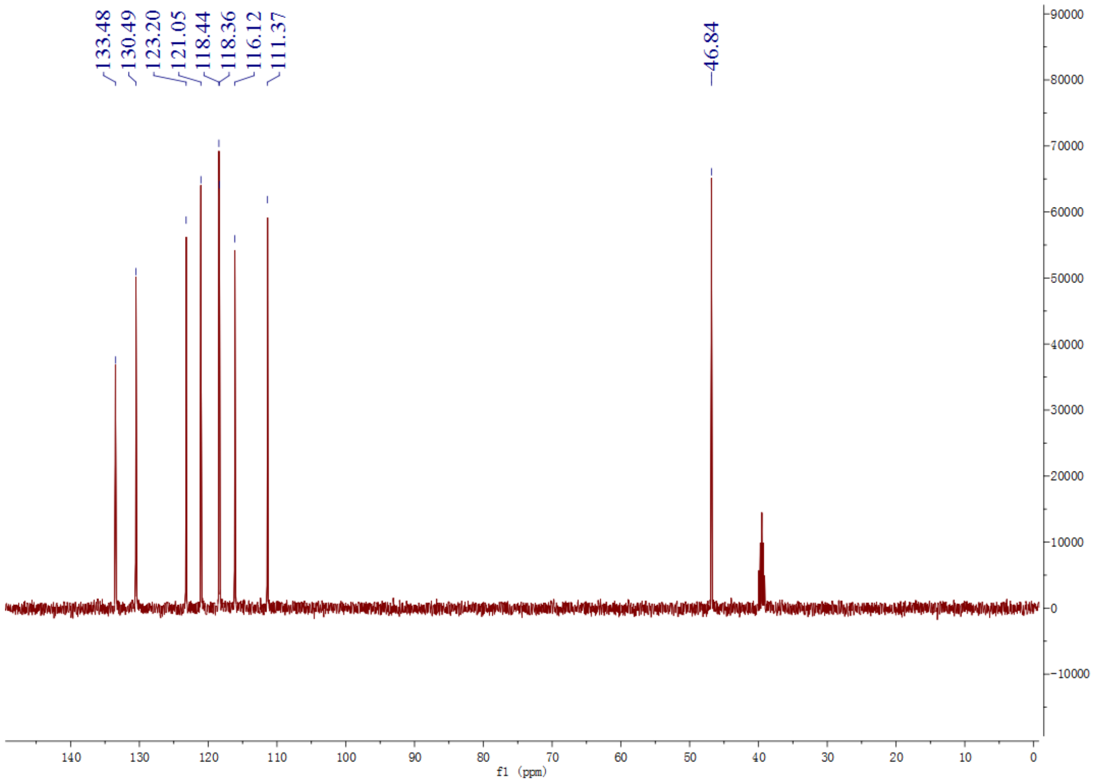

Figure S31. DEPT 90 spectrum of compound **4** in DMSO-d_6 (400 MHz).

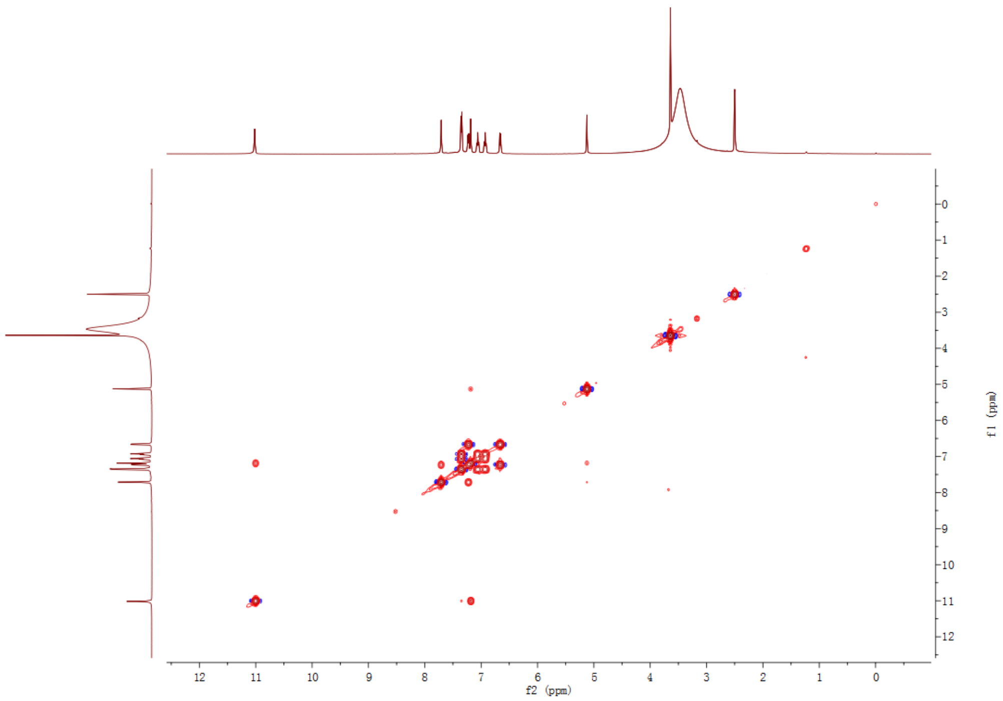

Figure S32. ^{1}H-^{1}H COSY spectrum of compound **4** in DMSO-d_6.

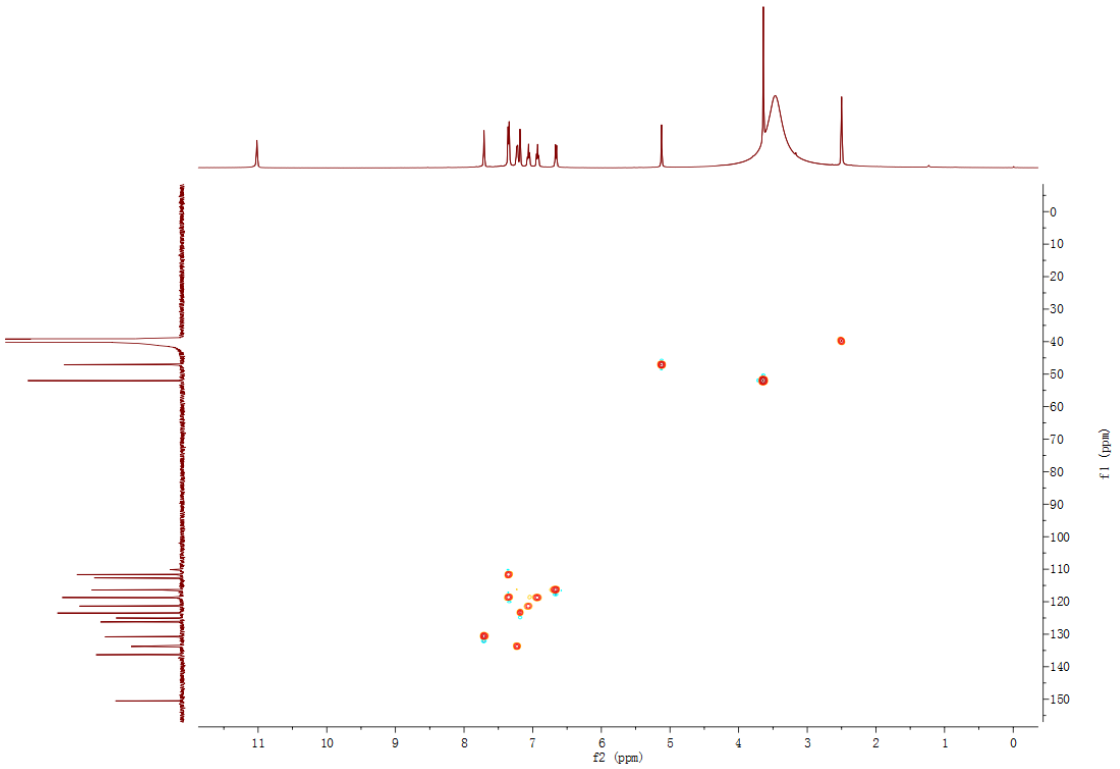

Figure S33. HSQC spectrum of compound **4** in DMSO-*d*6.

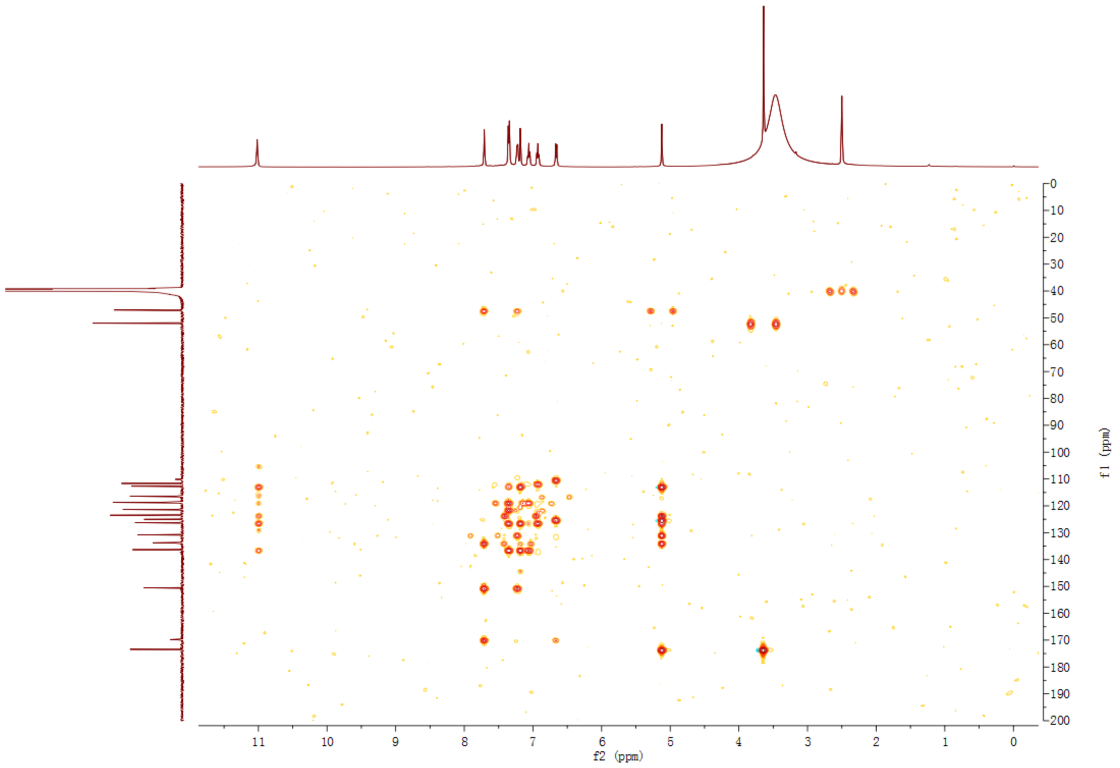

Figure S34. HMBC spectrum of compound **4** in DMSO-*d*6.

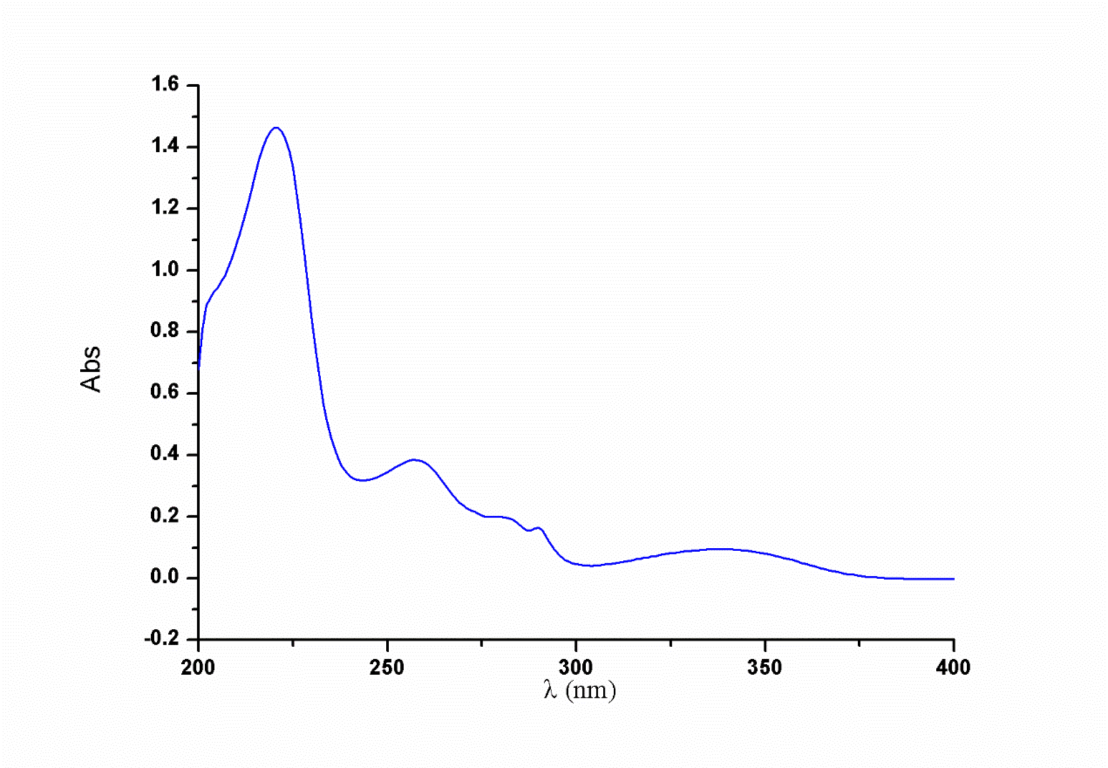

Figure S35. UV spectrum of compound **4**.

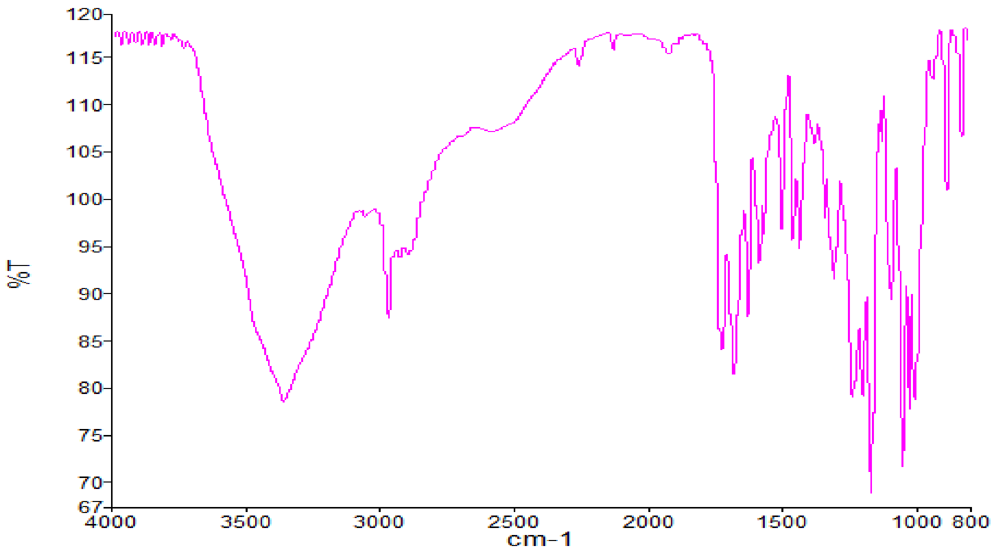

Figure S36. IR spectrum of compound **4**.

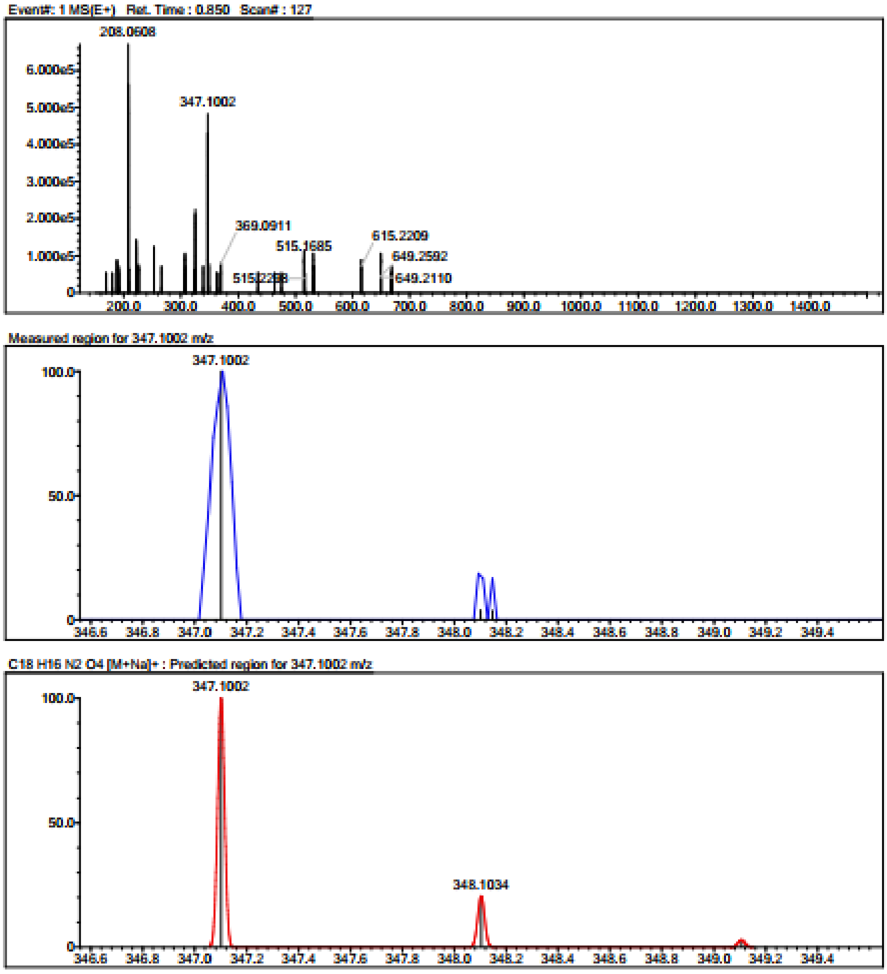

Rank	Score	Formula (M)	Ion	Meas. m/z	Pred. m/z	Df. (mDa)	Df. (ppm)	Iso	DBE
1	64.83	C18 H16 N2 O4	[M+Na]+	347.1002	347.1002	-0.0	0.00	64.83	12.0

Figure S37. HRESIMS spectrum of compound **5**.

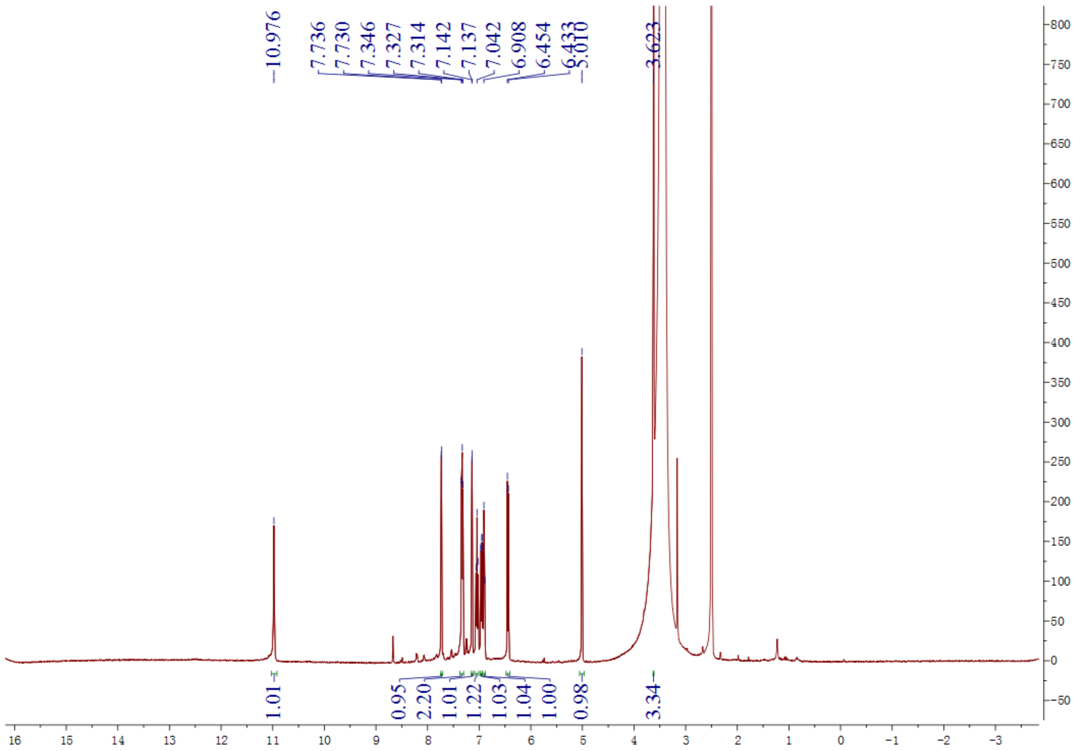

Figure S38. ^{1}H NMR spectrum of compound **5** in DMSO-d_6(500 MHz).

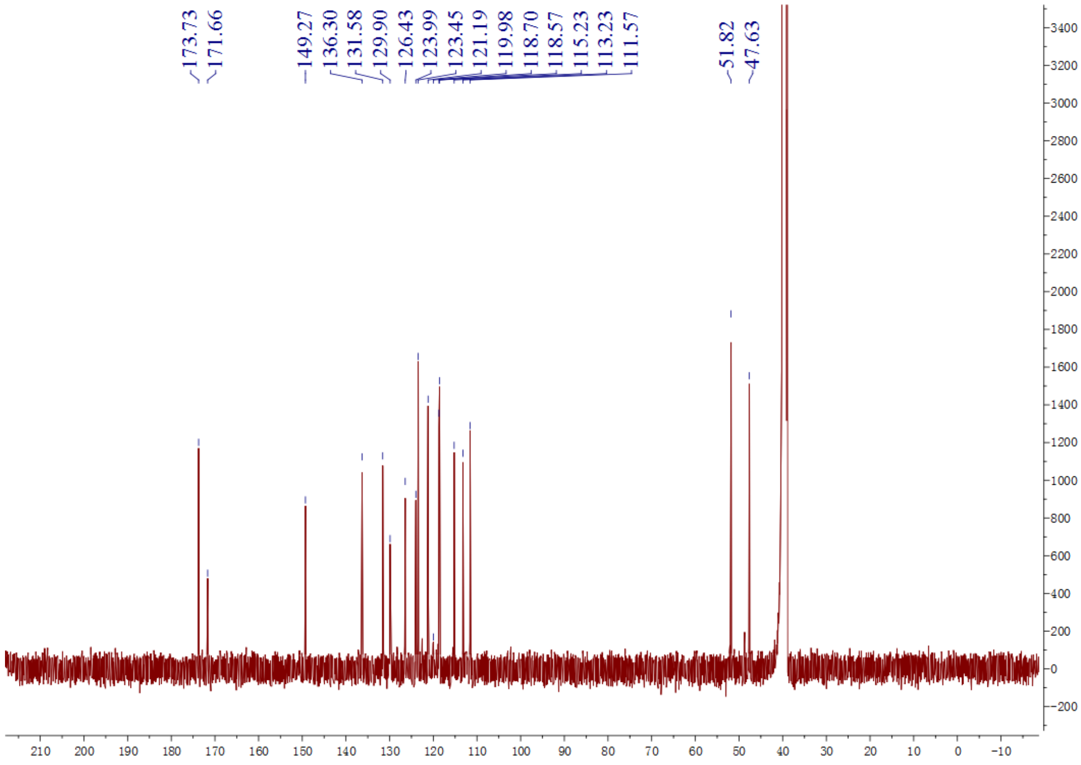

Figure S39. ^{13}C NMR spectrum of compound **5** in DMSO-d_6(125MHz).

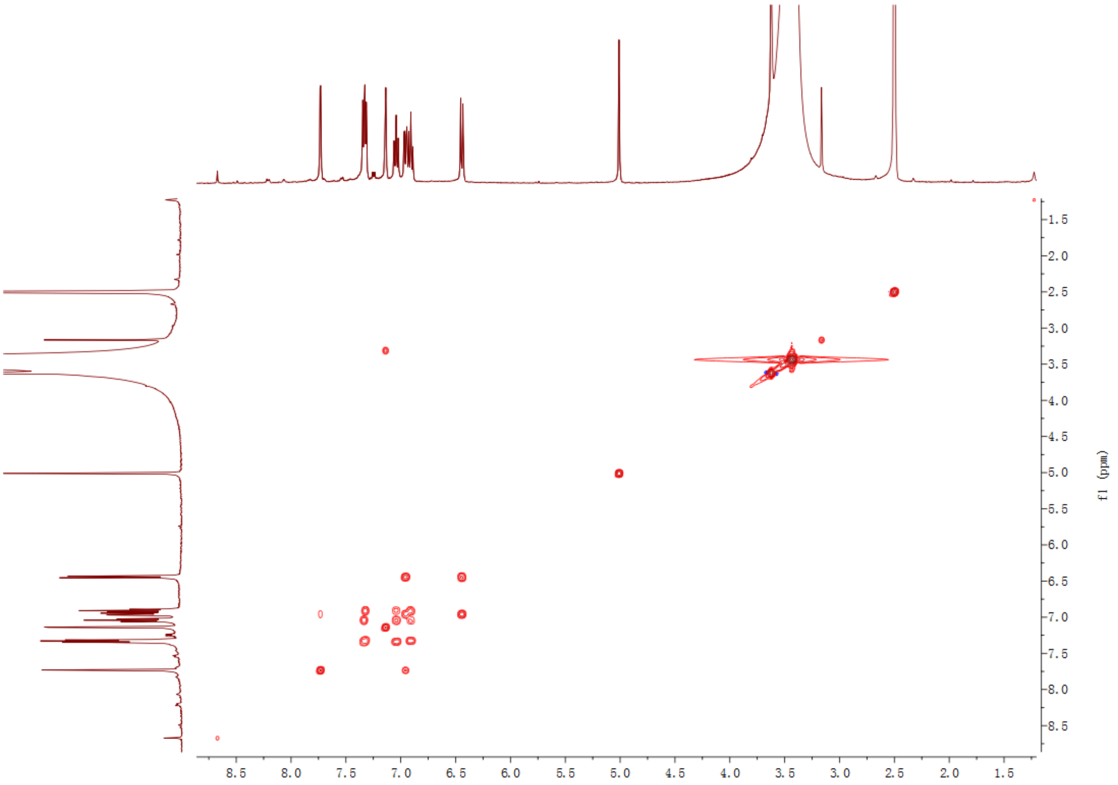

Figure S40. ^{1}H-^{1}H COSY spectrum of compound **5** in DMSO-d_6.

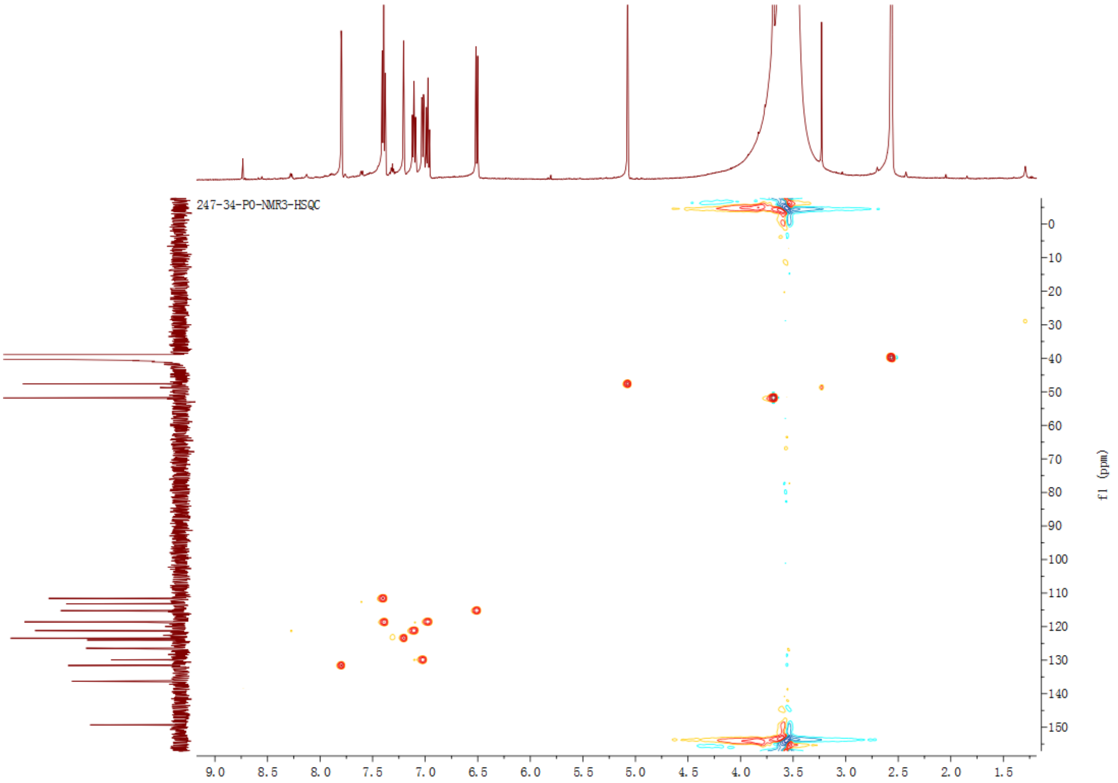

Figure S41. HSQC spectrum of compound **5** in DMSO-d_6.

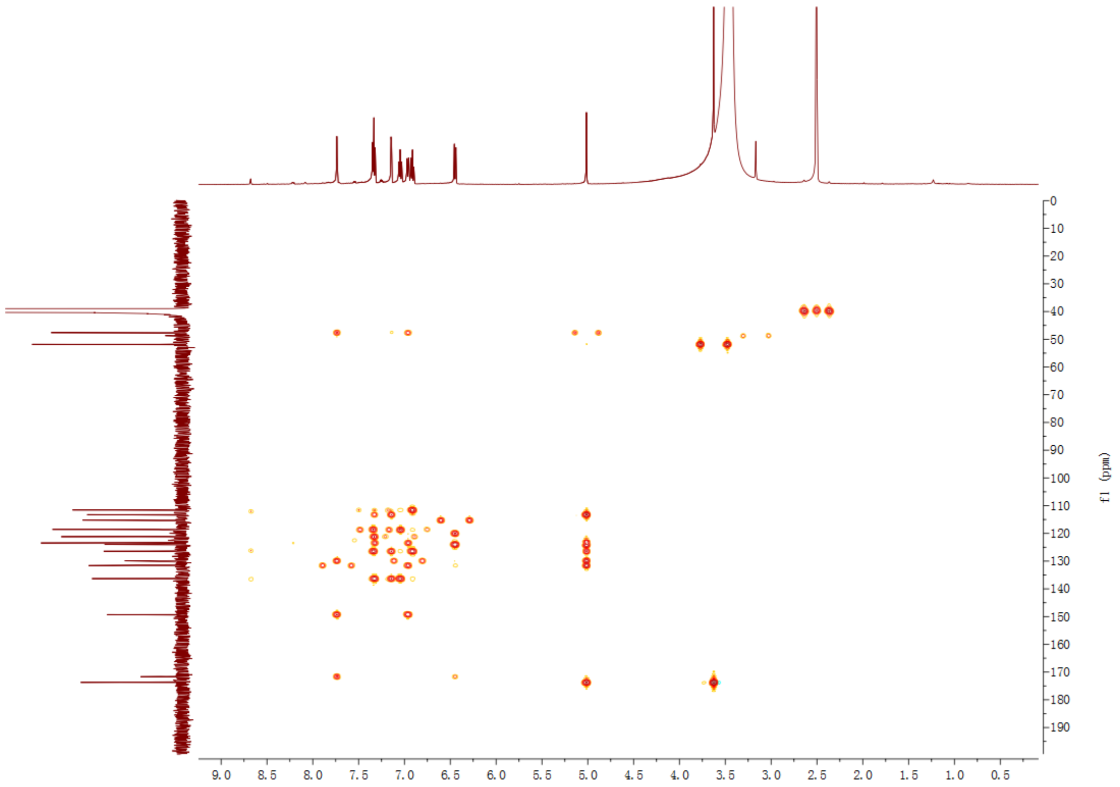

Figure S42. HMBC spectrum of compound **5** in DMSO-d_6.

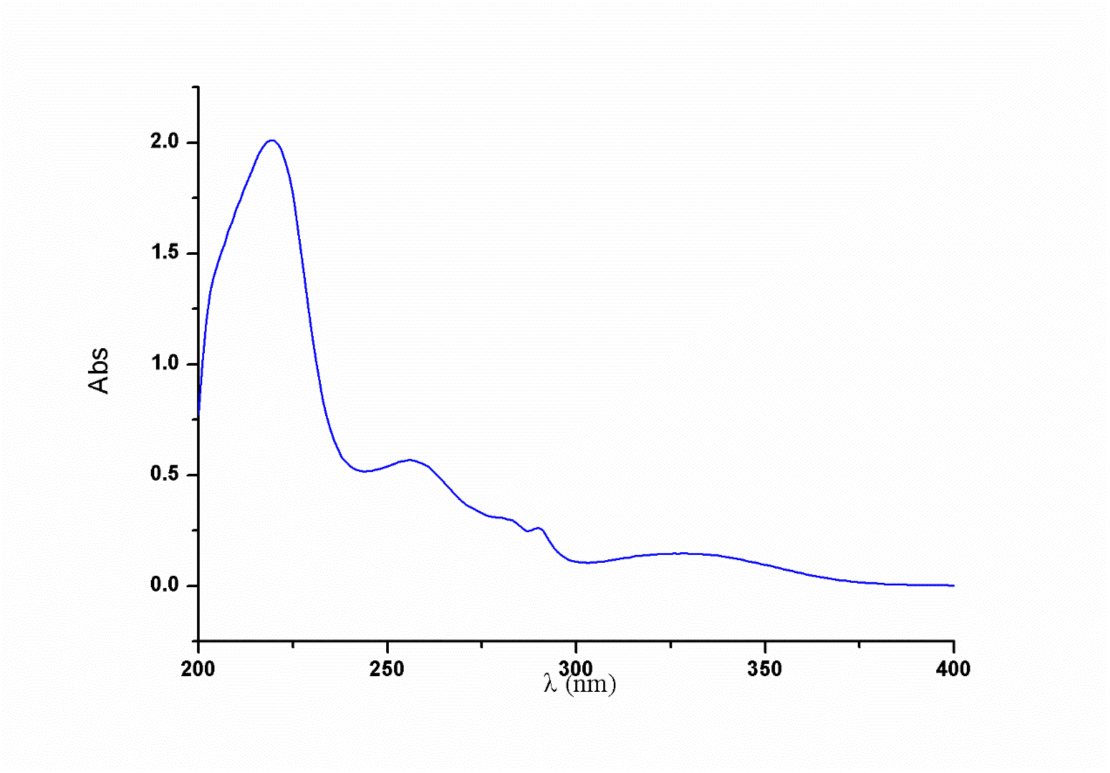

Figure S43. UV spectrum of compound **5**.

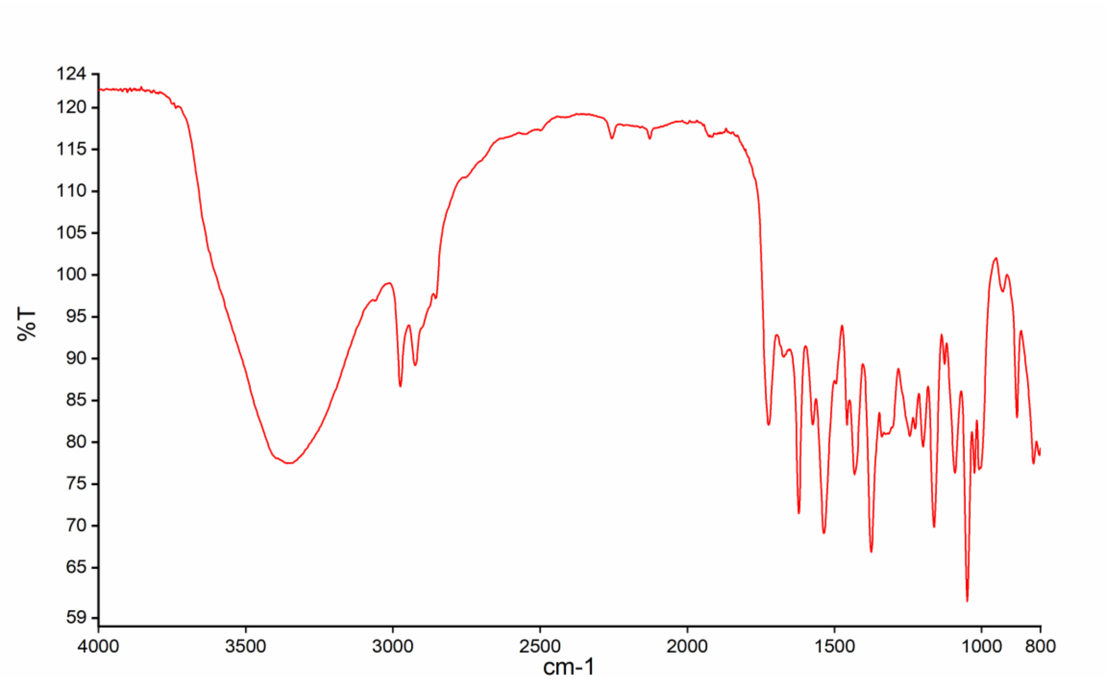

Figure S44. IR spectrum of compound **5**.

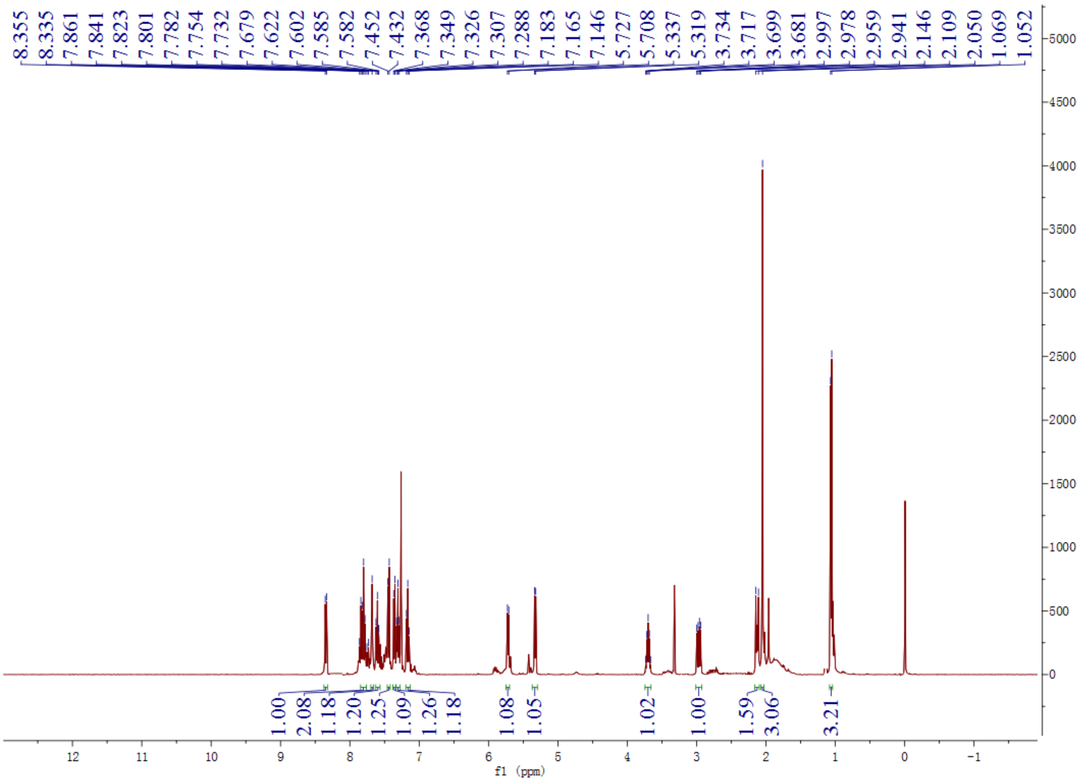

Figure S45. ^{1}H NMR spectrum of compound **6** in $CDCl_3$ (400 MHz).

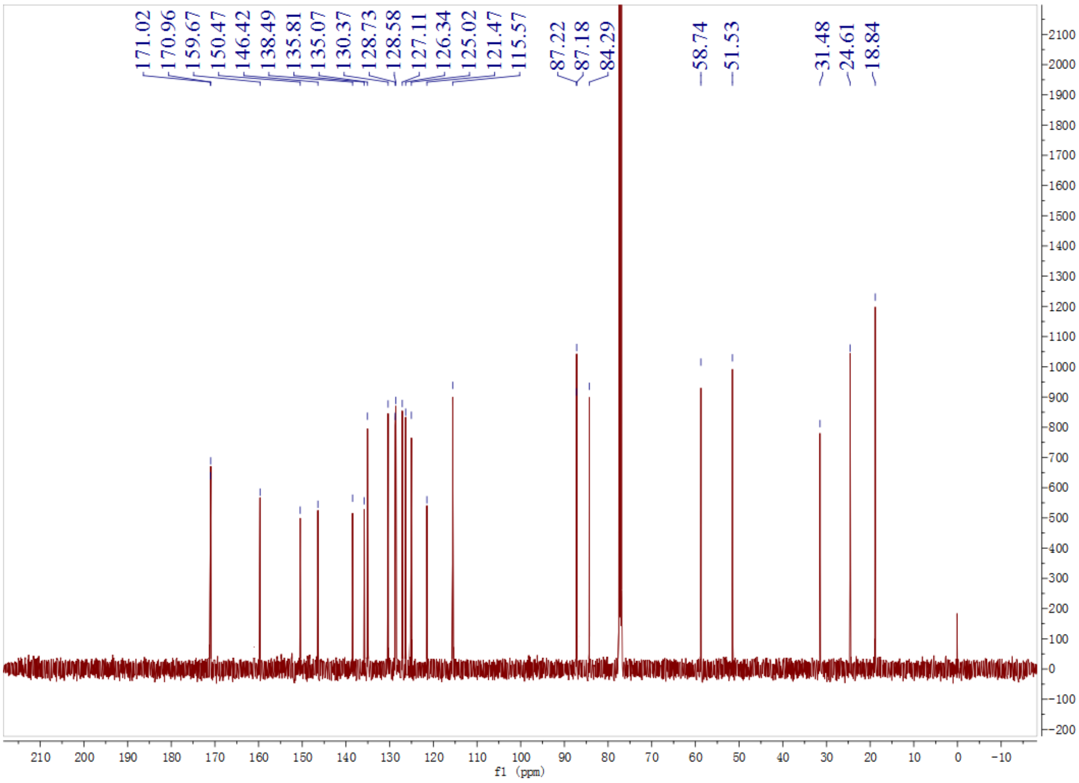

Figure S46. ^{13}C NMR spectrum of compound **6** in $CDCl_3$ (100 MHz).

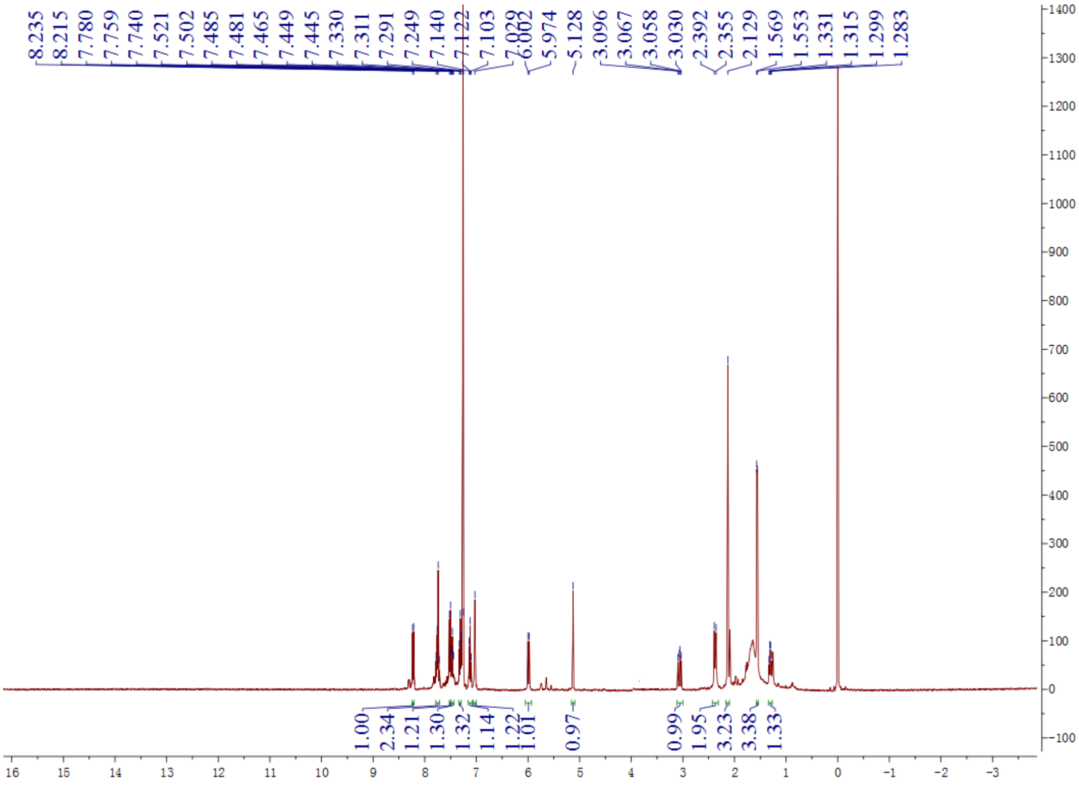

Figure S47. ^{1}H NMR spectrum of compound **7** in DMSO-d_6 (400 MHz).

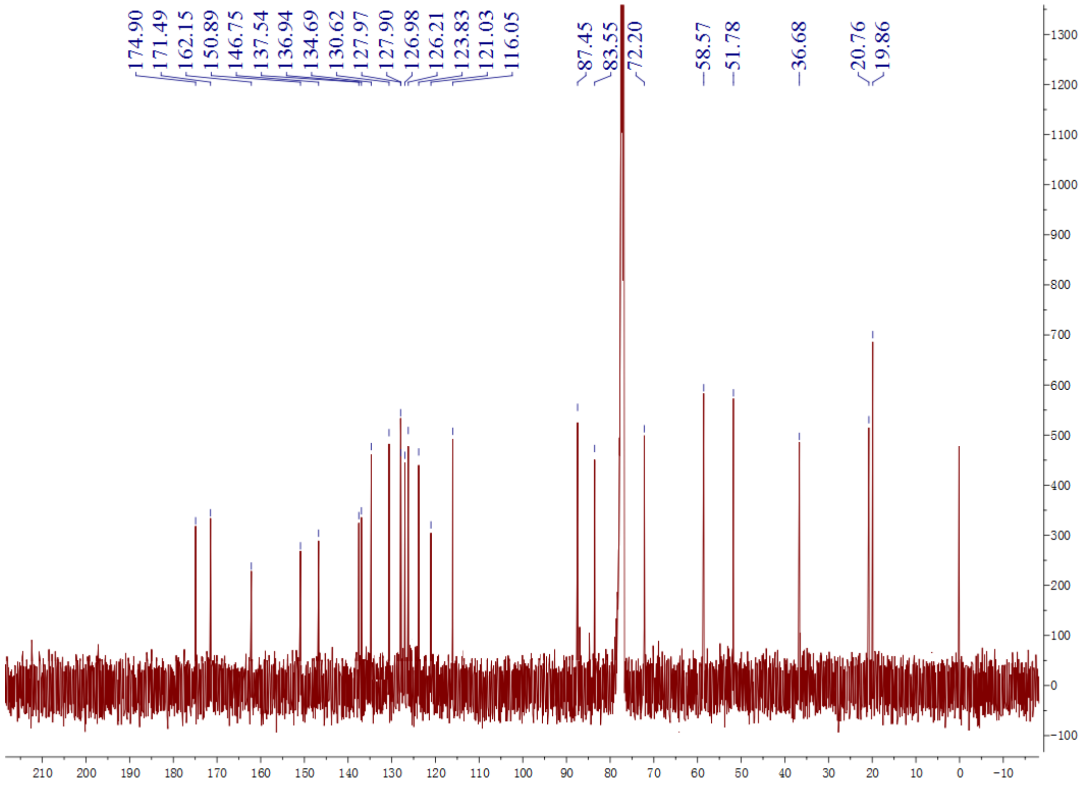

Figure S48. ^{13}C NMR spectrum of compound **7** in DMSO-d_6 (100 MHz).

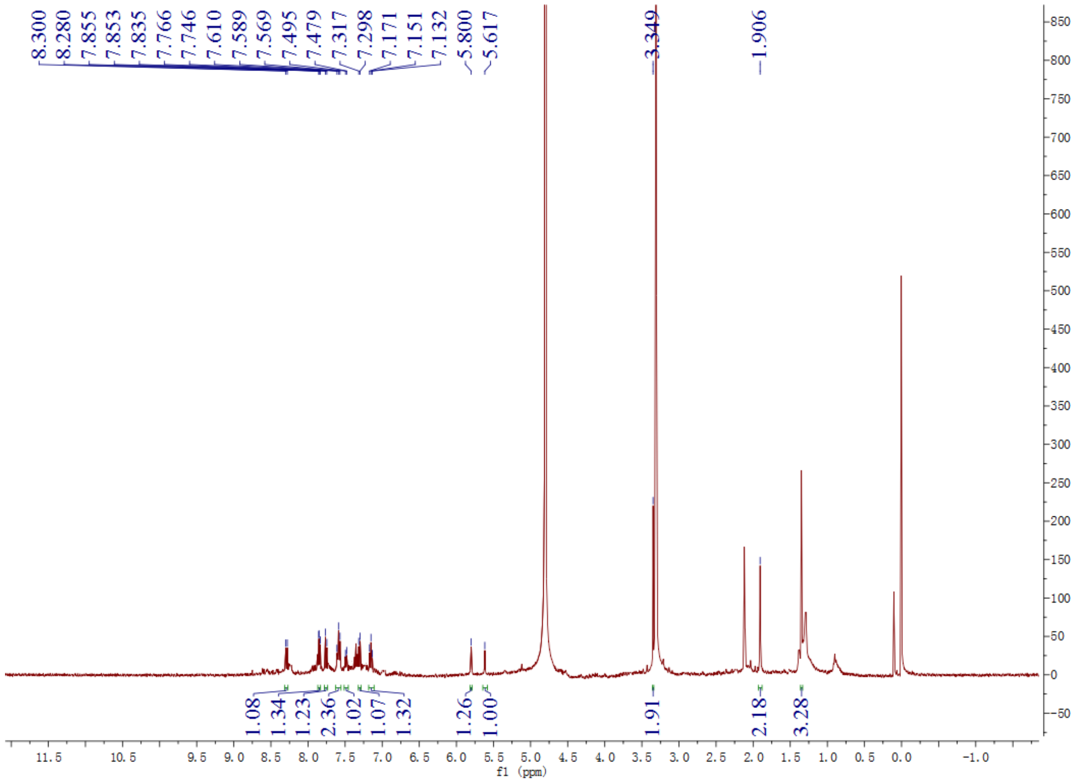

Figure S49. ^{1}H NMR spectrum of compound **8** in CD_3OD (400 MHz).

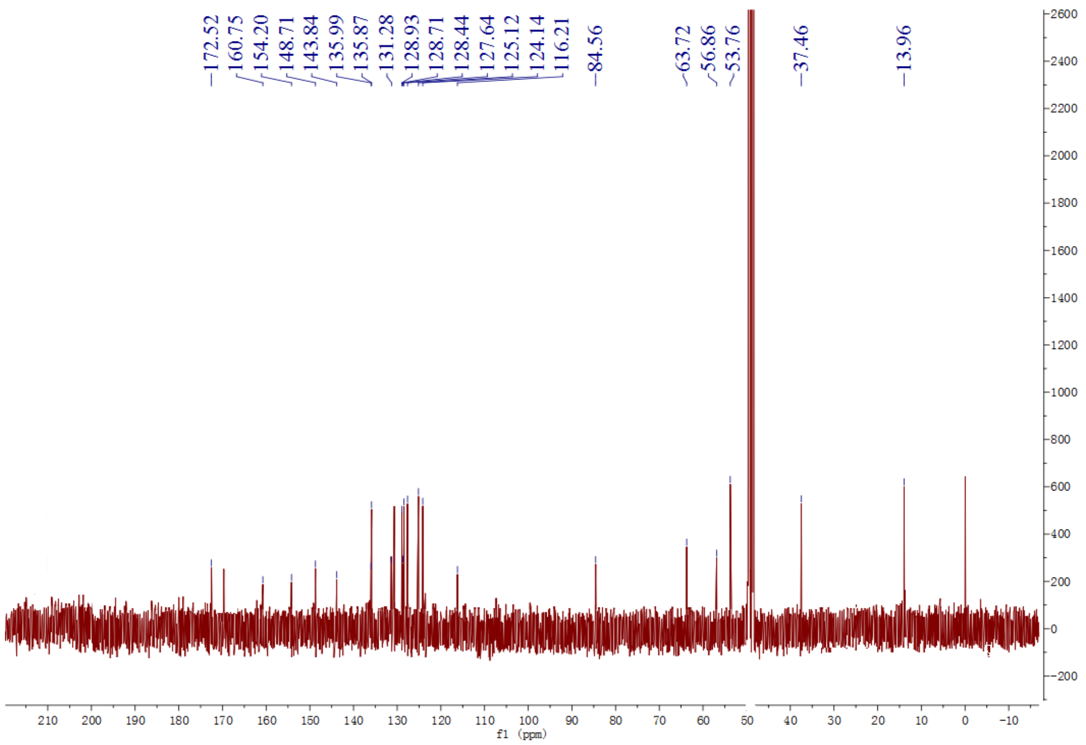

Figure S50. ^{13}C NMR spectrum of compound **8** in CD_3OD (100 MHz).

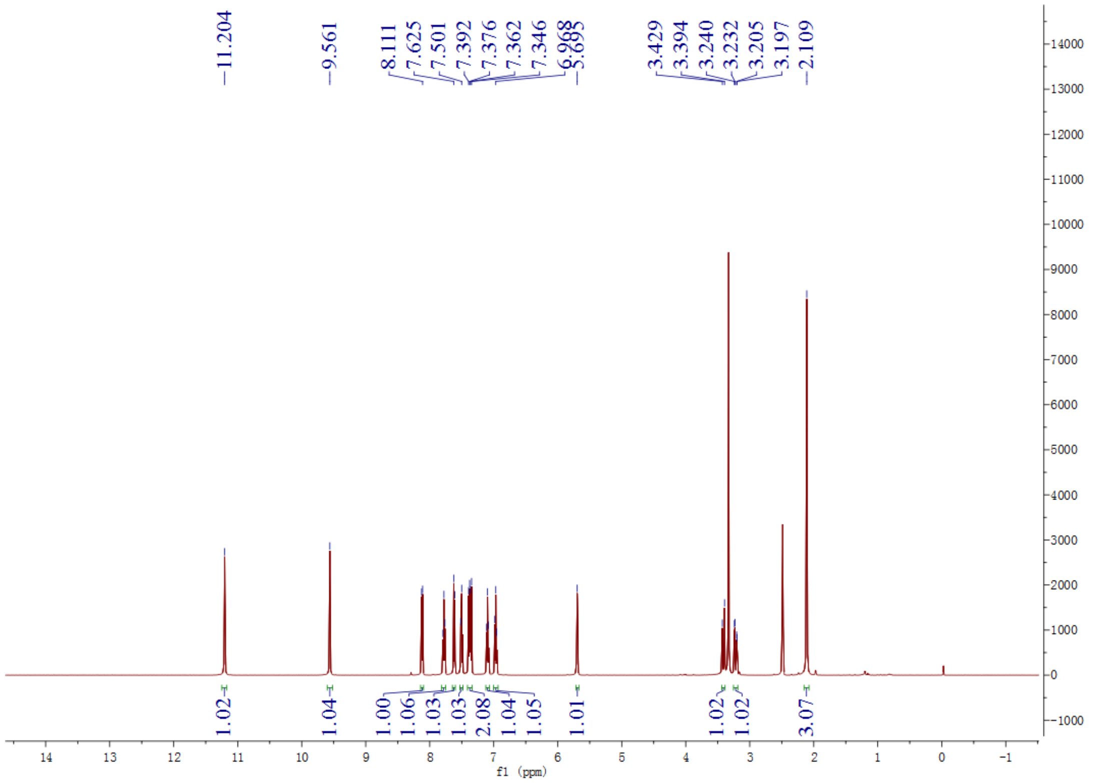

Figure S51. ^{1}H NMR spectrum of compound **9** in DMSO-d_6 (500 MHz).

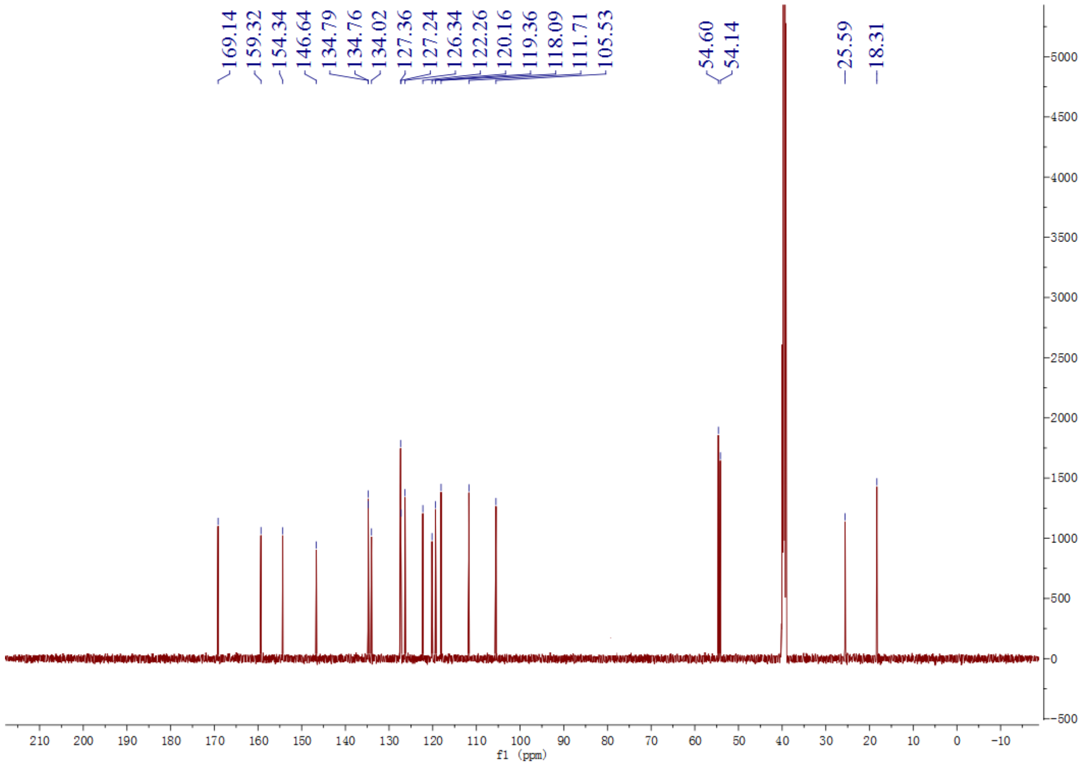

Figure S52. ^{13}C NMR spectrum of compound **9** in DMSO-d_6 (125 MHz).

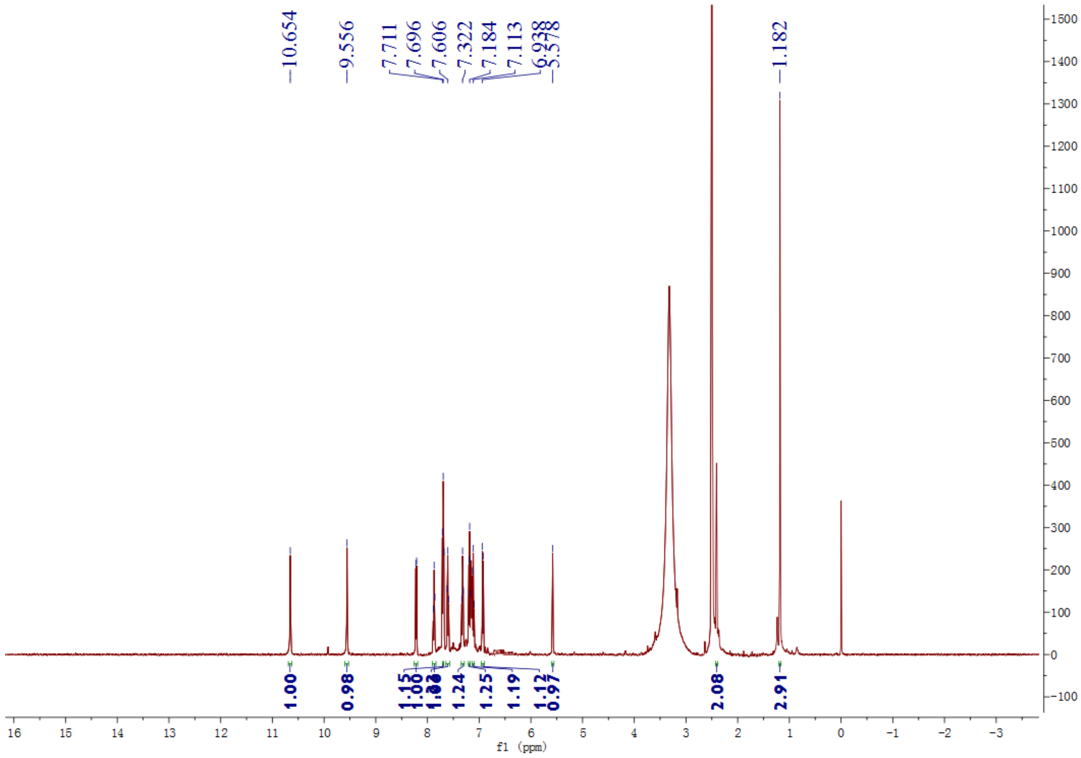

Figure S53. ^{1}H NMR spectrum of compound **10** in DMSO-d_6 (500 MHz).

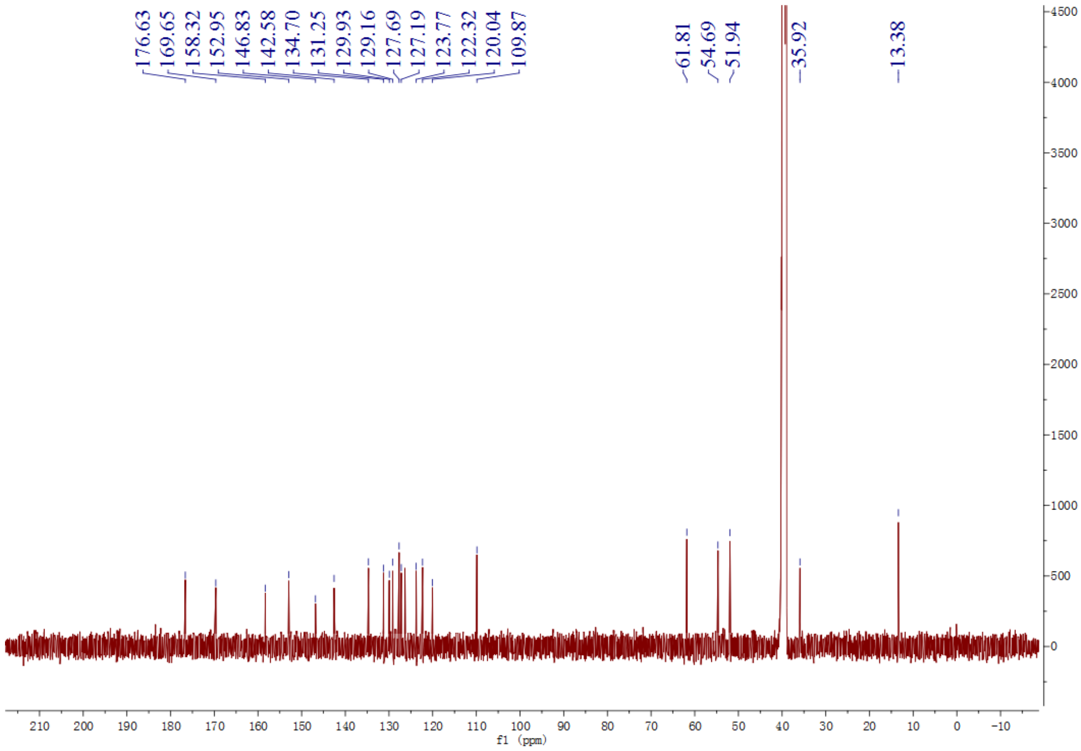

Figure S54. ^{13}C NMR spectrum of compound **10** in DMSO-d_6 (125 MHz).

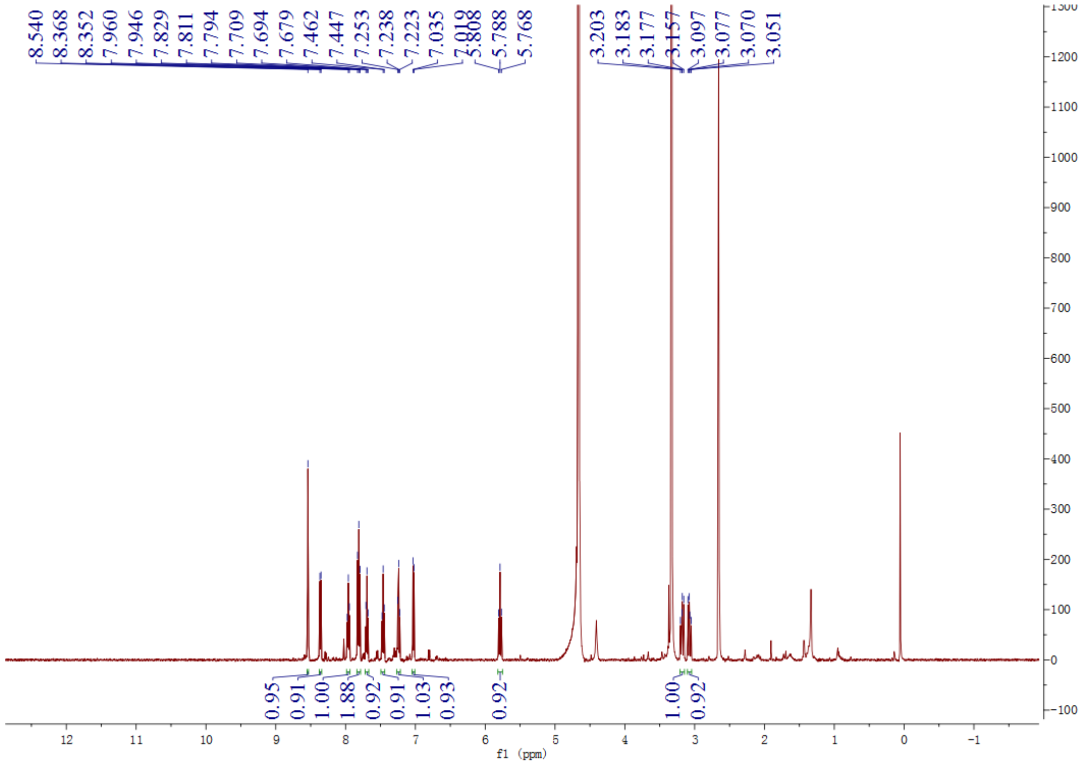

Figure S55. ^{1}H NMR spectrum of compound **11** in DMSO-d_6 (500 MHz).

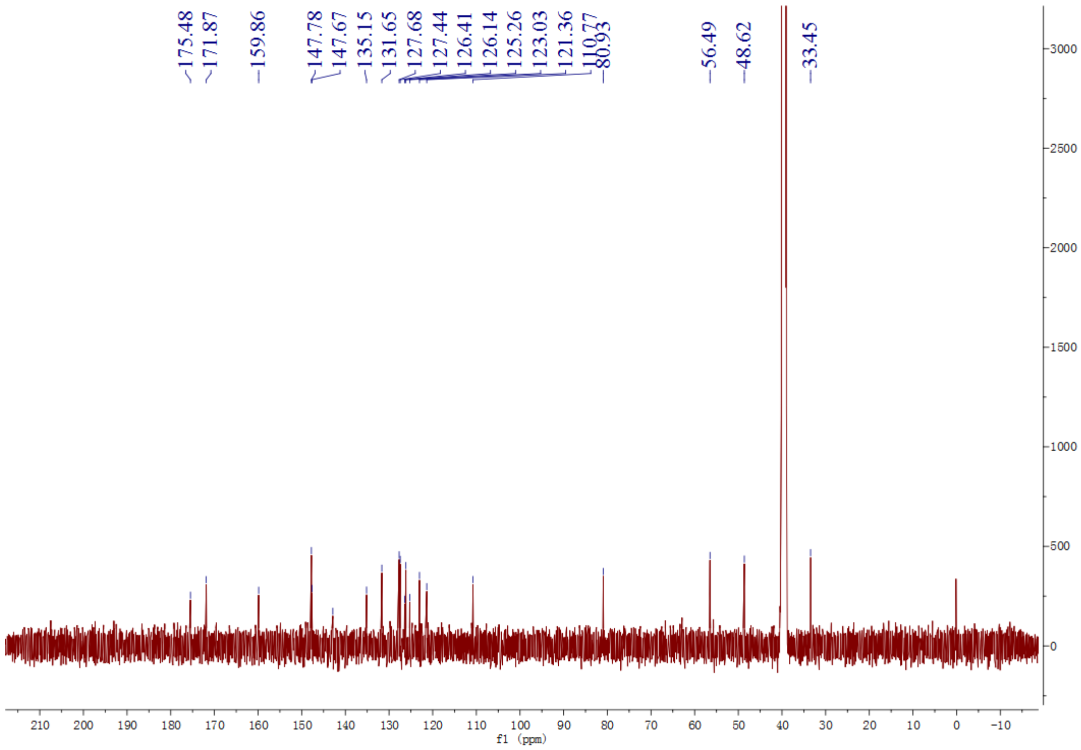

Figure S56. ^{13}C NMR spectrum of compound **11** in DMSO-d_6 (125 MHz).

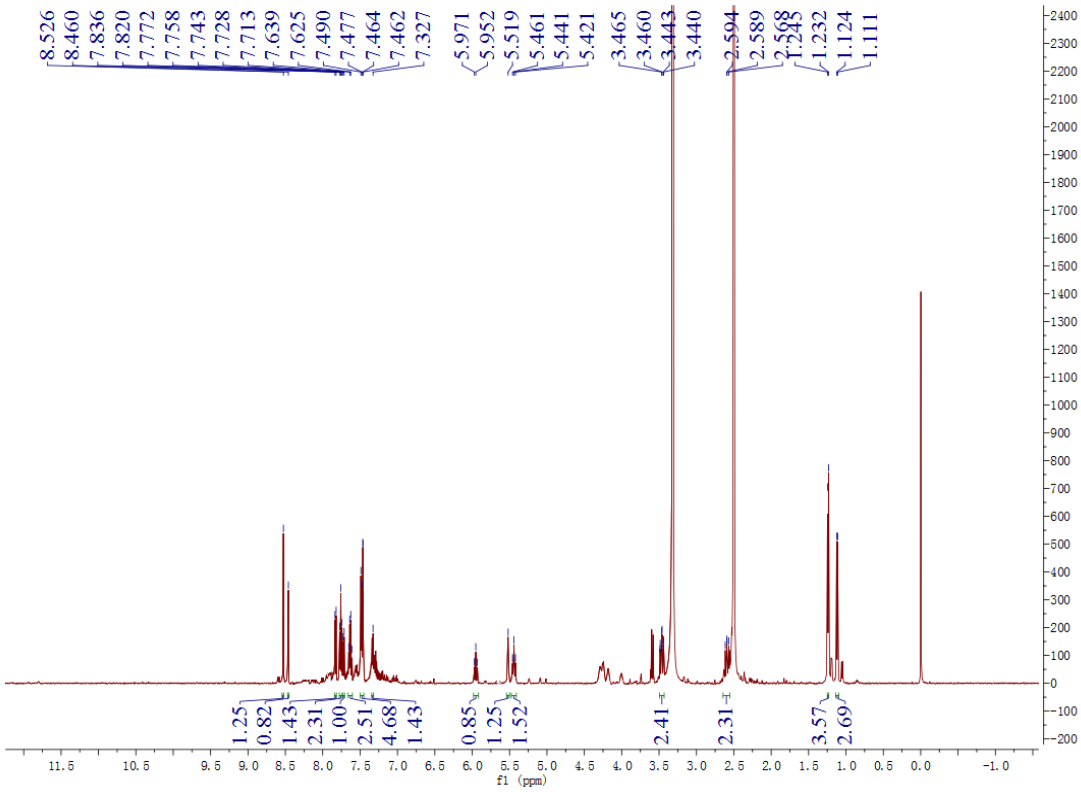

Figure S57. ^{1}H NMR spectrum of compound **12** in acetone-d_6 (400 MHz).

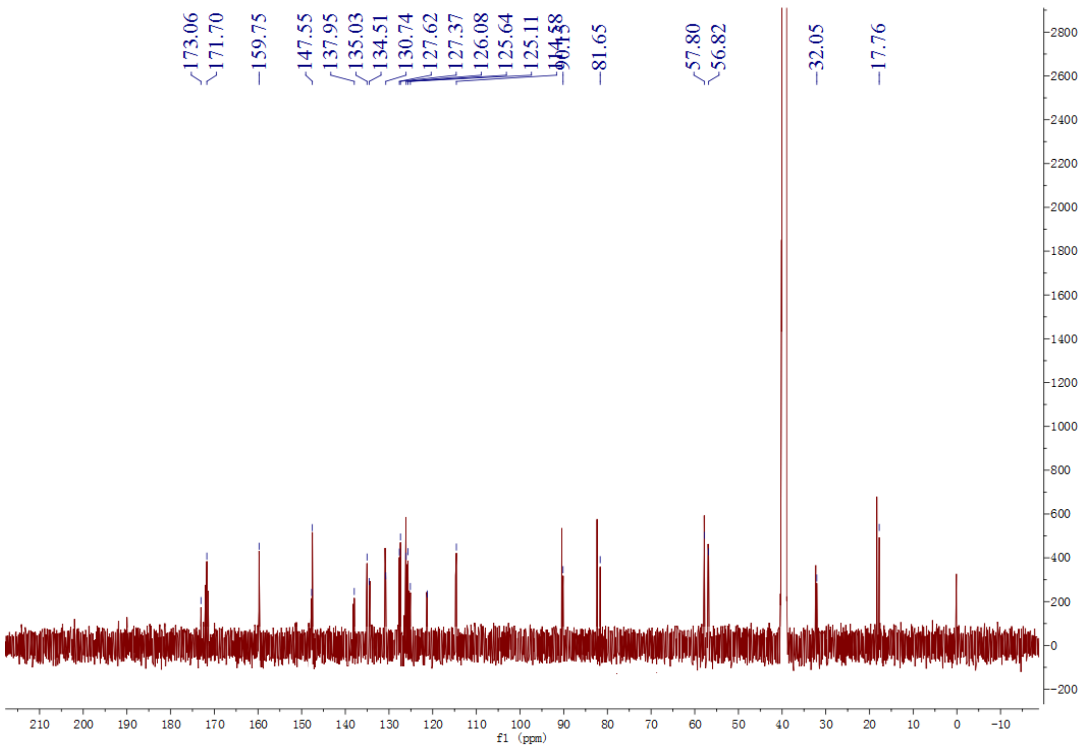

Figure S58. ^{13}C NMR spectrum of compound **12** in acetone-d_6 (100 MHz).

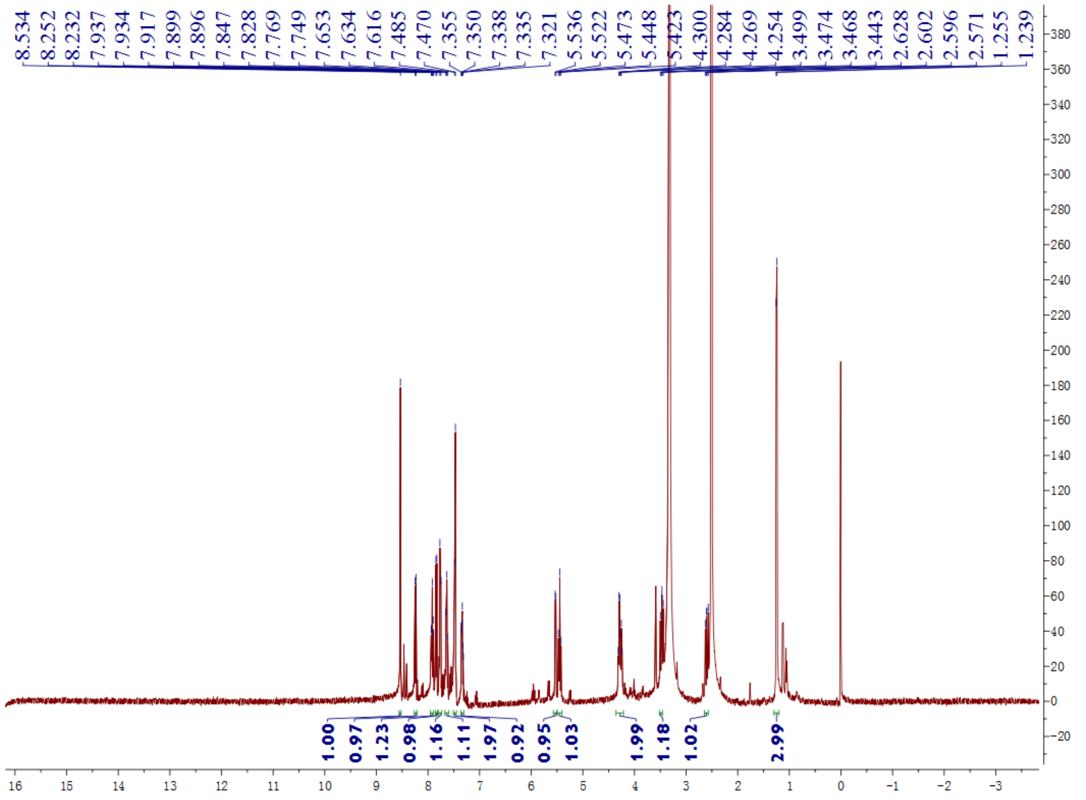

Figure S59. ^{1}H NMR spectrum of compound **13** in DMSO-d_6 (400 MHz).

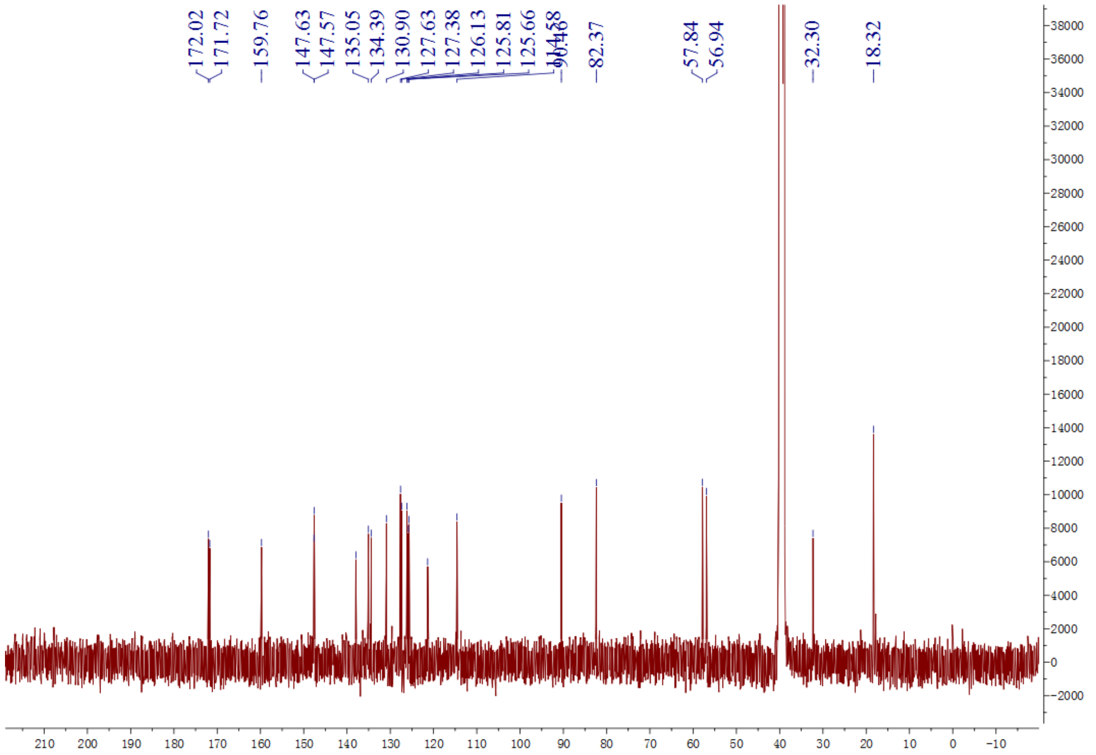

Figure S60. ^{13}C NMR spectrum of compound **13** in DMSO-d_6 (100 MHz).

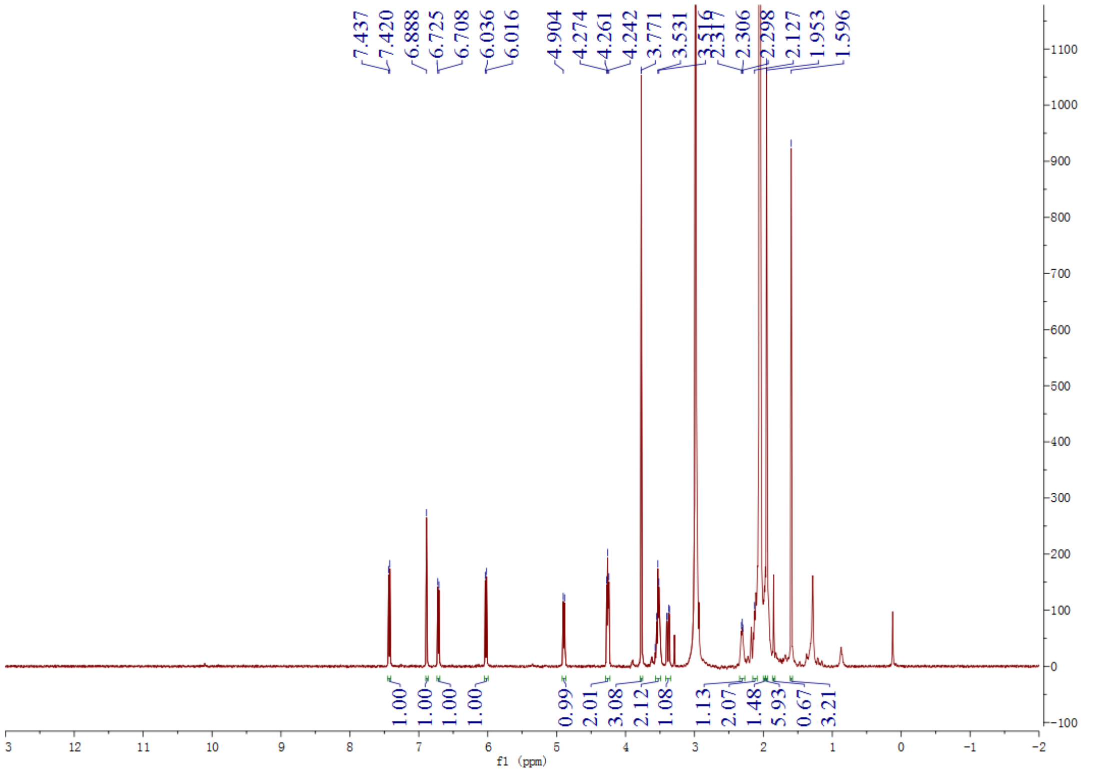

Figure S61. ^{1}H NMR spectrum of compound **14** in acetone-d_6 (500 MHz).

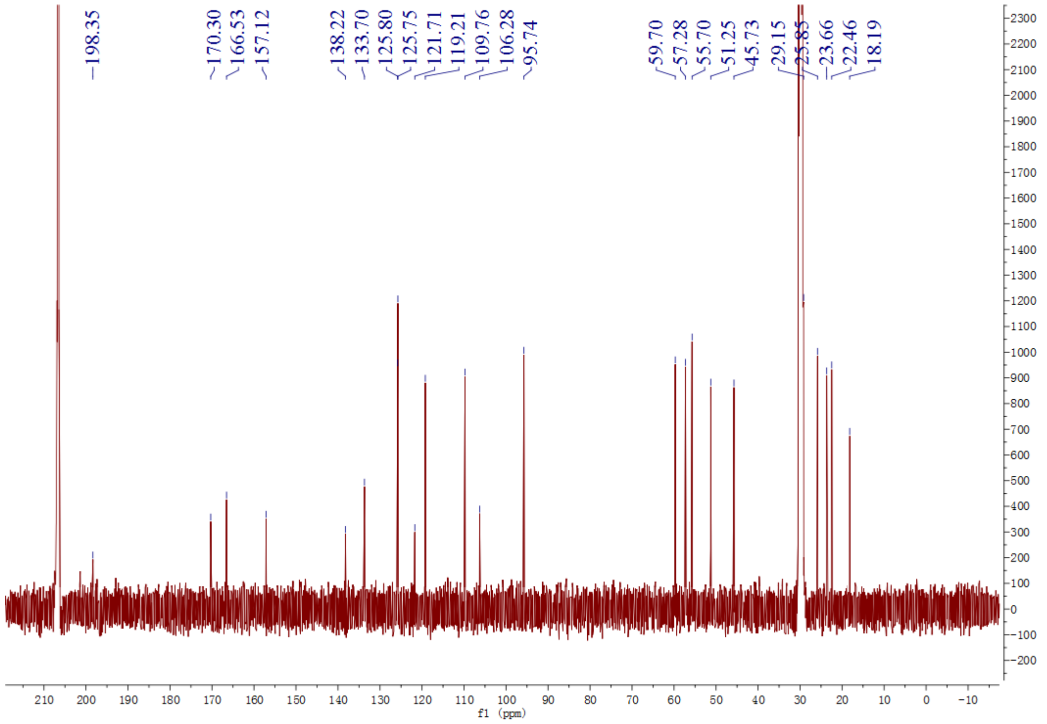

Figure S62. ^{13}C NMR spectrum of compound **14** in acetone-d_6 (125 MHz).

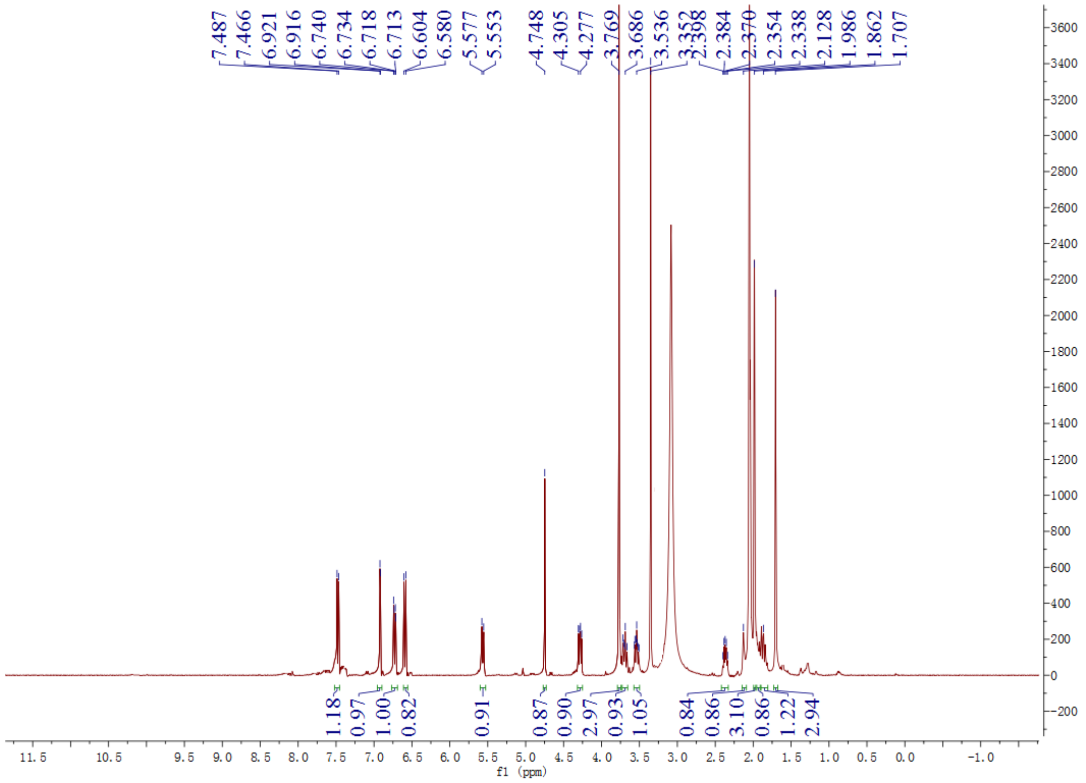

Figure S63. ^{1}H NMR spectrum of compound **15** in acetone-d_6 (400 MHz).

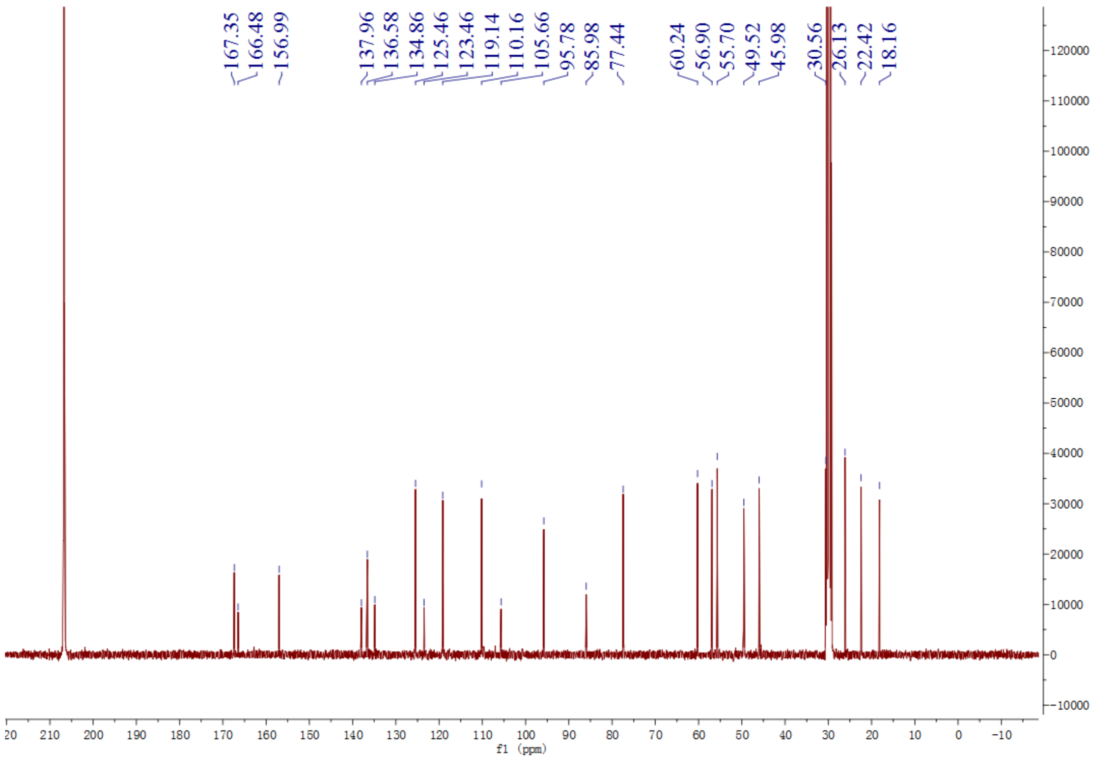

Figure S64. ^{13}C NMR spectrum of compound **15** in acetone-d_6 (100 MHz).

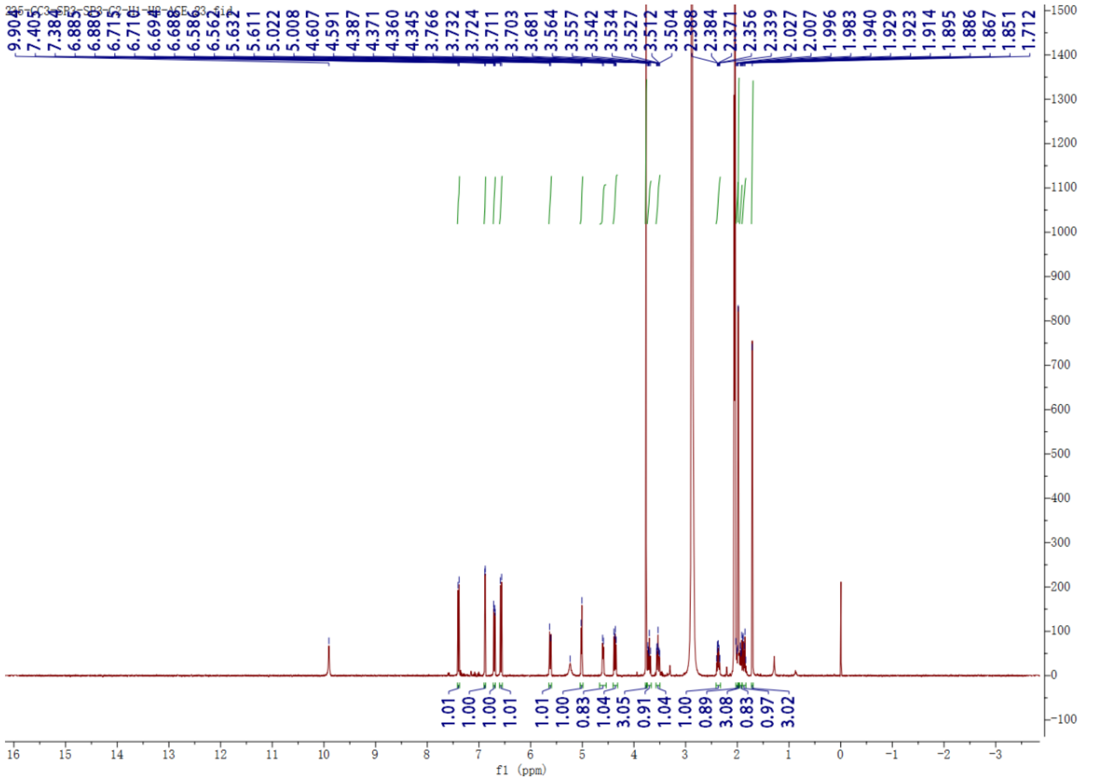

Figure S65. ^{1}H NMR spectrum of compound **16** in acetone-d_6 (400 MHz).

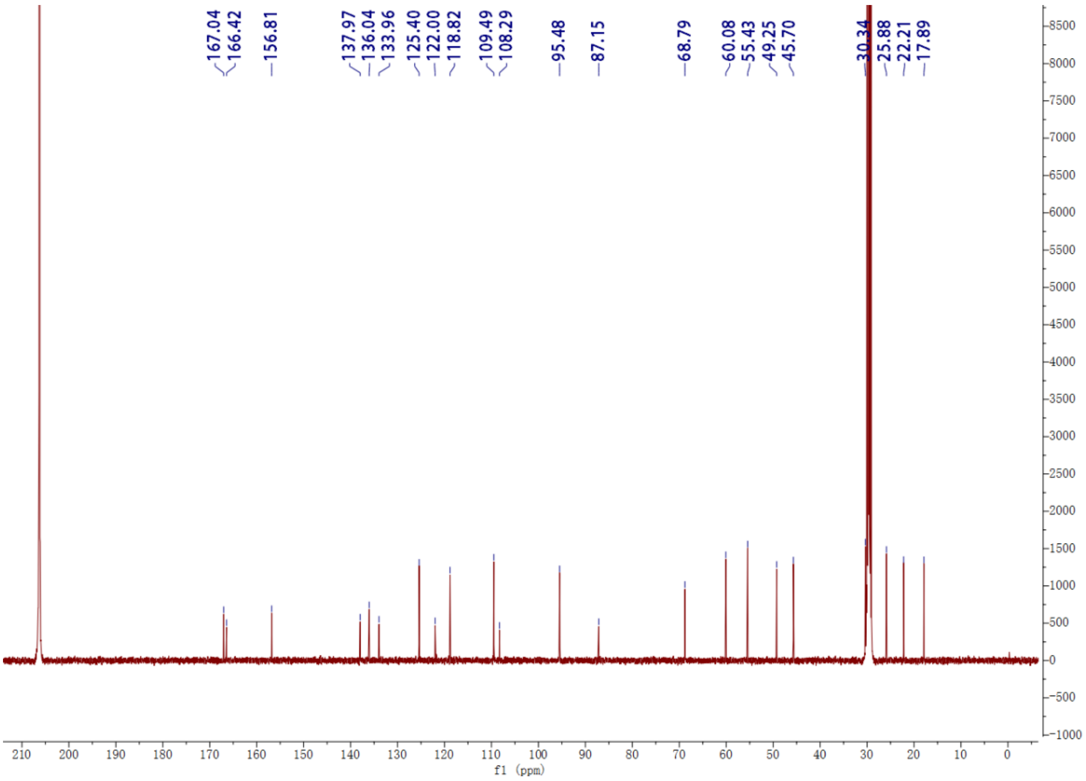

Figure S66. ^{13}C NMR spectrum of compound **16** in acetone-d_6 (125 MHz).

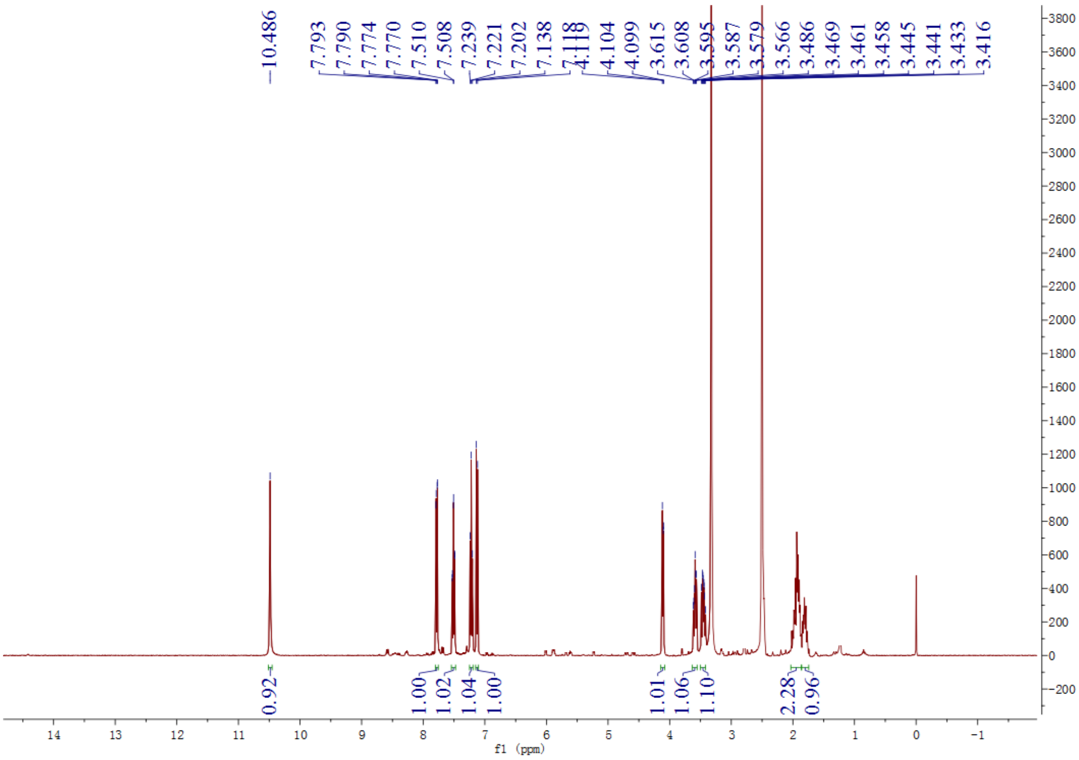

Figure S67. ^{1}H NMR spectrum of compound **17** in DMSO-d_6 (400 MHz).

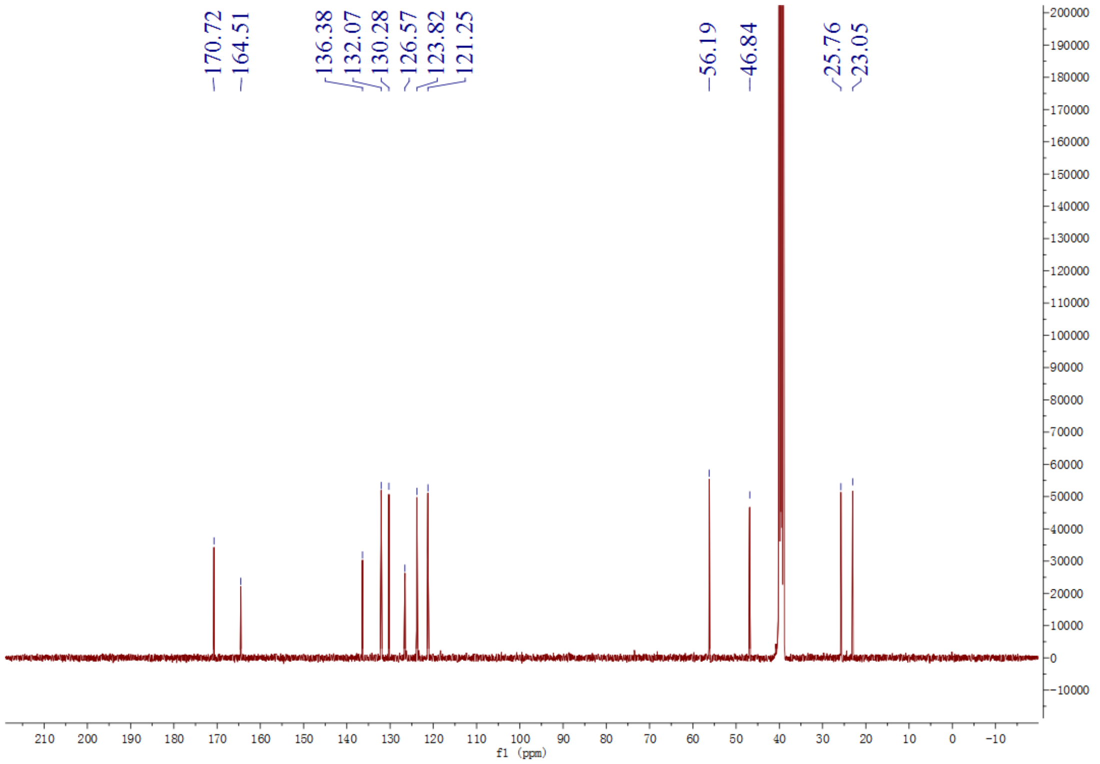

Figure S68. ^{13}C NMR spectrum of compound **17** in DMSO-d_6 (100 MHz).

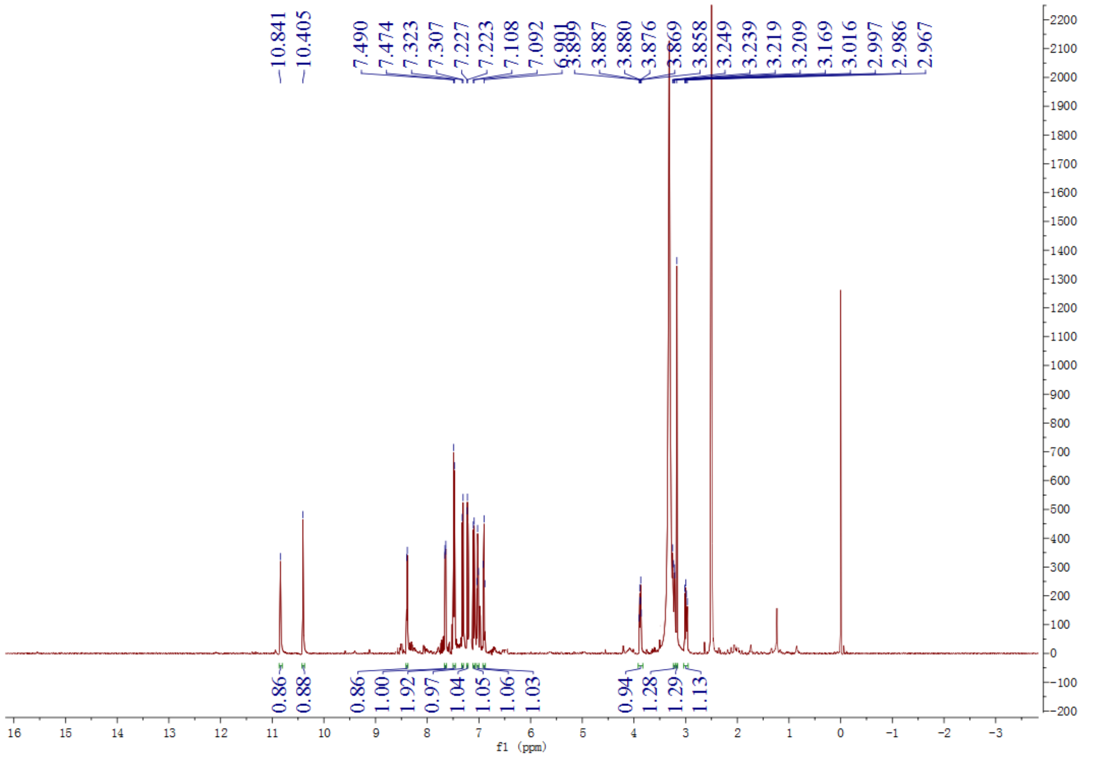

Figure S69. ^{1}H NMR spectrum of compound **18** in DMSO-d_6 (500 MHz).

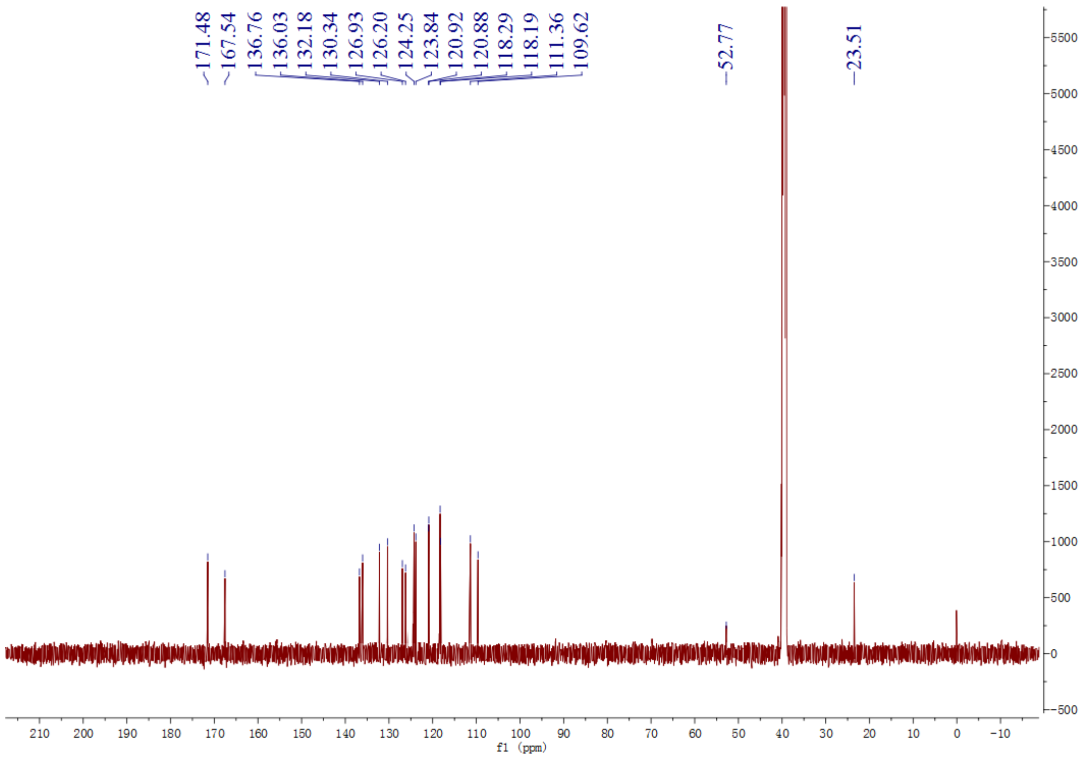

Figure S70. ^{13}C NMR spectrum of compound **18** in DMSO-d_6 (125 MHz).

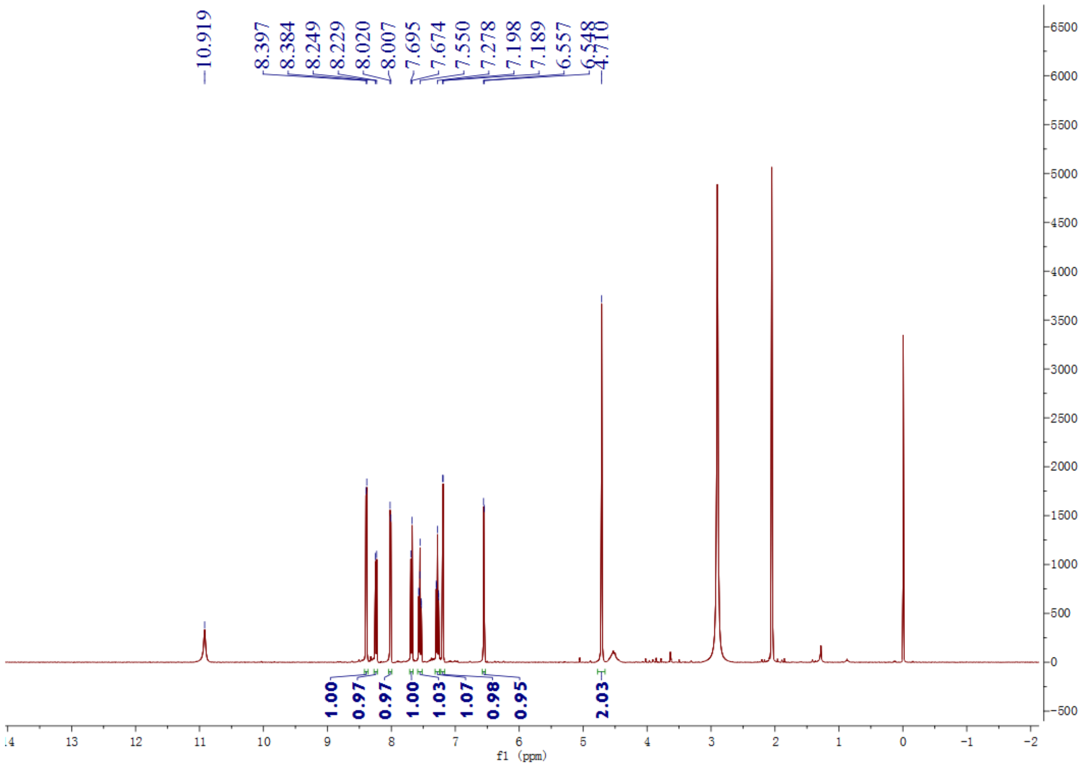

Figure S71. ^{1}H NMR spectrum of compound **19** in acetone-d_6 (400 MHz).

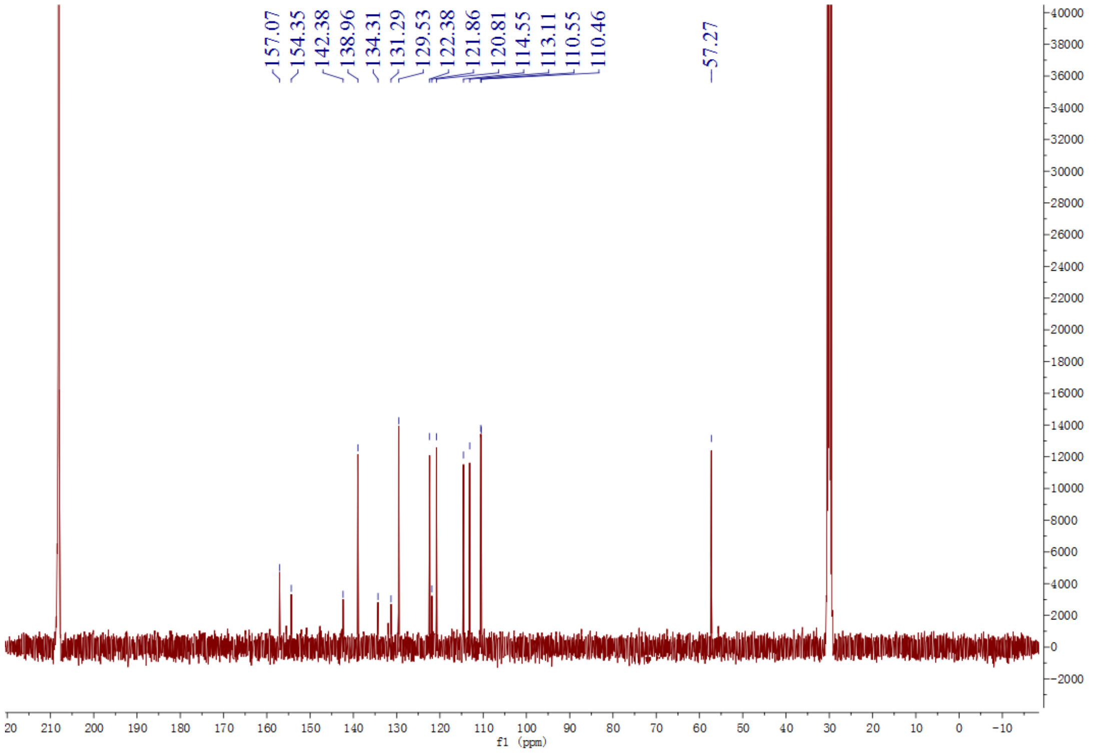

Figure S72. ^{13}C NMR spectrum of compound **19** in acetone-d_6 (100 MHz).

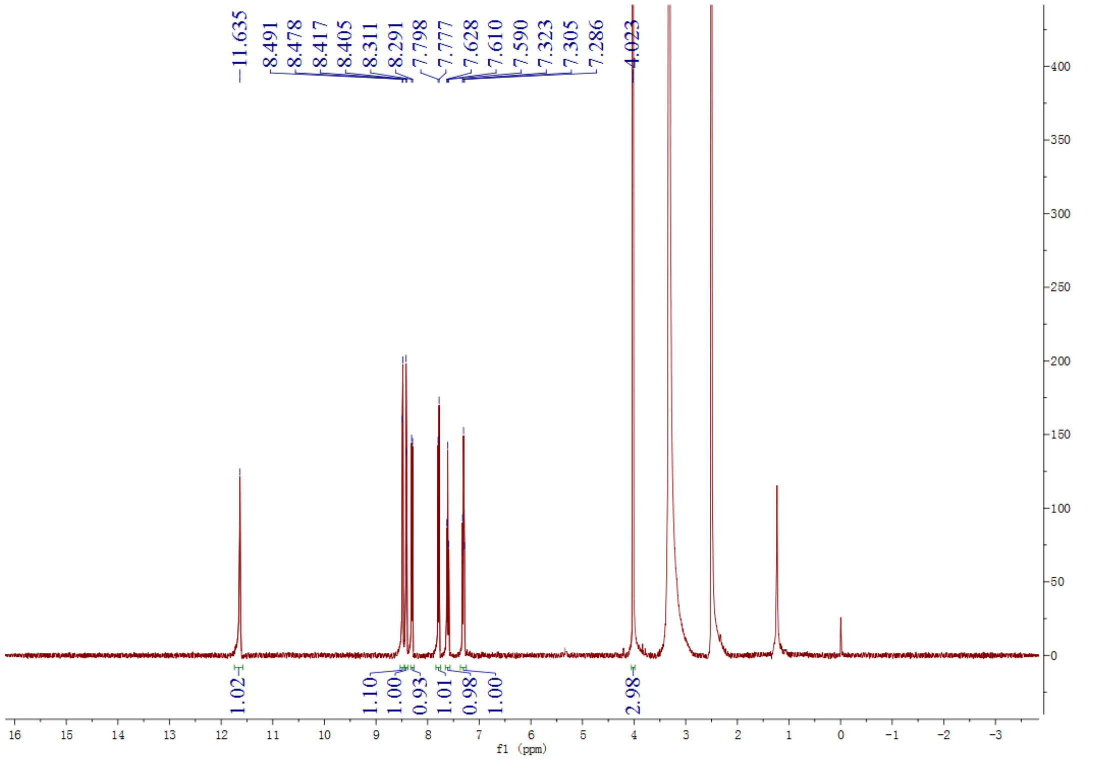

Figure S73. ^{1}H NMR spectrum of compound **20** in DMSO-d_6 (400 MHz).

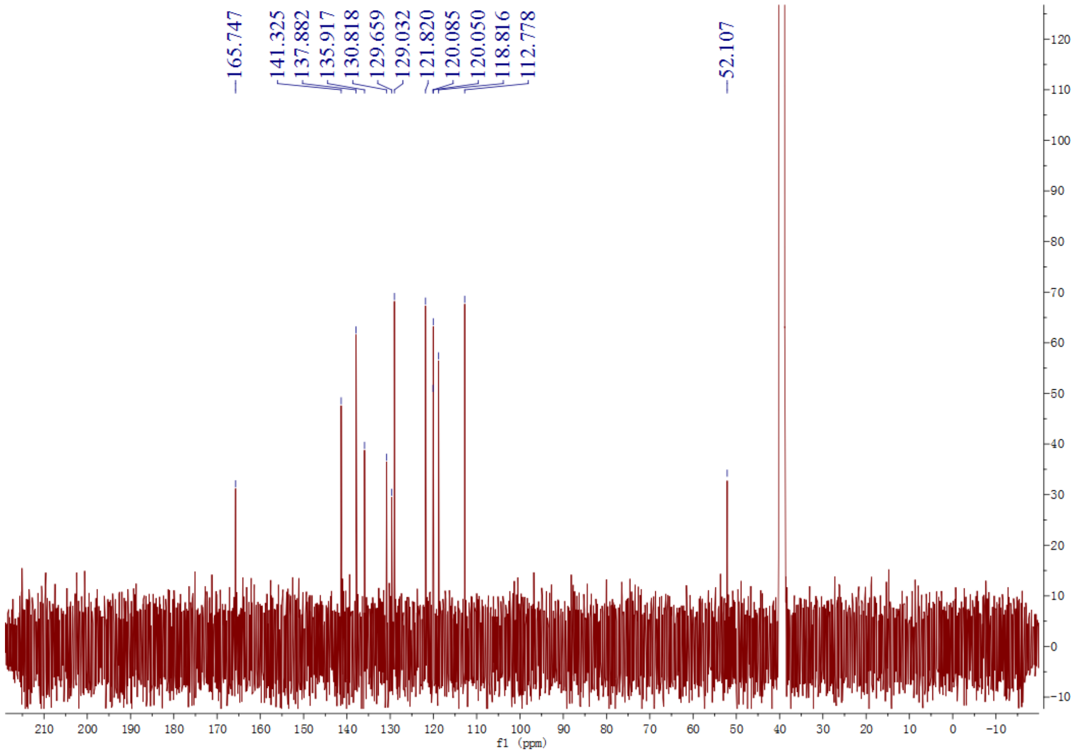

Figure S74. ^{13}C NMR spectrum of compound **20** in DMSO-d_6 (100 MHz).

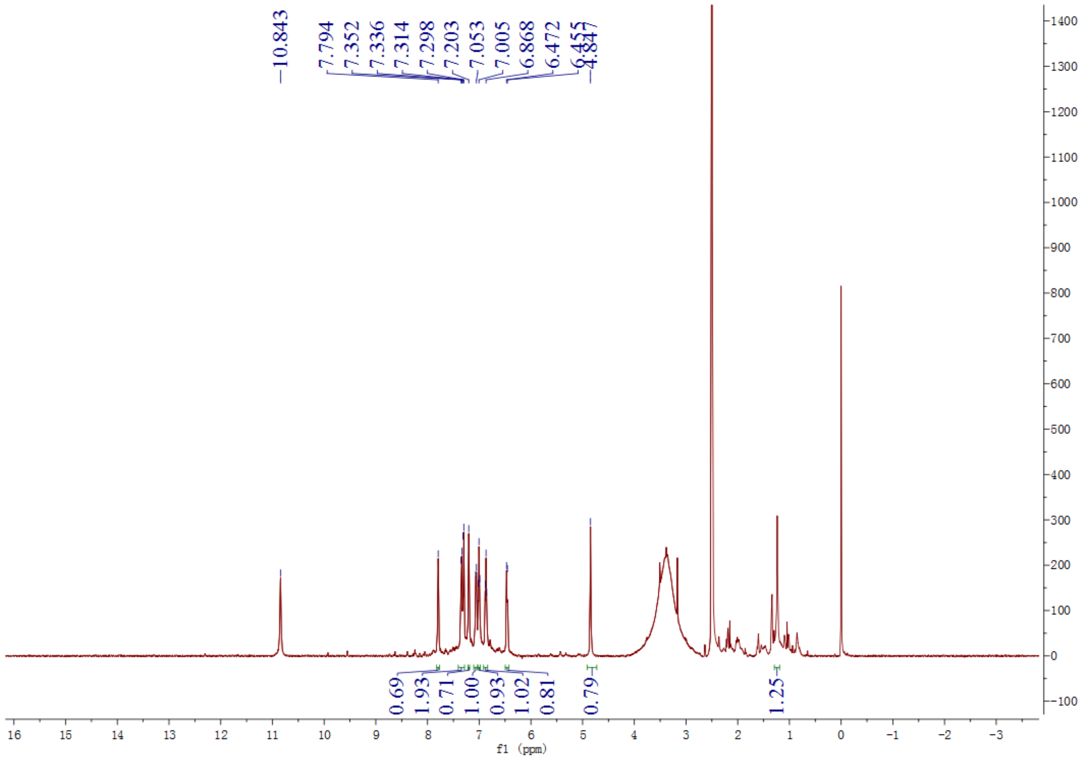

Figure S75. ^{1}H NMR spectrum of compound **21** in CD_3OD (500 MHz).

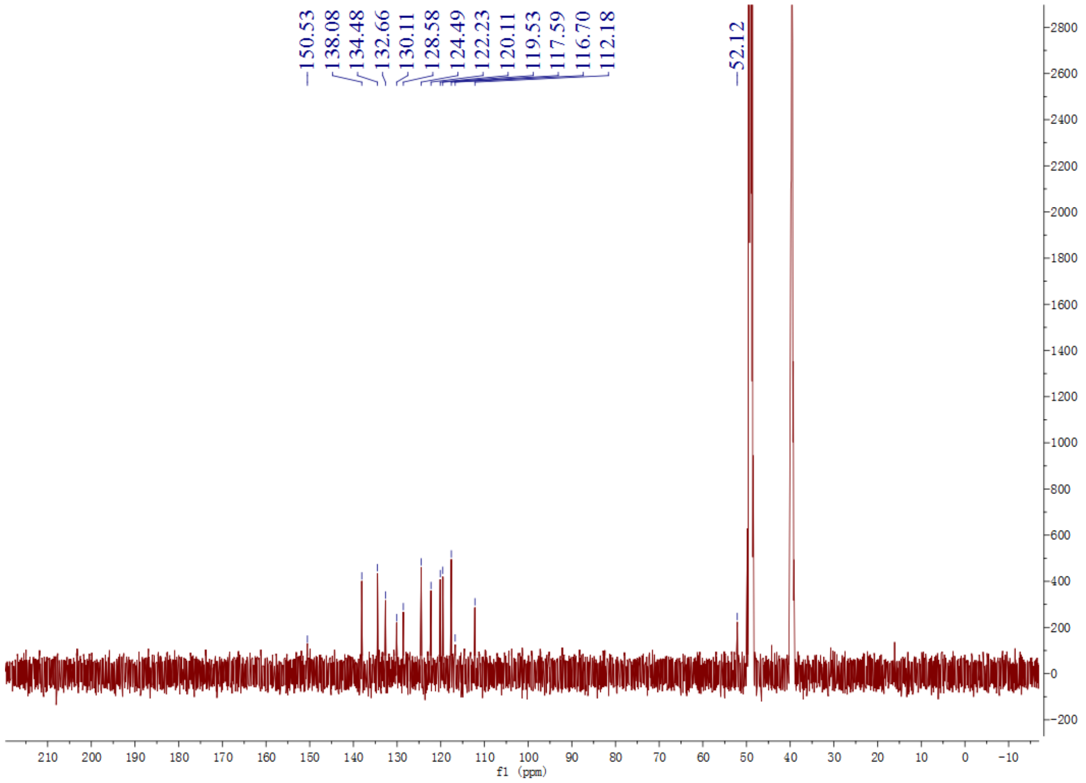

Figure S76. ^{13}C NMR spectrum of compound **21** in CD_3OD (125 MHz).
